# Fine mapping, introgression, and KASP marker development for powdery mildew resistance in watermelon using an interspecific RIL population (*Citrullus mucosospermus* × *C. lanatus*)

**DOI:** 10.1007/s00122-025-05079-4

**Published:** 2025-11-08

**Authors:** Rahul Kumar, Jennifer Ikerd, Raghupathy Karthikeyan, Chandrasekar Kousik

**Affiliations:** 1 U.S. Vegetable Laboratory, USDA ARS, Charleston, SC 29414 USA; 2 Agricultural Sciences Department, Clemson University, Charleston, SC 29634 USA

## Abstract

**Key message:**

A major QTL conferring powdery mildew resistance was fine mapped to a 54.8 kb region on chromosome 2 using an interspecific RIL population (*Citrullus mucosospermus* × *Citrullus lanatus*). Four co-segregating KASP markers were developed and validated across multiple populations, demonstrating their utility for marker-assisted selection.

**Abstract:**

Powdery mildew, caused by *Podosphaera xanthii*, is a major fungal disease that significantly affects watermelon production worldwide. Developing resistant cultivars through marker-assisted selection (MAS) offers an effective and sustainable strategy for disease management. In this study, a 54,772 bp quantitative trait locus (QTL) associated with powdery mildew resistance was mapped to chromosome 2 (30,111,475–30,166,247 bp) using an *F*_11_ recombinant inbred line (RIL) population derived from an interspecific cross between the resistant *C. mucosospermus* line USVL531-MDR and the susceptible *C. lanatus* line USVL677-PMS. Genetic analysis revealed that resistance is controlled by a single dominant gene, supported by a 3:1 segregation ratio observed in *F*_2_ populations. The mapped region harbored three lipoxygenase (*LOX*) genes and one 50S ribosomal protein L27-like gene. Four KASP markers were developed from SNPs located within four putative genes in the QTL region and were validated across multiple segregating populations, including the RIL (USVL531-MDR × USVL677-PMS) and two *F*_2_ populations (USVL531-MDR × ’Sugar Baby’ and PI 560003 × USVL677-PMS). These markers accurately differentiated resistant and susceptible individuals (*R*2 = 0.68–0.82) and exhibited 100% co-segregation with powdery mildew resistance in the RIL and two *F*_2_ populations, demonstrating their utility for MAS. The identified QTL and validated KASP markers will facilitate MAS for powdery mildew resistance breeding and enable future gene cloning work.

**Supplementary Information:**

The online version contains supplementary material available at 10.1007/s00122-025-05079-4.

## Introduction

Watermelon (*Citrullus lanatus*) is a widely cultivated cucurbit crop for human consumption. Rich in bioactive compounds such as lycopene, citrulline, and antioxidants, watermelon plays a vital role in promoting cardiovascular health, reducing oxidative stress, and supporting overall human wellness (Perkins-Veazie et al. 2005; Kousik et al. 2015; Maoto et al. 2019; Zamuz et al. 2021). Watermelon is cultivated on over 100,000 acres, generating an annual market value of $787 million in the USA (USDA NASS 2024). In 2023, Florida, Georgia, North Carolina, and South Carolina accounted for 58,400 acres of watermelon production, representing 56% of the total 103,900 acres cultivated in the USA. However, the weather conditions in these states create favorable environmental conditions for fungal pathogens such as *Podosphaera xanthii* (powdery mildew), *Didymella bryoniae* (gummy stem blight), *Fusarium oxysporum* f. sp. *niveum* (Fusarium wilt), and *Colletotrichum* species (anthracnose), which negatively impact watermelon yields and quality (Kousik et al. 2016).

Powdery mildew, caused by *P. xanthii*, is one of the major diseases affecting watermelon in the USA (Jarvis 1992; Keinath and DuBose 2004; McGrath 2017; Rennberger et al. 2018). Powdery mildew symptoms primarily appear on the leaves, stems, and occasionally fruits. These symptoms appear as white, powdery lesions, resulting from fungal colonization and sporulation (Cohen et al. 2004; Kousik et al. 2011; Keinath and DuBose 2012; McGrath 2017; Mandal et al. 2020). Powdery mildew reduces photosynthesis, resulting in reduction of fruit quality, yield, and market value (Matheron and Porchas 2013; Candido et al. 2014). Fungicides are commonly used to manage powdery mildew; however, their effectiveness progressively decreases due to the emergence of fungicide-resistant strains of *P. xanthii* (McGrath 2001, 2017; Rennberger et al. 2018). The issue is further exacerbated by the existence of various physiological races of *P. xanthii*. Seven physiological races of the cucurbit powdery mildew pathogen *P. xanthii* have been identified, using melon (*Cucumis melo*) differentials (McGrath et al. 1996; Pitrat et al. 1998; McGrath 2017). Four races of *P. xanthii* based on a set of bitter gourd (*Momordica charantia*) differentials have been classified in Asia (Dhillon et al. 2018). Similarly, two races (0 and 1) based on a set of watermelon differentials have been described (Kousik and Ikerd 2025). Kousik and Ikerd (2025) demonstrated that there was no correlation between the races classified based on melon and watermelon. Similarly, Tores et al. (2021) found that there was no correlation between the race classification based on watermelon differentials and melon differentials in Europe. Several other race classification systems have also been described for melon and other cucurbits (Lebeda et al. 2011, 2012, 2016, 2021, 2024). Because of the lack of differentials and fully resistant germplasm, physiological races of *P. xanthii* have not been clearly described for many cucurbit crops (Cohen et al. 2004; Kousik and Ikerd 2025). Because of the presence of multiple physiological races of the pathogen, development of durable resistant cultivar is the best strategy for managing powdery mildew in watermelon (McGrath 2017). So far, several powdery mildew-resistant sources have been identified in watermelon germplasm (Thomas et al. 2005; Davis et al. 2007; Tetteh et al. 2010; Kousik et al. 2018).

Breeding for disease resistance offers both environmental and economic benefits, minimizing the need for chemical fungicides and promoting sustainable agricultural practices. Natural genetic diversity existing within germplasm serve as valuable resources in breeding programs aimed at incorporating stresses-resistant genes (Kumar et al. 2013, 2023, 2025, Ben-Naim and Cohen 2015; Chugh et al. 2024). Furthermore, genetic variation can be artificially introduced through mutagenesis, transgenics, and genome editing (Li et al. 2022; Rana et al. 2022; Kumar et al. 2022; Liu et al. 2023; Tang et al. 2023). *Citrullus mucosospermus* (egusi watermelons) and *C. lanatus* (cultivated watermelons) are closely related species that diverged approximately 3.1 million years ago (Chomicki and Renner 2015). *C. mucosospermus* species have proven to be invaluable sources of disease resistance in watermelon, including powdery mildew, zucchini yellow mosaic virus, and phytophthora fruit rot (Guner et al. 2018; Kousik et al. 2023). A powdery mildew-resistant watermelon line USVL531-MDR (*C. mucosospermus*) was developed through six generations of screening and selection from plant introduction PI 494531 by the USDA-ARS U.S. Vegetable Laboratory (Kousik et al. 2023). However, unlike commercial red fleshed watermelon, USVL531-MDR exhibits several undesirable traits, such as white hard flesh, low sugar, bitter fruit taste, egusi-type seeds, and a hard rind (Kousik et al. 2023). These characteristics limit its direct use in commercial watermelon production and breeding. Molecular breeding can be helpful in handling linkage drags and selecting desirable traits. Thus, transferring powdery mildew resistance genes from USVL531-MDR into elite, high-yielding cultivars via marker-assisted selection (MAS) can be a highly efficient strategy for developing disease-resistant watermelons. However, so far, no quantitative trait loci (QTLs) mapping or KASP marker development for powdery mildew resistance has been reported in *C. mucosospermus*.

Previous studies have identified several QTL associated with powdery mildew resistance in *C. lanatus*. Deng et al. (2024) mapped a QTL to chromosome 2 containing the candidate gene *ClLOX*, which encodes a lipoxygenase involved in plant defense mechanisms. Similarly, Kumar et al. (2025) mapped a 570 kb QTL on chromosome 2 using the BSA-seq method. Furthermore, Kim et al. (2015) identified a major QTL on chromosome 2. Genome-wide association studies (GWAS) have also identified overlapping and adjacent regions on chromosomes 2, 3, 4, 7, and 9 that are linked to powdery mildew resistance (Wu et al. 2019; Mandal et al. 2020).

The objectives of this study were to map and develop Kompetitive Allele Specific PCR (KASP) markers for powdery mildew resistance in watermelon using an F_11_ recombinant inbred line (RIL) population, derived from a cross between *C. mucosospermus* resistant germplasm line (USVL531-MDR) and a *C. lanatus* susceptible line (USVL677-PMS). The KASP markers developed based on the *F*_11_ RIL population were then further validated in two different *F*_2_ populations.

## Material and methods

### Plant material and powdery mildew isolate

The study used an *F*_11_ population of 183 RILs, developed from an interspecific cross between USVL531-MDR (S6, derived from PI 494531) and USVL677-PMS (derived from PI 269677) for the QTL mapping (Kousik et al. 2023). Two *F*_2_ populations, USVL531-MDR × ’Sugar Baby’ and PI 560003 (*C. mucosospermus*) × USVL677-PMS, were also developed for the validation of KASP markers. USVL531-MDR and PI 560003 exhibit resistance to powdery mildew, while ‘USVL677-PMS’ and ‘Sugar Baby’ are susceptible. Seeds were sown in 2.54 cm square jiffy pots filled with Metro Mix (Sun Gro Horticulture, Bellevue, WA). Trays were placed on a heated mat at 27 °C for 4–5 days for uniform germination. Seedlings were grown for four weeks in a greenhouse before transplanting into the field.

### Field experiments

Field experiments were conducted during the summer (planted on May 19) and fall (planted on June 14) of 2023 in two different fields at the USDA U.S. Vegetable Laboratory (USVL) farm in Charleston, SC (32° 48′ 5″N, 80° 3′ 50″W). The experiments followed a randomized complete block design (RCBD) with two replications, and each plot consisted of five plants. Field plots consisted of 91 cm-wide, 20 cm-high raised beds covered with white plastic mulch, spaced 4.6 m apart. Before transplanting, row middles were treated with Roundup Pro (1.17 L/ha) and Dual Magnum (1.17 L/ha) herbicides for weed management. Throughout the growing season, weeds within plots were managed by hand weeding while row middles were maintained using spot applications of Roundup. The field was irrigated by drip irrigation system weekly or as needed, with a single tape placed 2.5 cm below the surface (Kemble et al. 2014). The field was fertigated through the drip tape using 10-0-10 PLUS liquid fertilizer (Possum’s, Charleston, SC). Pesticides were not applied to control diseases and insects.

### *Phenotyping F*_*2*_* populations in a Growth Chamber*

Two *F*_2_ populations, USVL531-MDR × ’Sugar Baby’ and PI 560003 × USVL677-PMS, were screened for powdery mildew resistance in a growth chamber under controlled conditions (25 °C, relative humidity 60–65%, photoperiod 16 h, light intensity 120 μmol m − 2 s − 1). Powdery mildew (*P. xanthii*) isolate B108ML collected from watermelon in Charleston, SC and classified as Race 1 based on watermelon differentials was used for inoculation (Kousik and Ikerd 2025). The isolate B108ML was classified as Race 1 based on melon differentials and can be designated as Race 1W based on the earlier system (Davis et al. 2007) where a ‘W’ was added after the 1 as at that time a race classification system based on watermelon did not exist. However, Kousik and Ikerd (2025) have described PM isolates that behaved as Race 1 based on watermelon differentials, but then as Race 2 based on melon differentials as they could also infect the melon cultivar PMR-45. The B108ML isolate used in this study did not cause symptoms on the melon differential PMR-45, but infected the melon differential Iran H, and hence it also gets classified as a melon Race 1. For further detailed description please refer to Kousik and Ikerd (2025). The isolate was routinely maintained on Early Prolific Straight Neck (EPSN) squash plants in a growth chamber as described previously (Kousik et al. 2018). Plants were inoculated with a conidial suspension as described by Kousik et al. (2018). Briefly, *P. xanthii* conidia were collected from the powdery mildew-infected squash leaves. A conidial suspension at a concentration of 2 × 104 conidia/mL was prepared in water containing 0.02% Tween 20. The inoculum suspension was then sprayed onto four-week-old plants using a handheld pump sprayer. Plants were rated for powdery mildew development after 14 days of inoculation using a 0–10 scale as described by Kousik et al. (2018).

### Powdery mildew rating

Plants were rated for powdery mildew resistance using a 0–10 rating system (Kousik et al. 2018). The scale ranged from 0, indicating no visible powdery mildew symptoms, to 10, representing complete coverage of the leaf surface with abundant conidia and leaf dying or dead. Intermediate ratings included: 1, characterized by very little scattered mycelial growth on leaves or cotyledons with few to no visible conidia affecting 1–3% of the leaf area; 2, where 4–6% of the leaf area showed powdery mildew symptoms with sparse development of conidia; 3, with 7–12% of the leaf area affected; 4, showing 13–25% coverage; 5, with 26–50% of the leaf area affected; 6, representing 51–75% coverage with abundant conidia; 7, where 76–87% of the leaf area was covered; 8, with 88–94% of the leaf area affected; and 9, indicating 95–97% of the leaf area was covered with powdery mildew. Disease ratings were recorded for Trial 1 on June 2, June 9, and June 16. Assessments for trial 2 were conducted on June 23, June 30, and July 7. Ratings were focused on older lower leaves, where symptoms of powdery mildew were most prominent, allowing for a consistent and reliable evaluation of disease severity.

### DNA extraction, sample preparation, and whole-genome resequencing

Leaf samples from the plants were collected before transplanting to the field, immediately frozen in liquid nitrogen, and stored at − 80 °C. Genomic DNA was extracted from the leaf tissues using the DNeasy Plant Mini Kit (QIAGEN, USA) according to the manufacturer’s protocol. DNA concentration and quality were determined using a Qubit fluorometer with the Qubit dsDNA HS assay kit (Life Technologies, CA, USA) and agarose gel (1%) electrophoresis, respectively. Whole-genome resequencing was performed using the Illumina HiSeq 2500 platform (Illumina, Inc., USA) to generate 150 bp paired-end reads. Libraries were prepared for sequencing using a standard Illumina protocol, and sequencing was conducted to achieve 30X depth across the genome.

### Raw data quality assessment and preprocessing

The quality of the raw sequencing data was evaluated using FastQC version 0.12.1 (Andrews 2010). Subsequently, Trimmomatic version 0.39 was used to remove adapter sequences and low-quality reads (Bolger et al. 2014). Cleaned PE reads were aligned to the ‘Charleston Gray’ watermelon reference genome (Wu et al. 2019) using the MEM algorithm of BWA-MEM2 version 2.1 (Li and Durbin 2009). The alignment produced SAM files, which were subsequently converted to BAM files and sorted using SAMtools version 0.1.8 (Li et al. 2009). Read groups were assigned using Picard Tools version 2.18.7 to differentiate among samples.

### Variant discovery and filtering

Variant discovery was conducted following the GATK Best Practices Workflow (DePristo et al. 2011; Van der Auwera et al. 2013) using GATK version 3.6 (McKenna et al. 2010). Initial variant calling identified single nucleotide polymorphisms (SNPs) and indels across the genome, which were subsequently filtered to retain high-quality markers for downstream analyses. SNP filtering was performed using VCFtools version 0.1.15 (Danecek et al. 2011), applying rigorous criteria to ensure the accuracy and reliability of the dataset. SNPs with a minor allele frequency (MAF) of less than 0.05, more than 10% missing data, or multiallelic sites with more than two alleles were excluded. After applying these filters, no accessions from the RIL population were excluded, resulting 407,963 SNPs in a final dataset optimized for QTL mapping. This stringent filtering ensured a robust dataset suitable for downstream analyses.

### Linkage map and QTL mapping

QTL analysis was performed using genotypic data along with mapping information and powdery mildew resistance data. QTL analysis was done using the *R*/qtl2 package (v0.24) (Broman et al. 2019), applying a linear mixed model with kinship as a random effect to correct for genetic relatedness among individuals. The leave-one-chromosome-out (LOCO) approach was used to control for population structure without sacrificing mapping power. Genotype probabilities were calculated using the appropriate cross type (‘riself’) for the recombinant inbred line population. To determine the statistical significance of detected QTL, a genome-wide permutation test with 10,000 iterations was performed. QTL were considered significant if the permutation-based *P*-value was less than 0.05. For each significant locus, the 95% Bayesian credible interval was used to define the QTL support interval.

A high-density linkage map was constructed in *R*/qtl v1.70 using a riself cross type. Markers were assigned to 11 linkage groups, pairwise recombination fractions were estimated, and genetic distances were derived using the Kosambi function. Co-located markers were collapsed to single positions for interval statistics, with genome- and chromosome-level lengths, mean spacing, and largest gaps being computed from adjacent cM distances. Segregation distortion was assessed per marker through Chi-square testing against the expected 1:1 ratio, with Benjamini–Hochberg FDR control applied at *q* = 0.05.

### KASP marker

KASP markers were developed based on SNPs identified within the mapped genomic regions associated with powdery mildew resistance. Candidate DNA and protein sequences from resistant and susceptible genotypes were aligned using NCBI BLAST tool (https://blast.ncbi.nlm.nih.gov/Blast.cgi). Two allele-specific forward primers and one common reverse primer were designed for the SNP located in the targeted genes. The genotyping reactions were performed using the Agilent AriaMx Real-Time PCR System (Agilent Technologies) in 96-well plates. Each reaction consisted of a 10 μL mixture, which included 5 μL of KASP 2 × master mix, 50 ng of genomic DNA template, and 0.17 μM of the primer mixture. The thermal cycling conditions for KASP genotyping began with an initial denaturation step at 94 °C for 15 min. This was followed by 10 touchdown cycles, each comprising 95 °C for 20 s and annealing at 65 °C for 60 s, with a temperature decrement of 0.8 °C per cycle. Once the final annealing temperature of 56 °C was reached, 26 additional amplification cycles were conducted, involving denaturation at 94 °C for 20 s and annealing at 57 °C for 60 s. Fluorescence signals from the amplified PCR products were detected and analyzed to differentiate between alleles, enabling the identification of polymorphic markers. The resulting genotype data were visualized using a Stratagene Mx3005P instrument (Agilent Technologies, Santa Clara, CA), which produced distinct allele clusters for each SNP, ensuring accurate marker differentiation.

### Statistical analysis

A Chi-square test (*χ*^2^) was performed to evaluate the goodness-of-fit between the observed segregation ratios and expected Mendelian inheritance patterns, such as the 3:1 ratio for resistance in* F*_2_ populations. Statistical significance was determined based on the corresponding degrees of freedom (df) and *P*-values. The coefficient of determination (*R*2) was calculated to determine the proportion of phenotypic variation explained by the KASP markers. Data visualization was performed in R version 4.5.0 using the ggplot2 package, including frequency distributions, violin plots, and scatter plots to assess phenotypic variation and inter-trial correlation in powdery mildew ratings.

## Results

### Phenotyping of RIL population for powdery mildew resistance

**Fig. 1:**
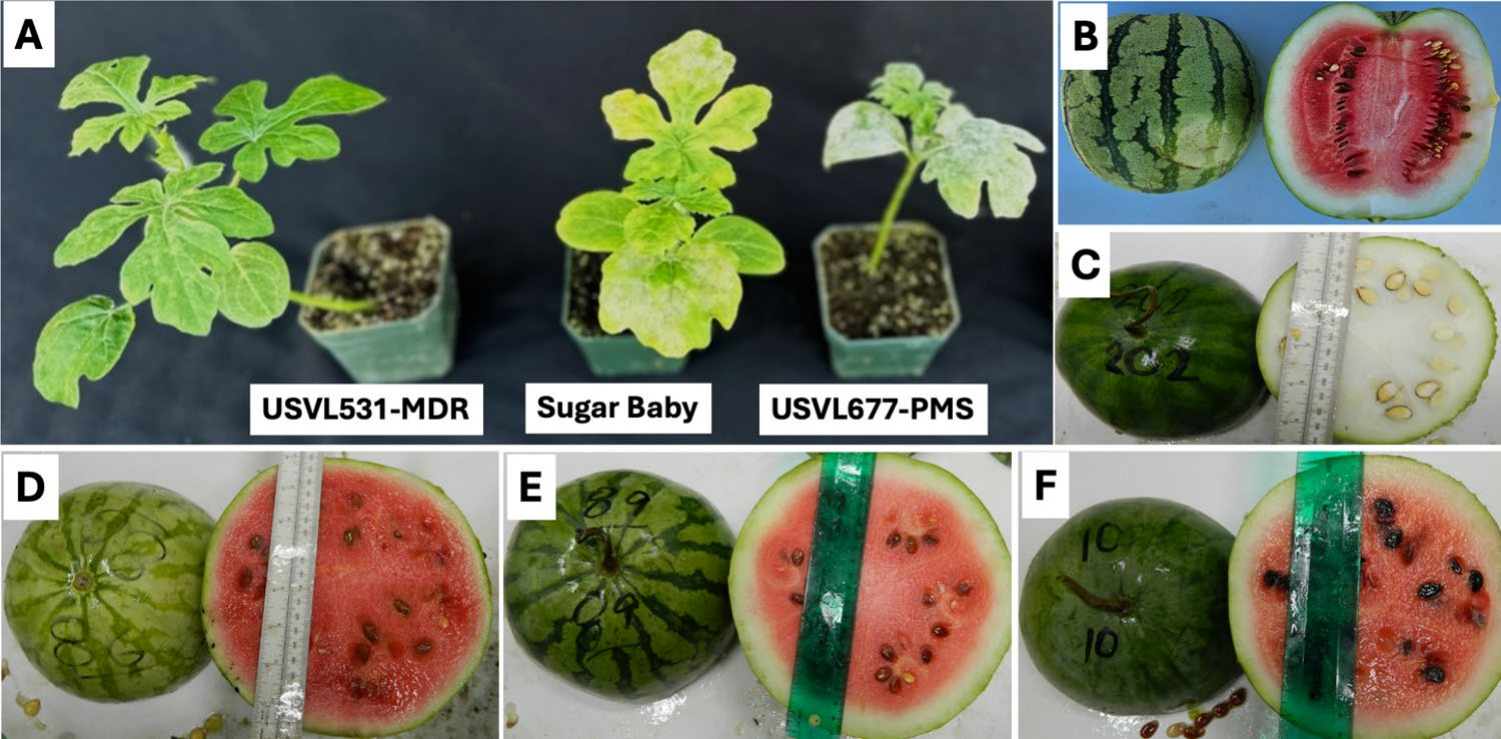
Phenotyping of plants for powdery mildew resistance and fruit characteristics. **A** Evaluation of *F*_2_ populations for powdery mildew resistance in the growth chamber. USVL531-MDR exhibited strong resistance, while the susceptible parental genotypes ‘Sugar Baby’ and USVL677-PMS showed visible powdery mildew symptoms. **(B–F)** Phenotyping of fruits harvested from the field. **B** Fruit phenotype of USVL677-PMS showing light green rind, red edible flesh, firm texture, brown non-egusi seeds, and a non-bitter taste. **C** Fruit phenotype of the resistant parent USVL531-MDR, characterized by dark green rind, hard non-edible white flesh, white egusi-type seeds, and a bitter taste. **(D–F)** Fruit phenotypes of selected powdery mildew-resistant recombinant inbred lines (RILs) with red edible flesh, firm texture, non-egusi seeds and a non-bitter taste

**Fig. 2:**
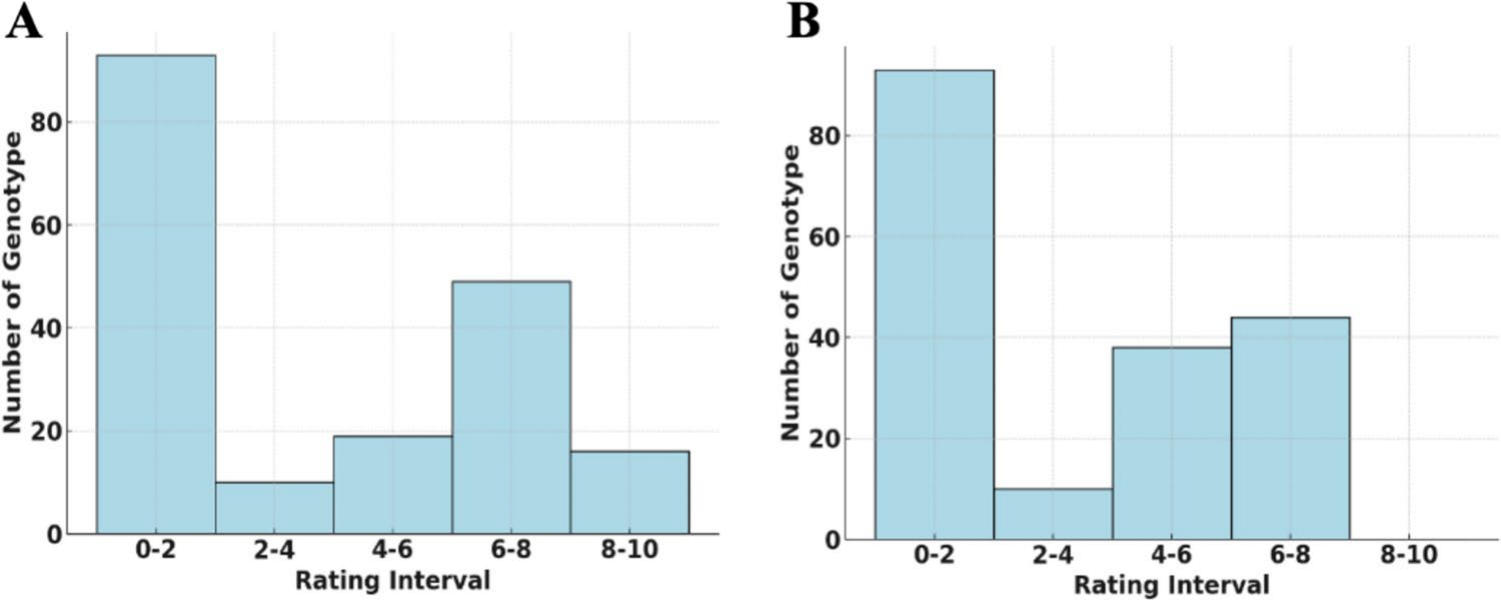
Phenotypic evaluation of RIL population for powdery mildew under field conditions. **A** Frequency Distribution of Ratings in Trial 1: A distribution of resistance ratings (0–10) showing moderate variation among the RILs, indicating genetic diversity in powdery mildew resistance. **B** Frequency Distribution of Ratings in Trial 2: Similar to Trial 1, but with a slightly broader spread, highlighting environmental or trial-specific effects on resistance expression

The interspecific RIL population (*F*_11_) comprising 183 RILs was evaluated for powdery mildew resistance at two field locations at the U.S. vegetable Laboratory farm in Charleston, SC during the summer and fall of 2023. Powdery mildew infections occur naturally every year on watermelon in Charleston, SC, and hence, plants were not inoculated. Powdery mildew development was rapid and severe in both trials (Fig. 1). The appearance of powdery mildew on plants was confirmed by the presence of white *P. xanthii* conidia on the abaxial surface of lower leaves. Based on reaction of watermelon and melon differentials in the same field, the prevailing *P. xanthii* race in the area was classified as race 1 as has been observed in the area for over a decade (Kousik et al. 2017). USVL531-MDR consistently showed resistance (Average 0), whereas USVL677-PMS was susceptible (Average 7.5 and range 6–8) to powdery mildew during both seasons. Disease severity scores across the RIL population were skewed toward resistance in both trials suggesting dominant nature of disease resistance (Fig. 2A, B).

Analysis of phenotypic variation within the recombinant inbred line (RIL) population revealed significant differences in resistance ratings across both trials. In Trial 1, a highly significant effect of genotype was detected (*F* (183, 1434) = 39.56, *P* < 0.01) with a total sum of squares of 17,732 and a mean square error (MSE) of 2.41. In Trial 2, the variation among RILs remained highly significant (*F* (183, 1482) = 12.03, *P* < 0.01) with a total sum of squares of 10,389 and an MSE of 4.64. These results demonstrate a significant genetic diversity within the RIL population, with Trial 1 showing a broader range of phenotypic responses compared to Trial 2. When data from both trials were combined, ANOVA results highlighted the significant contribution of genetic variation among RILs (*F* (183, 2916) = 38.71, p < 0.01). The mean resistance ratings varied significantly across replicates and trials. In Trial 1, replicate 1 showed average ratings of 3.88 (Rating 1), 2.48 (Rating 2), and 2.97 (Rating 3), while replicate 2 recorded averages of 3.30, 3.37, and 3.32 for the same ratings. In Trial 2, replicate 1 had average ratings of 3.25, 3.68, and 1.73, while replicate 2 recorded 3.28, 3.01, and 1.83. Broad-sense heritability estimates revealed that 66.57% of the phenotypic variation was attributable to genetic factors, reflecting the strong influence of genetic makeup on resistance. The repeatability of the resistance ratings across trials was estimated at 79.93%, indicating high consistency in performance. Several RILs exhibiting powdery mildew resistance, red edible flesh, firm texture, non-egusi seeds, and a non-bitter taste, were identified for further evaluation before releasing resistant lines (Fig. 1C–F).

### Identification of a major QTL for powdery mildew resistance on chromosome 2

**Fig. 3:**
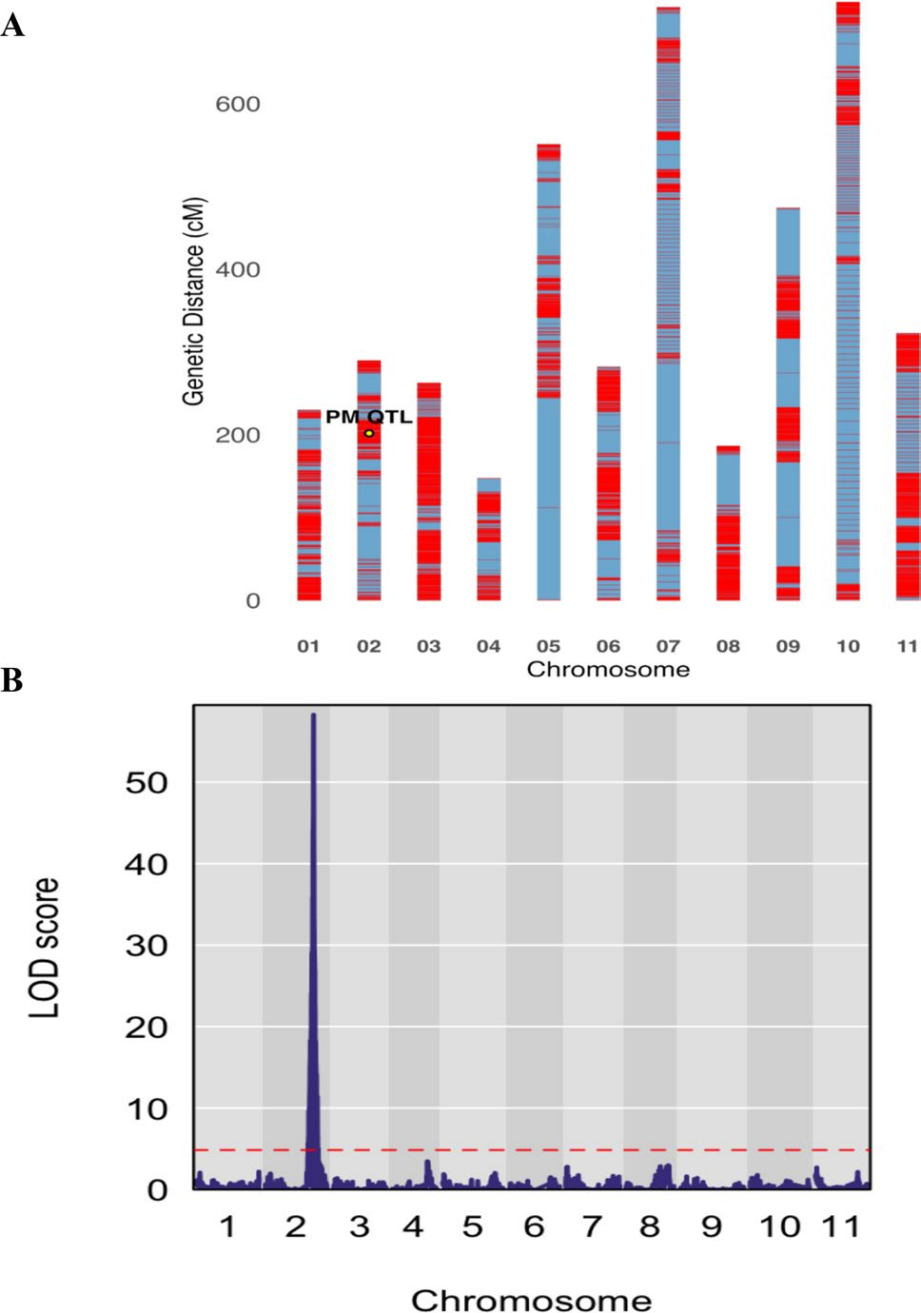
Linkage map and QTL mapping of powdery mildew resistance using a RIL population and whole-genome resequencing. **A** A high-density linkage map was constructed in *R*/qtl v1.70 using a riself cross type. **B** A major QTL was identified on chromosome 2 with a peak LOD score of 59 using *R*/qtl2, providing strong evidence for its association with powdery mildew resistance

The parental lines, USVL531-MDR and USVL677-PMS, along with 183 RILs, were re-sequenced to get ~ 30 × genome coverage for each line. Post-alignment quality control and variant calling resulted in the identification of 407,963 single nucleotide polymorphisms (SNPs) distributed across the genome. A high-density linkage map, constructed by *R*/qtl, covers 4,184 cM. The average distance between markers is 0.039 cM (0.0386 cM when counting unique positions; Fig. 3A). Chromosome lengths range from 148 to 722 cM. The biggest single gaps are on chr05 (132 cM), chr07 (106 cM), chr09 (80.3 cM), chr08 (61.4 cM), and chr02 (40.1 cM). No segregation distortion was detected (*P* < 0.05 test or the FDR *q* < 0.05 test).

Based on the unique segregation pattern, 8687 SNPs were selected for the QTL mapping to identify genomic regions associated with resistance to powdery mildew. Using the qtl2 software package, genome-wide QTL mapping was performed with phenotypic data obtained from field evaluations across two independent trials. A major QTL for powdery mildew resistance was identified on chromosome 2, with a LOD score of 59, indicating strong statistical evidence for the linkage (Fig. 3B). The identified QTL covers a genomic region of approximately 116,178 bp (position: 30,050,069–30,166,247 bp) on chromosome 2 and accounts for 82% of the phenotypic variance observed in the RIL population. This region includes six genes, of which five are lipoxygenases (ClCG02G015690, ClCG02G015700, ClCG02G015710, ClCG02G015730, and ClCG02G015750), along with the 50S ribosomal protein L27-like gene (ClCG02G015720).

### Development and validation of KASP markers for powdery mildew resistance in watermelon

**Fig. 4:**
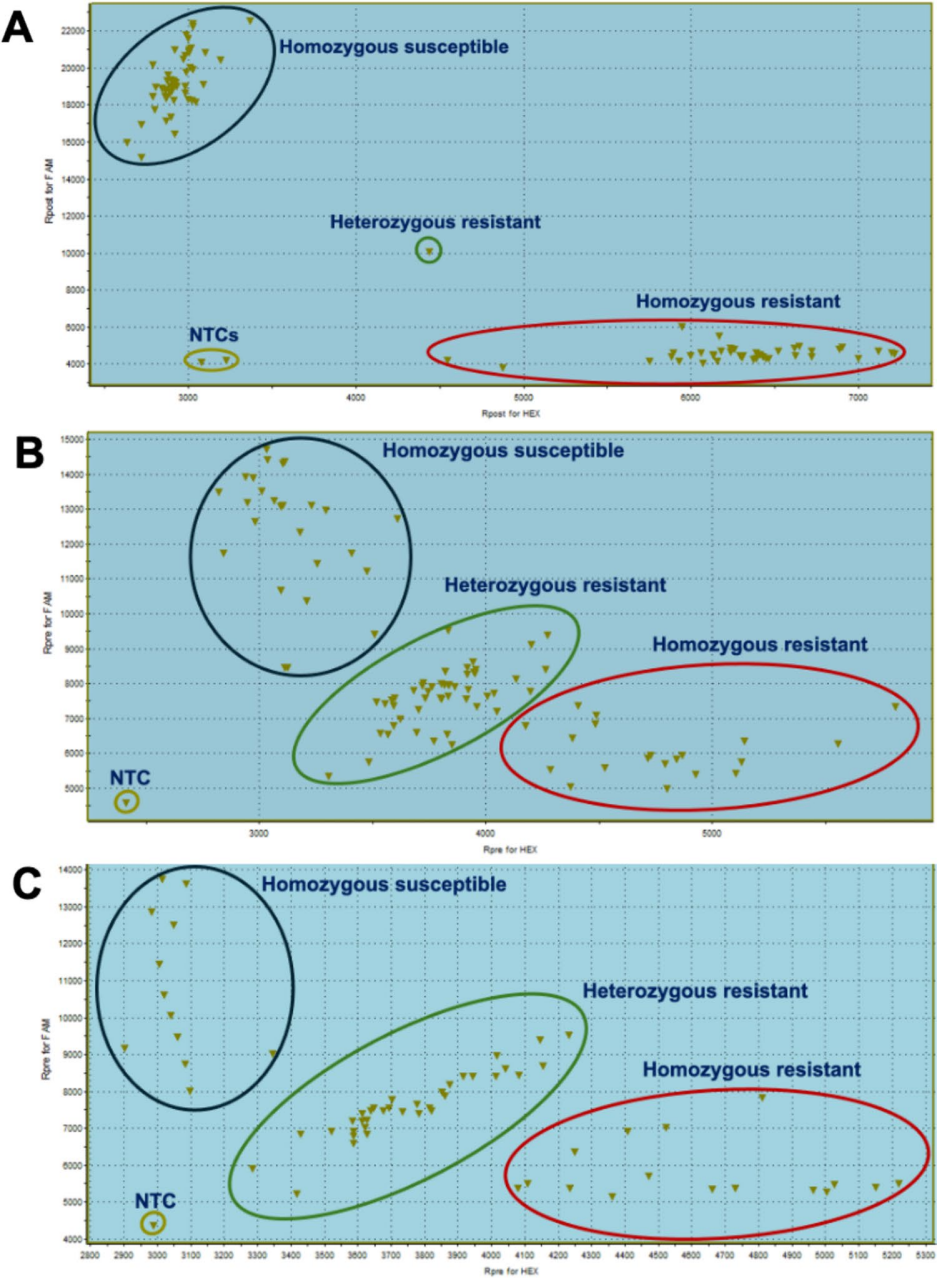
Development and validation of the CMPMR-5 KASP marker for marker-assisted selection (MAS) of powdery mildew resistance in watermelon. The CMPMR-730 marker was used to genotype a RIL population (**A**), an F_2_ population from PI 560003 × USVL677-PMS (**B**), and an F_2_ population derived from USVL531-MDR × ’Sugar Baby’. The KASP assay successfully distinguished homozygous resistant, heterozygous, and homozygous susceptible individuals. A strong correlation was observed between genotypic classes and powdery mildew resistance phenotypes (*R*2 = 0.68–0.82), indicating effectiveness of the marker for MAS

KASP primers CMPMR-1 to CMPMR-6 were designed for genotyping the RIL population, targeting the *LOX* genes ClCG02G015690*,* ClCG02G015700*,* ClCG02G015710*,* ClCG02G015730*,* and ClCG02G015750*,* as well as the 50S ribosomal protein L27-like gene ClCG02G015720 (Table 1). The KASP markers effectively distinguished homozygous resistant, homozygous susceptible, and heterozygous RILs (three (1.64%) heterozygous were observed) (Fig. 4). CMPMR-3, CMPMR-4, CMPMR-5, and CMPMR-6 each explained 82% (R2) of the phenotypic variation in powdery mildew resistance and exhibited 100% linkage among themselves and with the resistance phenotype (Table 2). In contrast, CMPMR-1 and CMPMR-2 explained a slightly lower proportion (79% *R*2), likely due to a linkage break relative to the other four markers and the resistance phenotype. This finding suggests that ClCG02G015690 and ClCG02G015700, targeted by CMPMR-1 and CMPMR-2, are not involved in powdery mildew resistance. As a result, the candidate region was narrowed from 116,178 bp to a refined interval of 54,772 bp (30,111,475–30,166,247 bp). To validate the KASP marker, two F_2_ populations derived from the USVL531-MDR × ’Sugar Baby’ and PI 560003 × USVL677-PMS, were phenotypically evaluated for powdery mildew resistance in the growth chamber. Disease severity was observed 14 days after inoculation and rated using a 0–10 rating system. The susceptible parents, USVL677-PMS and ‘Sugar Baby’, exhibited severe symptoms (ratings > 7), characterized by prominent white powdery fungal growth on hypocotyls and true leaves (Table 3, Fig. 1). On the other hand, the resistant parent USVL531-MDR and PI 560003 exhibited high levels of resistance, and powdery mildew symptoms were barely visible. Both *F*_2_ populations exhibited a Mendelian 3:1 segregation ratio for resistance and susceptibility to powdery mildew (Table 3). USVL531-MDR × ’Sugar Baby’ population (*N* = 61) exhibited a segregation ratio of 3:1, with 43 resistant and 18 susceptible progenies (*χ*2 = 0.34, *p* = 0.56). The PI 560003 × USVL677-PMS population (*N* = 96) exhibited a segregation ratio of 3:1, with 71 resistant and 25 susceptible progenies (*χ*2 = 0.056, *p* = 0.81). These results indicate that powdery mildew resistance is controlled by single dominant gene.

**Table 1:** List of KASP markers used for marker-assisted selection (MAS) along with their primer sequences

Marker name	Gene	Sequence
CMPMR-1	Lipoxygenase (*LOX*) (ClCG02G015690)	FAM forward—GAAGGTGACCAAGTTCATGCTAAGATTATTACAACAGTATAAGTTTCAAATATC
		Hex forward—GAAGGTCGGAGTCAACGGATTAAGATTATTACAACAGTATAAGTTTCAAATATT
		Reverse primer—CATACAAAACCTAACACATTGATTGTTAAT
CMPMR-2	Lipoxygenase (*LOX*) (ClCG02G015700)	FAM forward—GAAGGTGACCAAGTTCATGCTTTACATAAACCAAATAAATGTTTGGAAACTG
		Hex forward—GAAGGTCGGAGTCAACGGATTGTTTACATAAACCAAATAAATGTTTGGAAACTA
		Reverse primer—AACAAATACAAACATTTAATAACAAGATTT
CMPMR-3	Lipoxygenase (*LOX*) (ClCG02G015710)	FAM forward—GAAGGTGACCAAGTTCATGCTATAGTATGATGTTCATATTTCTTCAAGAAACAT
		Hex forward—GAAGGTCGGAGTCAACGGATTGTATGATGTTCATATTTCTTCAAGAAACAC
		Reverse primer—TTTGTTGTTAGAATTTGACTAAGAATTCAA
CMPMR-4	50S ribosomal protein L27-like gene (ClCG02G015720)	FAM forward—GAAGGTGACCAAGTTCATGCTGTTAAGTTTAAGTTAGATTTAGAAATATGTTCA
		Hex forward—GAAGGTCGGAGTCAACGGATTGTTAAGTTTAAGTTAGATTTAGAAATATGTTCG
		Reverse primer—CTCTGTGCTAATTGTTAGAATGCTAATTTA
CMPMR-5	Lipoxygenase (*LOX*) (ClCG02G015730)	FAM forward—GAAGGTGACCAAGTTCATGCTCGATTATGCTCTCTACAACGACCTT
		Hex forward—GAAGGTCGGAGTCAACGGATTATGCTCTCTACAACGACCTC
		Reverse primer—GGACGAGCGTATTGTGGACCTTTAT
CMPMR-6	Lipoxygenase (*LOX*) (ClCG02G015750)	FAM forward—GAAGGTGACCAAGTTCATGCTTCAAATAAATTGATCACTAACTAACGGC
		Hex forward—GAAGGTCGGAGTCAACGGATTCTTCAAATAAATTGATCACTAACTAACGGT
		Reverse primer—AGGTTGCATTAATACTCTTAGTCTTACAAA

**Table 2:** Phenotypic variation explained (*R*^2^) by KASP markers in three populations: *F*_11_ -USVL531-MDR × USVL677-PMS, *F*_2_ -USVL531-MDR × ’Sugar Baby’, and *F*_2_ -PI 560003 × USVL677-PMS

Cross	Marker	Phenotypic variation explained (*R*^2^, %)
F_11_-USVL531-MDR × USVL677-PMS	CMPMR-1	79
	CMPMR-2	79
	CMPMR-3	82
	CMPMR-4	82
	CMPMR-5	82
	CMPMR-6	82
F_2_-USVL531-MDR × ’Sugar Baby’	CMPMR-3	68
	ClPMR-5	68
F_2_-PI 560003 × USVL677-PMS	CMPMR-3	70
	ClPMR-5	70

**Table 3:** Segregation ratio of *F*_2_ progenies for powdery mildew resistance for experiments conducted in a growth chamber for the validation of KASP markers

Cross	Resistant	Susceptible	Total plants	Expected ratio	Df	*χ*^2^	*P* value
USVL531-MDR × ’Sugar Baby’	43	18	61	3:1	1	0.34	0.56
PI 560003 × USVL677-PMS	71	25	96	3:1	1	0.056	0.81
USVL531-MDR	8	0	8	–	–	–	–
PI 560003	8	0	8	–	–	–	–
USVL677-PMS	0	8	8	–	–	–	–
‘Sugar baby’	0	8	8	–	–	–	–

**Fig. 5:**
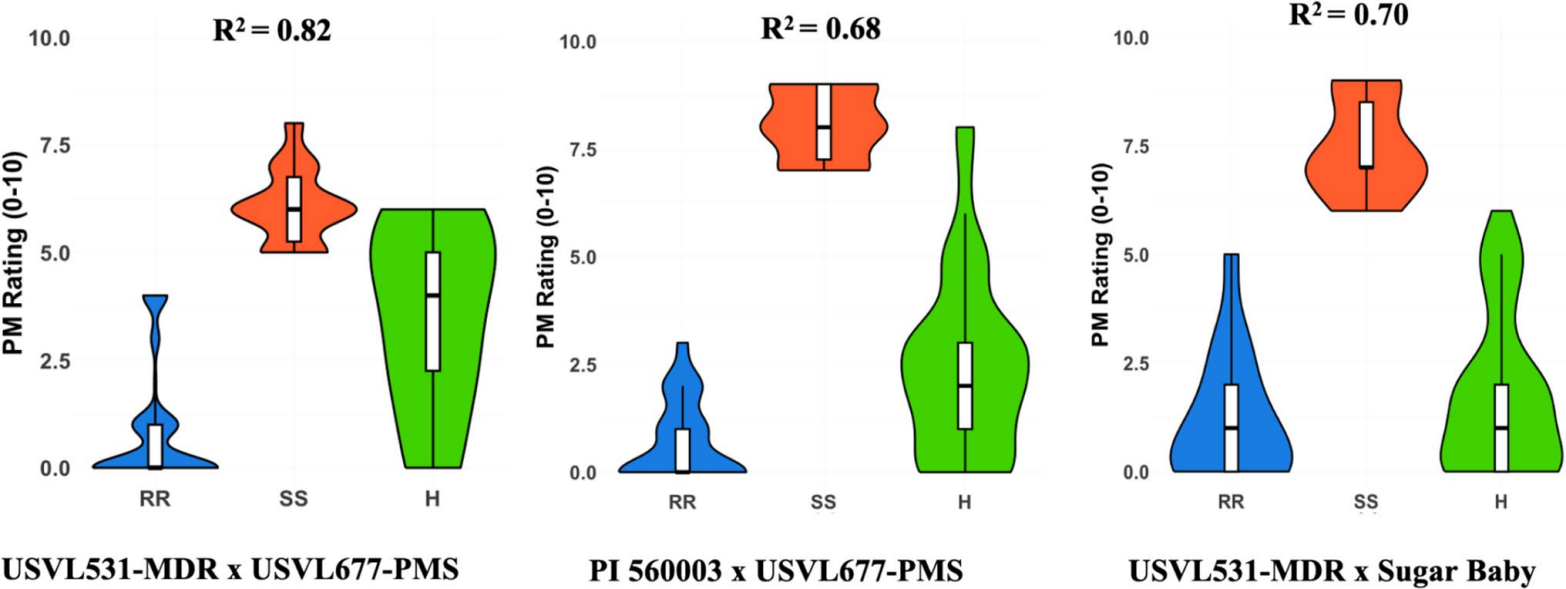
Violin plot showing powdery mildew rating of RIL and *F*_2_ populations linked with homozygous resistant (RR), homozygous susceptible (SS), and heterozygous (H) alleles genotyped by KASP markers. The width of each violin plot represents the probability density of powdery mildew ratings within each genotype, while the overlaid box plot depicts the median, interquartile range (IQR), and whiskers, indicating the central tendency and spread of the data. The results demonstrate that the RR (homozygous resistant) genotype exhibits lower powdery mildew ratings, confirming its association with resistance to powdery mildew. Conversely, the SS (homozygous susceptible) genotype displays higher powdery mildew ratings, reflecting its susceptibility, while the H genotype shows an intermediate distribution, suggesting partial resistance or additive genetic effects. The analysis was performed in R using ggplot2

CMPMR-3 and CMPMR-5 markers were used for the genotyping of *F*_2_ populations (Table 2). Both markers differentiated homozygous resistant, homozygous susceptible, and heterozygous progenies in both *F*_2_ populations. Both KASP markers explained 68% and 70% (*R*2) of the phenotypic variation in powdery mildew resistance in USVL531-MDR × ’Sugar Baby’ and PI 560003 × USVL677-PMS, *F*_2_ populations, respectively (Fig. 5, Table 2). CMPMR-3 marker distinguished genotypes better than CMPMR-5 marker. These results showed a strong linkage between CMPMR-3 to CMPMR-6 markers and powdery mildew resistance in two distinct *F*_2_ populations, demonstrating that these will be useful for watermelon breeding for powdery mildew resistance.

### Putative gene sequence analysis

Multiple sequence alignment of three *LOX* candidate genes was performed using Clustal Omega (Online Resource 1, figure S1). The alignment revealed lack of conserved regions in the upstream and middle part of the genes. In contrast, the downstream region having PLN02337 lipoxygenase catalytic domain was highly conserved. Furthermore, gene sequences of five candidate genes from the resistant parent USVL531-MDR and the susceptible parent USVL677-PMS were compared (Online Resource 1, figure S2-S5). Pairwise alignments showed almost complete identity across all candidate genes. The ClCG02G015710 gene sequence displayed 4785/4797 identical positions (99%). The ClCG02G015720 gene (50S ribosomal protein-like) exhibited 3045/3050 identical positions (99%), and ClCG02G015750 aligned with 2837/2839 identical positions (99%). The longest alignment, ClCG02G015730, revealed 9873/9903 identical positions (99%) with only three minor gaps. These variations were evenly distributed to across the genes.

### AlphaFold2 prediction revealed a loop-region substitution in LOX (ClCG02G015730) gene

**Fig. 6:**
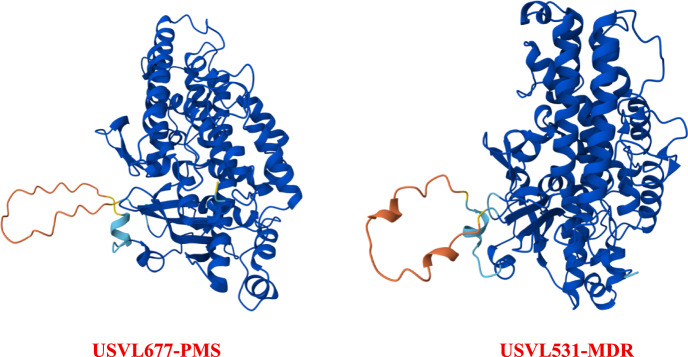
Predicted tertiary structures of the ClCG02G015730 protein from the resistant (USVL531-MDR) and susceptible (USVL677-PMS) watermelon genotypes. Protein 3D models were generated using AlphaFold2 (DeepMind; https://alphafoldserver.com). Both alleles exhibited nearly identical α-helical architectures with high per-residue confidence scores (pLDDT > 90), indicating well-defined and stable folding. The resistant allele (USVL531-MDR) differed from the susceptible allele (USVL677-PMS) by a single conservative Met ↔ Val substitution located within a flexible surface loop. This substitution does not alter the overall fold but may influence local flexibility or modify protein–protein interaction potential associated with powdery mildew resistance

To explore potential structural differences associated with powdery mildew resistance, translated sequences of ClCG02G015710, ClCG02G015720, and ClCG02G015730 from the resistant parent USVL531-MDR and susceptible parent USVL677-PMS were aligned. Both ClCG02G015710 and ClCG02G015720 showed complete amino acid identity between the two genotypes, whereas ClCG02G015730 exhibited a single conservative methionine (Met) to valine (Val) substitution to the resistant parent USVL531-MDR (Online Resource 1, figures S6–S8). Protein structure prediction for ClCG02G015730 was performed using the AlphaFold2 model (DeepMind; https://alphafoldserver.com). The predicted tertiary structures of both alleles displayed nearly identical α-helical architectures with well-defined and stable folding (pLDDT > 90) (Fig. 6). However, the two alleles differed by one Met ↔ Val substitution positioned within a flexible surface loop. This substitution, while not disrupting the overall fold, may influence local flexibility or protein–protein interactions, potentially contributing to differential responses to powdery mildew infection.

### Gene expression of candidate genes

Our laboratory has previously published a comparative transcriptomic study of USVL531-MDR and USVL677-PMS at 0, 24, and 96 h, and 8 days post-inoculation (Mandal et al. 2020). *LOX* genes were not focused and discussed in that manuscript. Expression levels of five candidate genes were observed in both genotypes before and after powdery mildew inoculation (Online Resource 2). Transcriptome analysis at 0 h, 24 h, and 96 h, and 8 days post-inoculation showed that ClCG02G015700 (Cla019900), ClCG02G015720 (Cla019898), and ClCG02G015750 (Cla019896) maintained stable expression levels after inoculation in both resistant and susceptible genotypes. Expression levels of all five candidate genes did not change significantly in USVL531-MDR after inoculation compared to before inoculation. In contrast, at 8 days post-inoculation, ClCG02G015730 (Cla019897) and ClCG02G015710 (Cla019899) expression was elevated 111-fold and 2.56-fold, respectively, in USVL677-PMS, while no differences in expression levels of these genes were observed at 24 and 96 h post-inoculation compared to pre-inoculation levels.

## Discussion

Powdery mildew is one of the most destructive fungal diseases of watermelon worldwide, causing significant losses annually (Keinath 2015). Development of disease-resistant cultivars is one of the best and preferred methods to manage powdery mildew. Molecular markers will be very helpful for transferring disease resistance genes from wild watermelon germplasm (*C. mucosospermus*) to cultivated watermelon species. In this study, QTL mapping and KASP marker development were conducted using an F_11_ RIL population derived from a cross between the resistant *C. mucosospermus* genotype USVL531-MDR and the susceptible *C. lanatus* genotype USVL677-PMS. This study mapped a major QTL on chromosome 2 for powdery mildew resistance and four KASP markers were developed and validated for MAS.

Transferring genes from *C. mucosospermus* to cultivated species is very challenging due to its several undesirable traits, such as white flesh, low sugar, bitter fruit taste, egusi-type seeds, and a hard rind (Kousik et al. 2023). USVL531-MDR, a watermelon line resistant to powdery mildew and Phytophthora fruit rot, was selected from PI 494531 within the USDA ARS germplasm collection. This line has demonstrated consistently high resistance to powdery mildew in comparison to commercial watermelon varieties such as Dixie Lee and Mickey Lee, based on evaluations conducted in both greenhouse and field environments (Kousik et al. 2023). USVL531-MDR was resistant to eleven *P. xanthii* isolate collected from different states and crops in the USA (Kousik and Ikerd 2014). Further testing with a wider range of *P. xanthii* isolates from different regions, both within and outside the USA, is required for better understanding the resistance conferred by USVL531-MDR. This will be helpful in determining the stability and effectiveness of resistance under diverse conditions against different *P. xanthii* races. The germplasm line USVL531-MDR is available to the public and private sectors for transferring powdery mildew resistance genes to cultivated watermelon. A genetic study is helpful for the efficient transfer of powdery mildew resistance from USVL531-MDR to susceptible cultivars. In this study, a RIL population derived from a cross between USVL531-MDR and USVL677-PMS genotypes was used for genetic analysis and QTL mapping. Our study of segregation data from the RIL and F_2_ populations revealed that powdery mildew resistance is controlled by a dominant gene. Previous studies have shown that powdery mildew resistance in watermelon is controlled by a single dominant gene (Tetteh et al. 2010; Deng et al. 2024; Kumar et al. 2025). In another study, powdery mildew resistance was governed by three complementary and partially dominant genes (Ben-Naim and Cohen 2015). Kim et al. (2015) demonstrated that the genetic inheritance of resistance is controlled by a single incompletely dominant gene (Kim et al. 2015).

In this study, a genomic region associated with powdery mildew resistance was identified on chromosome 2 (30,050,069–30,166,247), which contains six genes: five lipoxygenases (ClCG02G015690***,*** ClCG02G015700***,*** ClCG02G015710***,*** ClCG02G015730***,*** and ClCG02G015750**)** and one 50S ribosomal protein L27-like gene **(**ClCG02G015720**).** To refine the region, six KASP markers were developed based on these genes and tested for linkage with the resistance phenotype. Two (ClCG02G015690 and ClCG02G015700) of the genes did not exhibit co-segregation with the trait or with the remaining four markers. Consequently, the candidate interval was narrowed from 116,178 bp to a more precise 54,772 bp, enhancing resolution for downstream validation. *LOX* genes have previously been reported to play a potential role in powdery mildew resistance in plants (Hwang and Hwang 2010; Oh et al. 2014; Deng et al. 2024). The *ClLOX* gene reported by Deng et al. (2024) shares > 99% sequence similarity with ClCG02G015730, supporting that this locus represents the same functional gene. Furthermore, Kumar et al. (2025) mapped a 570 kb interval on chromosome 2 that include our fine-mapped 54.8 kb region. In contrast, the 50S ribosomal protein L27-like gene has not been linked to disease resistance in plants. However, homologs of this gene have demonstrated antimicrobial properties in other organisms, such as *Lactobacillus salivarius*, where it acts against *Streptococcus pyogenes*, *Streptococcus uberis*, and *Enterococcus faecium* (Pidutti et al. 2018), and in *Branchiostoma japonicum*, where it exhibits activity against *Staphylococcus aureus* and *Aeromonas hydrophila* (Chen et al. 2025). These observations suggest a potential but previously unrecognized role for this gene in defense mechanisms. The *LOX* gene family has been shown to positively regulate plant defense (Hwang and Hwang 2010). In the comparative transcriptome study, the *LOX* genes ClCG02G015710 and ClCG02G015730 were significantly upregulated following *P. xanthii* inoculation in the susceptible parent USVL677-PMS. No differential expression was detected for any candidate gene in the resistant parent USVL531-MDR, indicating that transcriptional regulation of these loci is not associated with resistance. The 50S ribosomal protein-like gene ClCG02G015720 also showed no differential expression between resistant and susceptible genotypes. These results suggest that resistance in USVL531-MDR may rely on post-transcriptional or post-translational regulation, with protein activity or structural variation playing a more critical role than transcript abundance.

The comparative analysis of *LOX* candidate genes between USVL531-MDR and USVL677-PMS revealed almost complete sequence identity, with only a few nucleotide differences evenly distributed across the genes. Importantly, the catalytic domain (PLN02337) was highly conserved, consistent with the strong selective pressure to maintain enzymatic activity. At the protein level, all putative genes were identical between the resistant and susceptible genotypes except for ClCG02G015730, which carried a single amino acid substitution. Structural modeling of ClCG02G015730 revealed a conservative Val ↔ Met substitution located within a flexible surface loop. Although this substitution did not alter the overall α-helical architecture, it may influence local conformational dynamics or protein–protein interactions, potentially affecting the activation or stability of downstream defense components. ClCG02G015730 encodes lipoxygenase, an enzyme involved in oxylipin-mediated defense signaling, suggesting that this subtle structural variation could modulate substrate binding or signal relay pathways associated with powdery mildew resistance. Flexible surface loops are increasingly recognized as critical determinants of protein function and interaction specificity in plants (Kozome et al. 2024; Corbella et al. 2023). Kozome et al. (2024) demonstrated that a loop region in a glycoside hydrolase family 19 (*GH19*) chitinase was essential for antifungal activity and pathogen degradation. Similarly, Corbella et al. (2023) highlighted the role of flexible loops in mediating loop–loop or loop–nonloop interactions during protein–protein or protein–ligand interactions. Therefore, the substitution in ClCG02G015730 may control functional differentiation between the resistant and susceptible alleles.

Our transcriptome results are contrary to previous studies. Hwang and Hwang (2010) demonstrated that overexpression of *Capsicum annuum* lipoxygenase 1** (***CaLOX1***)** in *Arabidopsis thaliana* enhanced resistance to *Pseudomonas syringae* pv. tomato**,** *Hyaloperonospora arabidopsidis***,** and *Alternaria brassicicola***,** while mutation of the endogenous *AtLOX1* gene reduced resistance. Overexpression of *CaLOX1* in pepper leaves also induced cell death and activated defense responses, whereas its silencing led to increased susceptibility to *Xanthomonas campestris* pv. *vesicatoria* (Xcv) and *Colletotrichum coccodes***.** These findings indicate that *LOX1* functions as a positive regulator of disease resistance in plants. *LOXs* are key enzymes in the oxylipin pathway, catalyzing the oxygenation of polyunsaturated fatty acids to produce hydroperoxides, which serve as precursors to signaling molecules such as jasmonic acid (JA)—a critical component of plant defense (Oh et al. 2014; Zhang et al. 2021; Singh et al. 2022; Deng et al. 2024). *LOXs* have also been shown to regulate pathogen-associated molecular pattern (PAMP)-triggered hypersensitive responses (HR), particularly during incompatible host–pathogen interactions (Naveed et al. 2020). A resistance-associated region on chromosome 2 was identified by Wu et al. (2019) through GWAS. Mandal et al. (2020) also found powdery mildew resistance on chromosome 2. More recently, Deng et al. (2024) identified a 0.93 Mb region on chromosome 2, suggesting a possible role for a *LOX* gene in powdery mildew resistance in watermelon using the XP-GWAS method. Kumar et al. (2025) also mapped 570 kb region to chromosome 2 using BSA-seq method. Our mapped region overlaps with Kumar et al. (2025) and Deng et al. (2024) studies. Transcriptome analyses in cucumber further support the involvement of *LOX* genes, with overexpression observed in response to *P. xanthii* inoculation (Oh et al. 2014; Zhang et al. 2021).

KASP is among the most widely adopted genotyping platforms in plant breeding for MAS, offering biallelic precision, scalability, and the ability to screen large populations efficiently at a relatively low cost. In this study, four KASP markers were developed to target a SNP that distinguishes the donor-resistant *C. mucosospermus* genotypes USVL531-MDR and PI 560003 from the recipient *C. lanatus* genotypes USVL677-PMS and ‘Sugar Baby’. CMPMR-3, CMPMR-4, CMPMR-5, and CMPMR-6 markers showed very strong linkage with powdery mildew phenotype in RIL and F_2_ populations. Previous studies have reported molecular markers associated with powdery mildew resistance in watermelon (Mandal et al. 2020; Deng et al. 2024). Mandal et al. (2020) developed a Cleaved Amplified Polymorphic Sequence (CAPS) based on transcriptomic data and upregulation of NBS-LRR gene in Chromosome 2 and validated it on an F_2_ population of USVL531-MDR × USVL677-PMS.This CAPS marker is located approximately 648 Kb from the locus identified in our current study. Deng et al. (2024) developed a derived CAPS (dCAPS) marker associated with resistance to *P. xanthii* race 2WF in *C. lanatus*. Deng et al. (2024) developed a derived cleaved amplified polymorphic sequence (dCAPS) marker linked to resistance against *P. xanthii* race 2WF in *C. lanatus*. Notably, this marker is located in the same genomic region identified in our study for resistance to *P. xanthii* race 1. Furthermore, marker data revealed 1.64% heterozygosity in the RIL population. Although *F*_11_ RILs are expected to be ~ 99.95% homozygous, a low level of residual heterozygosity at certain loci is expected. Overall, our results indicate that these markers can be used to facilitate MAS for powdery mildew-resistant genotypes in watermelon breeding programs.

A major QTL was mapped on chromosome 2 for powdery mildew using the RIL population (*F*_11_) derived from an interspecific cross. Four tightly linked KASP markers were developed from the SNP identified within the mapped region. These KASP markers were validated on the *F*_2_ populations. In addition, KASP markers were developed for one additional powdery mildew resistance sources from *C*. *mucosospermus*. These results indicate that powdery mildew resistances genes are located in the same region in both *C*. *mucosospermus*. The development of tightly linked KASP markers with powdery mildew resistance will be useful for developing commercial disease-resistant watermelon cultivars.

## Supplementary Information

Below is the link to the electronic supplementary material.

Supplementary file1 (DOCX 171 KB)

Supplementary file2 (XLSX 443 KB)

Supplementary file3 (CSV 3356 KB)
