# Supplementary material for: Fine mapping, introgression, and KASP marker development for powdery mildew resistance in watermelon using an interspecific RIL population (Citrullus mucosospermus × C. lanatus)
Source: Theor Appl Genet. 2025 Nov 8;138(12):299. doi: 10.1007/s00122-025-05079-4 (PMC12596284; doi:10.1007/s00122-025-05079-4)
Supplement: Supplementary file 1 — Supplementary file1 (DOCX 171 KB) [file 122_2025_5079_MOESM1_ESM.docx]

**Putative gene sequence comparison between USVL677-PMS (susceptible) and USVL531-MDR (resistant)**

ClCG02G015710 ------------------------------------------------------------ 0

ClCG02G015730 ATGTTGATTGGAAGCATTGTGGGGGCTATCTCTGGGTTGAAGCATGGAGGGAAGAAAATC 60

ClCG02G015750 ------------------------------------------------------------ 0

ClCG02G015710 ------------------------------------------------------------ 0

ClCG02G015730 CATGGAACTGTGGTTTTGATGAGAGACAATGTTTTGGACTTCAATGATTTTGGTTCCACT 120

ClCG02G015750 ------------------------------------------------------------ 0

ClCG02G015710 ------------------------------------------------------------ 0

ClCG02G015730 GTTCTGGATAATCTTCATGAGCTTTTAGGGGGTGGTGTTTCTCTTCGACTTGTTAGCGCT 180

ClCG02G015750 ------------------------------------------------------------ 0

ClCG02G015710 ------------------------------------------------------------ 0

ClCG02G015730 CATCATGGAGACCCTTGTGAGTTTTACTTTACTTCGTACTTATTTTATTTTGATTTTTGA 240

ClCG02G015750 ------------------------------------------------------------ 0

ClCG02G015710 ------------------------------------------------------------ 0

ClCG02G015730 ACTTTCGAATGAGGCTAGATCTATTGTTAAGTTATTTATATATAATTATAATTTTTTTTT 300

ClCG02G015750 ------------------------------------------------------------ 0

ClCG02G015710 ------------------------------------------------------------ 0

ClCG02G015730 TTTAGTTTAATAAAATGAGGATTCAAATTTTTAATGAAATAGCTCAATGGAAAAAAATGC 360

ClCG02G015750 ------------------------------------------------------------ 0

ClCG02G015710 ------------------------------------------------------------ 0

ClCG02G015730 ATGTTGAATTATGTATGCTAGAGGTTTGCAAAACTATTTGAGATTTTACTTATAAAATCT 420

ClCG02G015750 ------------------------------------------------------------ 0

ClCG02G015710 ------------------------------------------------------------ 0

ClCG02G015730 TGGAAAAGCTTTTTTCTTCTTTGAGTGAATGAATTTTTCATAAAATTTGTTTTAGTATTT 480

ClCG02G015750 ------------------------------------------------------------ 0

ClCG02G015710 ------------------------------------------------------------ 0

ClCG02G015730 TGCCCATGGAACTACTCTGTGTTTCCATATTTTTTTTTTTTTCTTTTTTTTTTTTTTTTT 540

ClCG02G015750 ------------------------------------------------------------ 0

ClCG02G015710 --------------------------------------------CCACTTAAAAAGTATA 16

ClCG02G015730 TTTTTTTTTTTTTTTTTCTTTTTTTTTTTNNNNNNNNNNNNNNNNNNNNNNNNNNNNNNN 600

ClCG02G015750 ------------------------------------------------------------ 0

ClCG02G015710 GAGAGAAAGAACTCGAAAAAAAGAGAGGAAATGGTAGAAGAGTGCCTAAAAATTGGGAAA 76

ClCG02G015730 NNNNNNNNNNNNNNNNNNNNNNNNNNNNNNNNNNNNNNNNNNNNNNNNNNNNNNNNNNNN 660

ClCG02G015750 ------------------------------------------------------------ 0

ClCG02G015710 ACATATACGACTATGATGTCTACAATGATATTAGTGATTTAGATTCTAATTCAACAAATA 136

ClCG02G015730 NNNNNNNNNNNNNNNNNNNNNNTTTTTTTTTTTGTCTCTTCTATTTTATTTTTATCATTT 720

ClCG02G015750 ------------------------------------------------------------ 0

ClCG02G015710 AACCCCCTATTCTTGGAGGATTAGTGCCTTATCCACGCA-------GAGGAAGAACTGGA 189

ClCG02G015730 ATTTTCTTTTTGGTGCCTATTCCATTTTTTTTATAAAAAAAATTAACATCGGTATCTAGA 780

ClCG02G015750 ------------------------------------------------------------ 0

ClCG02G015710 CGACCACGCTCAAAAAAAGGCAA----GT------------------------------- 214

ClCG02G015730 CTTCAAACTTTGCATAAAAAGAAATATTTGTCTTTTTCTGTTACTTTTTTTAAAAATATT 840

ClCG02G015750 ------------------------------------------------------------ 0

ClCG02G015710 -------------------------ACAATTTAGACTTTCATAAAGGCTTATAATT---- 245

ClCG02G015730 ATTCAACACATAAACACATATATGAAATAGGAGGACGTTCCAAGGTGTTGATAGATCTCT 900

ClCG02G015750 ------------------------------------------------------------ 0

ClCG02G015710 -----------------CTAGCAATTAAACTCCTTGTCTTT------------------- 269

ClCG02G015730 TAATCATCTATGTCATGATATCATTTATTTTGGTTGTGCTTTGGACCTCCAAATCTAATC 960

ClCG02G015750 ------------------------------------------------------------ 0

ClCG02G015710 ---------------------------------CATAAGCAAATCAATTTAGCCTCTTGA 296

ClCG02G015730 TCTTTTTTTAATTATGATGTTACCATAATTGTTCTTAATTGAAAAGCTTTCC--TCTAAT 1018

ClCG02G015750 ------------------------------------------------------------ 0

ClCG02G015710 TTAGAATTTTTTTTTT------TTAAATCGTTTATACATTAGTTC--------------- 335

ClCG02G015730 CTTGCATATTTCATCTTTCTTTTGTGATGAGGTTTCCTCTAGCTCTATTGTAAATTTTGA 1078

ClCG02G015750 ------------------------------------------------------------ 0

ClCG02G015710 ---------TAATCGTCCATTTCATAATTAATCCAACATTTGAAGAATTTTGCAC----- 381

ClCG02G015730 ACCTCTCTATTCTCATTCTTTTGATAACATATCGACTCAATTTGGGATATGATGAGGGTG 1138

ClCG02G015750 ------------------------------------------------------------ 0

ClCG02G015710 -----ATGTGTAAGAAT----------TGATGT---------------ATTGACTATTTG 411

ClCG02G015730 TCACGATGTGTCAAGCTAGTTAAGATATCCTGATACACCTAATATCCTGTTTACTAGTAT 1198

ClCG02G015750 ------------------------------------------------------------ 0

ClCG02G015710 CAATTGTAG----------ACCTTAATAAATTATTTATATTGATCAACTTGTGATGATTT 461

ClCG02G015730 CTTGTTCAGAAGAAAAAAAAACATAAATACATAAATGTCTTAACCATCATGGGTTGGTCT 1258

ClCG02G015750 ------------------------------------------------------------ 0

ClCG02G015710 AGAAGTTTAACTTAAACAATTATAAGTCTCACAACATATTTTTCAATAAAATCTAAAGAT 521

ClCG02G015730 AGTGGTAGTGGAAATATCTT-------------------------AAAAAGTCCAAAGAG 1293

ClCG02G015750 ------------------------------------------------------------ 0

ClCG02G015710 CAAAGGTGT---AAATTGATTTTATTATTTCTAAACCATTTTTATCTTTGAAAATTTTAA 578

ClCG02G015730 CGAAAGGGTTATAAATTTAATCTATGGTGGC---GACCTACTTAAGATTTAATATTCTAT 1350

ClCG02G015750 ------------------------------------------------------------ 0

ClCG02G015710 AAATACTTTTT--AACCATCTTTTA--AAAATT--TAACCAAACACTACTCATTTAAAGA 632

ClCG02G015730 GAGTTTTCTTGACACCCAAATGTTTTGTTTATTGATTATTAGATATT-CAAGTTTGAAGG 1409

ClCG02G015750 ------------------------------------------------------------ 0

ClCG02G015710 AAAAGAACACCCAAAACAATCATTATATAT-------------GTTTAAGTTGGTGGCAA 679

ClCG02G015730 AAAAAAAATAAAAAATAAAAGCTTAAATACATCAAATGGGCAAATTTTATTTTGA-TCTA 1468

ClCG02G015750 ------------------------------------------------------------ 0

ClCG02G015710 GGTTAAAAATGTCAGTATCAATTGTCTCAATTTTACAAAATATTGATAGAAATTATATTG 739

ClCG02G015730 GGGAATACTTTACATTTTTTTTTTCGATATTTATAAACTAAATTGCTAT--ATTATATTA 1526

ClCG02G015750 ------------------------------------------------------------ 0

ClCG02G015710 ATAAAATAATGACATCAATGAATATTTTTAAAATGATAAAAATAAATATTCAAATTTTAA 799

ClCG02G015730 --------TTT----------------------------------------TCATTTTTT 1538

ClCG02G015750 ------------------------------------------------------------ 0

ClCG02G015710 AATAACATTTTATACTTTTTAAACTATCTCAAATAAATAAATAAATATATATATATATAT 859

ClCG02G015730 ACTTTTTTTTTTTTTTTTTTACAATACGTGAAGTACGAAAATTAAACTGTTTTTCTTCTT 1598

ClCG02G015750 ------------------------------------------------------------ 0

ClCG02G015710 AAAATTTATATTATATTCACAATAATAACATTTTGTTTGCCTATATATATTTCATGAA-- 917

ClCG02G015730 A-AATTTTTATAAAAATTTCTTTCAATATATATTACTTGTCATTGAATTTTTCATAATTT 1657

ClCG02G015750 ------------------------------------------------------------ 0

ClCG02G015710 ------------------------------------------------------------ 917

ClCG02G015730 ATTTAAAATATCTTACATCATTAATGATTTCAACACGATATATATACAAACATTCAAATT 1717

ClCG02G015750 ------------------------------------------------------------ 0

ClCG02G015710 -----------TATTTAATTTTTTACTATGCATTGCAAATGTCGAC------------TT 954

ClCG02G015730 CACATAATACTCAATAAATTTTTTATTAAACACTCTACACATAAAGTAAACTACAATAAT 1777

ClCG02G015750 ------------------------------------------------------------ 0

ClCG02G015710 ATTGTTTATATCGATGTCAAAGCAACTCATAGACACATGTGAAAATGTTCGAAAATGTCT 1014

ClCG02G015730 TTAATTATTTTGGCTAATACAAAGACCAATACAAAAGTGTGAAAGTCTTTGGAGAGACCA 1837

ClCG02G015750 ------------------------------------------------------------ 0

ClCG02G015710 AACATTTTGAAGAAAACTGAATACTTAATTTGTTGTGAATTAACAGATGATAG-ATATGA 1073

ClCG02G015730 AGTTGTTTTTTTTTTTTTTTAATTTTATTTTTTT---ATTTTTAAAATGAATGAATTTAA 1894

ClCG02G015750 ------------------------------------------------------------ 0

ClCG02G015710 GACCAGATGTGCAATCAAAGATGTTTATGTTCCCAGTGATGAAAGATTTA---------- 1123

ClCG02G015730 AGGCGACTT-----TTAAATTTGTTACGATAACGATTGATTTAACATTTTCTTGAACTTC 1949

ClCG02G015750 ------------------------------------------------------------ 0

ClCG02G015710 ---------------GTGACTTGAAGAAATCAGATTTTG---ATATTCATGG-----ATT 1160

ClCG02G015730 TTTATATTCAATTTCAATTCTTTATCAAACAAAAAATTAGTCTTGTTCTTGCTAACCATT 2009

ClCG02G015750 ------------------------------------------------------------ 0

ClCG02G015710 AAGATCAGTGCTTCGAGACATTAAAGATAAACTTAAAG-------------------CTT 1201

ClCG02G015730 TAGGGCTCGGTTGGTATTTATTTTGCATCCATTTTTTTTTTTTTTTTTTTAACAAAGTAC 2069

ClCG02G015750 ------------------------------------------------------------ 0

ClCG02G015710 CATTAGGAAAATC---------TCCTA----------AAAGATTGGAGTCTCTTAAAGAT 1242

ClCG02G015730 TAGTGGGGATATCAATAGATGGACCTAGATATCTCAACTAGATTGACACATTTTTAGCAT 2129

ClCG02G015750 ------------------------------------------------------------ 0

ClCG02G015710 GTGTATGCAATCTATGAACCACGTTCCTTCTTTCGACGAGGGAAATTTCCAATGCCCCAG 1302

ClCG02G015730 CCTCATCATATACTTGCATTCAATTTTTTTAATATAAAATTTACGTTTTTTTTTTTCTTC 2189

ClCG02G015750 ------------------------------------------------------------ 0

ClCG02G015710 GTGATCGAAGGTATGTGTAACCCATTTTCTGACCTAAAACTTAAGTCCATGAAATTACGA 1362

ClCG02G015730 AAATTTTTTAATATGATTTTCATATTTTCTAACTAAACATTTGAATT-------CTAAGT 2242

ClCG02G015750 ------------------------------------------------------------ 0

ClCG02G015710 TAATGTTAAAAAGTACTTTTGGTATT-AATATGATTAGGGGCAGTTGCAAATATAAAAAT 1421

ClCG02G015730 CAAATTTAAAAAACAGTTTTTTTTTTTTTTTTAATTTTAA----------AAATTTTTTT 2292

ClCG02G015750 ------------------------------------------------------------ 0

ClCG02G015710 CATATCCAAATTGTTTGTAGATATAGCACAAC------ACGAACACAATAG--------C 1467

ClCG02G015730 AATTTACTATTTGATTGGTTTTTGAAAGCACTCCTAAAACATAGACATCAAAAGAAGAAA 2352

ClCG02G015750 ------------------------------------------------------------ 0

ClCG02G015710 AGATATGTCAAAACTTAAATTCTGCTTTTGAACCTATCAATGATAAACCATA-----TCA 1522

ClCG02G015730 AAATCAATAGATGCAAGTAGTATGTGTAGGCTCAATTTAAAAAAAAAATAAAAACAAAAT 2412

ClCG02G015750 ------------------------------------------------------------ 0

ClCG02G015710 CTAGTATGGTC---TATTAGTGATAGACCATTTTATTGTTAGCA---------------- 1563

ClCG02G015730 GTGAAATGGTTATCAAATGGGGCCTTAGTTTTTAGTTTTTTGCATTTTTTAAAATTAACT 2472

ClCG02G015750 ------------------------------------------------------------ 0

ClCG02G015710 -------------GTCTATTAGTGATCGATTTTGCTACATTTGTAATTCCTTAAAAACAT 1610

ClCG02G015730 TTGTTTCCTCATATTCCTTAAGTAAACAATTGAACCTTTTGTCCAATTCTAAAATAAAAA 2532

ClCG02G015750 ------------------------------------------------------------ 0

ClCG02G015710 TGTTATACACTTAATTATTATCGCTAAAAGTGTTATCCAATGCAATTGCCTATATGTTTA 1670

ClCG02G015730 CATTTTA-TTTTAATTTTTTTAGT-------TTTTAAAACTTGACT----TGAATGTTTA 2580

ClCG02G015750 ------------------------------------------------------------ 0

ClCG02G015710 TTGAAACAATGAACTCTAAATTTTATTTTGAAATTAAAGAAAGATTTCGTTAACCATTTA 1730

ClCG02G015730 AAACACTTTTAAAGTGTAGATAACAAAACAAAGAAATCAACAGAGGTTGACAAGTATTTT 2640

ClCG02G015750 ------------------------------------------------------------ 0

ClCG02G015710 GTCTAACTTTAGTATATAACAATACTAATTAATATCAAGTTTAGCAAAATGCGAAATTAG 1790

ClCG02G015730 TTTATTGTTTTTATTTTA--AAGACTGT-----GTAATGTGTAACATTAAGGATATT--T 2691

ClCG02G015750 ------------------------------------------------------------ 0

ClCG02G015710 TTGCAAAGAATGTAAGAAGTCTAAGAAACCCACTAGCCCATAAAAAAATAATAATAAATA 1850

ClCG02G015730 TATTCATGATTTCAAGTAGAGACGAACAACCTTGGAATATTTATTTATTTATTATTAATT 2751

ClCG02G015750 ------------------------------------------------------------ 0

ClCG02G015710 AAAAATAAAAAAACTCATCAAAATCATCAATGACTTTTAGGGTATGCTTGGGTTAACTTT 1910

ClCG02G015730 TTTAATTTTGGTATTCAACAAAACAGCTAAAGATTTTGAAGGGAAAGTGGGAGAGGCAGC 2811

ClCG02G015750 ------------------------------------------------------------ 0

ClCG02G015710 TCAAGTGTTTAAATTTGAATATAATTCATTTTGAAAAAAATTGAAGTTTTTGATAACCAC 1970

ClCG02G015730 ATACTTGGAGAATTGGGTTGGGAAT--------ACAATAATCCCAATTTTTGCTGGAGAA 2863

ClCG02G015750 ------------------------------------------------------------ 0

ClCG02G015710 TC-------AAAATAGTTTTTGAAACAC--ATTTAAAGTGTATTTTAAATAGCTTTTATC 2021

ClCG02G015730 ACAGCCTTCAGTATTACATTTGATTGGGATGAAGAAATTGGAGTTCCAGGTGCTTTCTTC 2923

ClCG02G015750 ------------------------------------------------------------ 0

ClCG02G015710 AAAAGAGTT-TAAATAAAAATGACTTTTTTGAAAAACATTTTTTTCCCTAGTCAATCCAA 2080

ClCG02G015730 ATTAGAAATGAACATTTTAGTGAATTCTTTCTCAAATCTCTCACTCTTGAGGATGTCCCT 2983

ClCG02G015750 ------------------------------------------------------------ 0

ClCG02G015710 A----TA-GACCCTTACTC-TATT-------TGGTGATTGATCTTTAACCATGATACTCT 2127

ClCG02G015730 GGCCATGGTAGACTTCATTTTGATTGCAATTCTTGGGTTTATCCTGCTGATAAATACAAA 3043

ClCG02G015750 ------------------------------------------------------------ 0

ClCG02G015710 ATCGGTCGTAAACTTCTAGATAATCGGCTATATATTTACAA---AATCTTAAAAACGCAT 2184

ClCG02G015730 AATGATCGTATATTTTTTGCCAATCAGGTAAAGTTTCATGATCAAGTCCTTATCCATCAT 3103

ClCG02G015750 ------------------------------------------------------------ 0

ClCG02G015710 TGCTACCGTACAATTTTTCTT-----------TT-----------ATTATTTACTTTTTT 2222

ClCG02G015730 TAAAACGTTACAAAAATGGTATTGCATGAATTTGAAAACCACGATACTTGTTATGAAGCT 3163

ClCG02G015750 ------------------------------------------------------------ 0

ClCG02G015710 GTAGTTTATAAACACATATGATCATCGTCAGTTTATTG---------------------- 2260

ClCG02G015730 ATCAATTATCATTATATAATAACCTCGTAATTTACTATTTCATGTCGAAGTAAGGTTTTA 3223

ClCG02G015750 ------------------------------------------------------------ 0

ClCG02G015710 --ATCTACATA----TATATACAACTCATTAAATCTTAGAAAATATTTATAAATTTTAAT 2314

ClCG02G015730 AAGACTAAGGCAATGTAAAATTGACATGTTTGATTTTGTAACTTTTTTTTTTTTTTTAAT 3283

ClCG02G015750 ------------------------------------------------------------ 0

ClCG02G015710 CTACAAACATTAAGACTCTGTTTGGTAACGATTTTGTTTTTAAAATT------------- 2361

ClCG02G015730 TT-GAGAAATGAAAACACAAGATATTTCTCACTAAGATAATAAAAATATATCTTAATTTT 3342

ClCG02G015750 ------------------------------------------------------------ 0

ClCG02G015710 ------------TTTCCTTGCGTTCTCATAAAATTCGATAGTATGATGTTCATATTTCTT 2409

ClCG02G015730 ACATCATTGTTGCATCCTTGAGCTTTAACACATTTAGCTAGAGG----GAAAGATTTGAT 3398

ClCG02G015750 ------------------------------------------------------------ 0

ClCG02G015710 CAAGAAACACTTGAATTCTTAATCAAATTCTAACAACAAAAACAAGTTTTCGAAAATAGT 2469

ClCG02G015730 TAAAAAAAATTCAA------TATAAATGTGTAAAA--AA---AAATTAATCTAAAAT-TT 3446

ClCG02G015750 ------------------------------------------------------------ 0

ClCG02G015710 TTTGAAATTTTGGCATTTTATAAACATTTTAAAATTAAAACTTTAAGTAAAAATATCTTA 2529

ClCG02G015730 TCAACACATAAGTTGGATAATGATCACACTAATATTAGAAATTAAATTATATATATTTTT 3506

ClCG02G015750 ------------------------------------------------------------ 0

ClCG02G015710 GAATTTTAATGTGTTATTTTAAAATATTCTATGGGTTAATAAATCAAGGTTATTGGTGTA 2589

ClCG02G015730 AGCTTTTTACTTTTTATTTAAGAATTATTAAAAGGAAA---AGAAAAGTTTATTGTGCTC 3563

ClCG02G015750 ------------------------------------------------------------ 0

ClCG02G015710 ATTAAACAGGTAATAAATCTGG-ATGGAGGACTGATGAAGAGTTCGCTAGAGAAATGTTG 2648

ClCG02G015730 ATCAACTATTGTAGTAATCTATGTTTAATCACCTAT--CAACTTAGCTATCTTGATGATT 3621

ClCG02G015750 ------------------------------------------------------------ 0

ClCG02G015710 GCAGGAGTAAATCCTATGGTCATTCGTCGTCTCCAAGTAAGGGTTTGTTTGGATTAACC- 2707

ClCG02G015730 TA-------AATATTATAAT-----ATGGTATAAGGCACCTGGTTTTGCATGATAATGAC 3669

ClCG02G015750 ------------------------------------------------------------ 0

ClCG02G015710 TAAAAAGTAATTTTTTTTTGAAAATTTATTTTCATTTAAATACTGTTGAT------AAGA 2761

ClCG02G015730 ACTTTAATATATGATTTCTAGATTTTTTTTTTTTTTTTAGTACAACAAGTGGGAGATGGA 3729

ClCG02G015750 ------------------------------------------------------------ 0

ClCG02G015710 ACTTTTTAAAATGAAAACACTAATAACTTTCAA------AATCCATTTTGAGTGAT---- 2811

ClCG02G015730 GGATTCGAACCTTCCACCTCGAGGATGGAACGTTATGCCAATTATTGCTGAGCTAAGCTC 3789

ClCG02G015750 ------------------------------------------------------------ 0

ClCG02G015710 ----TGTC-AAACCCTTTACTT--------CTTTTAAAATAACTTATTTTTTAAAGCCTA 2858

ClCG02G015730 GCTTTGGCATAATCCCTAGCTTAGCCTATATATTTGGGATAATTAATATTTTAAAATAAA 3849

ClCG02G015750 ------------------------------------------------------------ 0

ClCG02G015710 AATATAAAAAATGCCCTTGAACTATGCACTTTATGTCAAAAA----T-ATTTCTGAACTT 2913

ClCG02G015730 AGGA-CTTCCTTGGCCCTTGACTATGGAATTAATTAAGATATAAGAGCAATTTGCATATT 3908

ClCG02G015750 ------------------------------------------------------------ 0

ClCG02G015710 TCAAAACTACCCTTAAACATTAAAAAAGAAAACAAAAATTCTCTTGCCGTTAGTTTTGGA 2973

ClCG02G015730 TATAATCGAAGTGTACCCTTTAAAATAT-----TTTGCTTCACATTTTATTAGTTTTGAA 3963

ClCG02G015750 ------------------------------------------------------------ 0

ClCG02G015710 TGGAAACTGTTAAAGTTTTGTTTCAAAAATACCCCTAAACTTTTCAAAAGTTTAAAAAAT 3033

ClCG02G015730 GTACCACATCTAAAAAATAATTAAAAAAAAG-----TCAAAGGTCAAAAGTTGCTAACCT 4018

ClCG02G015750 ------------------------------------------------------------ 0

ClCG02G015710 ACCA-----------------------------TTAACTCAAAGAAGGTTAAAAAATACC 3064

ClCG02G015730 ACTAAACAAACCTGTATATTCAATTTCAAAACTATTGCAATGAGAAGATTAACCCATCT- 4077

ClCG02G015750 ------------------------------------------------------------ 0

ClCG02G015710 TTCTTTGTAAGTATATGAACAGAAATCGTTAATACCACATTGCAAAAATAACTTTAAAAT 3124

ClCG02G015730 ATTTTATAAAGAATGTGAGGTGAT-----CTCTCCATTTTTTCAAACGACTCTTAAACAT 4132

ClCG02G015750 ------------------------------------------------------------ 0

ClCG02G015710 TAAAAA------AAAAAGTTTATAATTGCTCCTAACATTAATATAGTGATCAATTTGTTT 3178

ClCG02G015730 TAAAGAAAAAAAATATAGTTGATTAAATATTCGACAATTCATGTTTTTATAGTTTA---- 4188

ClCG02G015750 ------------------------------------------------------------ 0

ClCG02G015710 AAAGTTTTCTTAAGTTCGAAAATAATGAAT--TTAAAAACAAATTATATAGATATCCTCA 3236

ClCG02G015730 AGAGTAGGAGTGGATTGGTATATATCTCACATATATTTCAAAAATTGTGAGTCATCCTCT 4248

ClCG02G015750 ------------------------------------------------------------ 0

ClCG02G015710 TTTTTTTTTTCAT-----ATAGATACTCTCATTTTTCTCTTCATTTTTCAATTCTAACTT 3291

ClCG02G015730 TCATTTTTTCAATGTGAGATAATTGACCTCAATCTCTTTGGAGTTGGTTATTTCTGAACT 4308

ClCG02G015750 ------------------------------------------------------------ 0

ClCG02G015710 ACACAAATTTTCTCGCCAACCAAAATTTTAAGTTAAAATATAAAATCCCATCAAATCTCA 3351

ClCG02G015730 TTTT--------------------TTTTTTTTTTAAAGAATCAAATGT-----CCATTTA 4343

ClCG02G015750 ------------------------------------------------------------ 0

ClCG02G015710 CAAAATTCTGAAATAAAAATTTTCTCT-TAAACATAACAAAAAGAAAAAAATGTTAGCAG 3410

ClCG02G015730 GGTAATTCATATGTTGTTTTGATACCATATTATATAAACATAGAGGATTAGTTCTCAAAA 4403

ClCG02G015750 ------------------------------------------------------------ 0

ClCG02G015710 TTGAGTATGTGTTAATAACTAAATACATTTTTTTATTTAA-----CTATTTATCGTTTTG 3465

ClCG02G015730 CCAATTGTGTGATACTCACCACAAACCATTGCTTTTTGAATGAGTGTATATATAATGTTT 4463

ClCG02G015750 ------------------------------------------------------------ 0

ClCG02G015710 GTATGTTTAAGGGAAGATTTTGATTTTAGAATTTTGTGAGATTTTATGTTATTTTATGCT 3525

ClCG02G015730 TGATCAACTGAGCTA-TGTTTGGGTTGACAATATCGATAAT------TTGAATACAGGCA 4516

ClCG02G015750 -------------------------------CGAATTGAAA------TTGTTTACAGACC 23

* * * * *

ClCG02G015710 TAACTTACAATTTTGGTTAGTGAGAAAATTA--------GTGTAAGCTGAGAATTGGAAA 3577

ClCG02G015730 TACCTTCCTAATGAAACACCGGAGCCACTTCGCAAATATAGGGCTGATGAACTATTGAAT 4576

ClCG02G015750 TATCTTCCAAGTGAAACGCCAGAGCCACTTCGCGAGTTCAGAGAGGACGAATTACGGAGC 83

** *** * * * *** * ** * ** **

ClCG02G015710 CTTAAGAGGAAAA-----------------C------------------AAAAGTATCTA 3602

ClCG02G015730 CTTAGAGGAAATGGAAAAGGAGAGAGAAAGGAATGGGATAGAATTTATGATTATGATGTA 4636

ClCG02G015750 TTGAGAGGAGACGGCAATGGCGAACTTCAAGAATGGGATAGGGTTTACGATTATGCTCTC 143

* * * * * * * *

ClCG02G015710 TATAAAAAAAATGAGGATATCTCTAT-------------------AATTTGTTTTAAAGT 3643

ClCG02G015730 TACAATGATATTGGTGACCCAGATAGTAATTTGGACCTTGGTCGTCCTATACTTGGAGGT 4696

ClCG02G015750 TACAACGACCTTGCCGATCCAGATAAAGGTCCACAATACGCTCGTCCTGTCCTCGGTGGC 203

** ** * ** ** ** * * * *

ClCG02G015710 TCATTCTTTTTTAACTTAAGAAAACTTCATACAAACCGATCACCATATTAATGTTATGAG 3703

ClCG02G015730 TCATCCAAATATCCGTACCCTCGTAGAGGAAGAACGGGAAGACCACCTTCCAAGAAAGGT 4756

ClCG02G015750 TCCACCAACTATCCTTACCCTAGGAGAGGAAGAACCGGACGACCCCCGACCAAATCAGGC 263

** * * * * * ** ** *** *

ClCG02G015710 CAATTTTAAACTTTTTGTTTTTTAATTTTTAAAGTTATTTTTG----TAACGAGGTATTA 3759

ClCG02G015730 AAATTATATATACATATATTTTTATTT--------------------------------- 4783

ClCG02G015750 AAGTTAATCGAAATCTCTTCCCCTTCATTTTCCGTTGATTTTGAATGAAAACTAGTTTCA 323

* ** *

ClCG02G015710 ACGGTTTCTG------TTCATATACTACGGTGAAGGTAAAAATGAATTTTTTTAGTTAAC 3813

ClCG02G015730 ------------------------------------------------------------ 4783

ClCG02G015750 TAGGTTCAATTTCTTTTTCAGATCCTGAAACTGAGAGCAGAATACCACTTATTAATAGCT 383

ClCG02G015710 GGTATTTTTGAAACTTTTGAAAAGTTCAAGGGTATTTTTGAAACAAACTTAAACGGCTTC 3873

ClCG02G015730 ------------------------------------------------------------ 4783

ClCG02G015750 TAAAC---ATTTACGTTCCAAGAGATGAGAGAT-TTGGTCACTTGAAATTGTCCGATTTT 439

ClCG02G015710 CATC--------------------------------TAAAAATAACGACAAGGGTATTTT 3901

ClCG02G015730 ------------------------------------------------------------ 4783

ClCG02G015750 CTTGCTTATGCGTTGAAGGCAGTTTCTGAGTTTATTAAACCTGGTCTCGAAGAGTACTTT 499

ClCG02G015710 CTTAACTC------------------TTTTTGAAAGTTTAAGGATATTTTTGAAAAAAAA 3943

ClCG02G015730 ------------------------------------------------------------ 4783

ClCG02G015750 GAAGGCACTCCGGGCGAATTCGACATCTTACAGGATGTTCTTGATCTTTATGAAGGAGGA 559

ClCG02G015710 TGCATAGTTCAAAGGCATTTTTTTT-TAATATAATTTA--ACCTTTTTTAAATTAAACAC 4000

ClCG02G015730 ------------------------------------------------------------ 4783

ClCG02G015750 TTCCCAGTGCCGGAAGGTTTGTTTGAGCTTATCAGCGAAAACATTGCTGCTCCTTTGCTT 619

ClCG02G015710 TCGAAAAGTATTTCAAATATACCCTAAATGTCAATTAATGTGTAATTTCTTTTGGAATTT 4060

ClCG02G015730 ------------------------------------------------------------ 4783

ClCG02G015750 AAGGAAATT-TTTAGAACTGATGGTGAAAGGCTCTTCAAA-----TTCCCTATGCCTCAA 673

ClCG02G015710 TTATTTTAAATTAAAGTTAATTATATC-GATTAATTAAGACTTCGTTTGATATATATATA 4119

ClCG02G015730 ------------------------------------------------------------ 4783

ClCG02G015750 GTGATTAAAGGTATATGAAATTGTAATCTCTGGTTACGTAATTTGGGTTAAATTTCTGAT 733

ClCG02G015710 TATTTTTTGGCATATTAATTATAGAGTTTCCCACCGACTAGCAACCTTAACCCTAGTGAC 4179

ClCG02G015730 ------------------------------------------------------------ 4783

ClCG02G015750 GAAATTTAATGATCTTTGTAACAGAGGATAGGTCTGCATGGAGGACTGATG----AAGAA 789

ClCG02G015710 TATGGTGATCAAAACAGCAAGATAACACCAAAACAG---ATTATGAATGGTATGGAGGGA 4236

ClCG02G015730 ------------------------------------------------------------ 4783

ClCG02G015750 TTTGGTA---GAGAAATGTTGGCCGGAGTCAACCCTGTAGTCATTCGTCGTCTCCAAGTA 846

ClCG02G015710 CTTACGGTAGA-------CCAGGTAATCCTTTTCTTTTCTTTTCCTAATTAAATAGACTT 4289

ClCG02G015730 ------------------------------------------------------------ 4783

ClCG02G015750 TTCTCGGCAATCTATTAACAACCTCATTATTCGAATTTATAATTCTCTCTGAATCCATTC 906

ClCG02G015710 AGTACTTTCGATTTTTTTTTTTTTTTCATTCAAATCTGTCCCTAATCTTTTTAATATCAT 4349

ClCG02G015730 --------------TTTTTTT-T------------------------------------- 4791

ClCG02G015750 GAAATTTGAAATT-TCTTTTC-T--AGAGAAAGATATAAAATGTGGGGTGTGAGGATGAA 962

* **** *

ClCG02G015710 T------TTTTATCGTT-AACTCGAGCATAATCACTATTTCAAAAGTTGATGATTTAATT 4402

ClCG02G015730 -----------AAAGAAAAGCTTTGATA-ATATCTAACCTCAACA-TCTATCTTTGTGTT 4838

ClCG02G015750 TTCAAAATTTTATATGAAAATTTTGGGATTAAGGAAATCTGAAAC-GGTATATATAT--- 1018

* * * * * * ** ** *

ClCG02G015710 CCTTTCATCCTCGTTATCGTTAACTGTTTGCATAATTGGACATTTTTCTATGATAATAAA 4462

ClCG02G015730 ----CACTCAAAAATATCTATATTTGTGTGGTCTAGACAATA---GTTAAATGTAGTAAA 4891

ClCG02G015750 ---------ATATATATATATATTACATTAAATTATACAACA---TACCCCTAAACTCAA 1066

*** ** * * * * * * **

ClCG02G015710 CAATTTTGAGAAGTTAAGTGGCTAAGTGC---------------------AAAGTCATGT 4501

ClCG02G015730 AATTTTTAAATCACAACTATATACATTCCTAAATCATTAATAATTACTACTAAGTTTAAT 4951

ClCG02G015750 AAAT------------CACTTTAGACTTCAAAAAATTTCAAAAATACTGTAGAACTTTCA 1114

* * * * * *

ClCG02G015710 AAATGAGTT-------------------TATTGTTTGGAAAGTAACAAAAATAGATTTCA 4542

ClCG02G015730 TAATCAATTAATTATTTCATCATCATATTAATGTTTGATTTTTTTTTAATTTTTTTCTTG 5011

ClCG02G015750 AAAACATTTAAAAAA-TACCCAAACTATTAGTGTTTTGTTT----CAAAAATACCTTTAA 1169

** * ** ** ***** ** * * *

ClCG02G015710 CTAGTAAAGCTCTATATATATAATA------------GAAAAA-------------TTAT 4577

ClCG02G015730 ATTTTTTCACTCTC--TATTTTTTAGT--ACAATAGAGATAGAGAAATTCAAACTACAAA 5067

ClCG02G015750 ATTTTCCGAAGTTCCAAAAATTCTATTATGCTTCATAAAAAAAAAACTTTAAAATTTCTC 1229

* * * * * ** * * *

ClCG02G015710 TTTATATTAAATGATAAAAA---TTTTGAAAGTATTTACAAATAATAGCAAAATATCACA 4634

ClCG02G015730 TCTTTTTGTCATTAACACATTCGT---ATGCC-------------AGTTGAACTATGTTC 5111

ClCG02G015750 TTGCTATTATATGAGCACAAACTGTTAATACTTGTTTAAAAAGATTCTTAAACTTTCAAA 1289

* * * ** * * * ** * *

ClCG02G015710 GTTTATCTGTGAATAGACCGCAATAGTTCACAATAGACTACTATTTGTATCTATCATGAC 4694

ClCG02G015730 ATTTTGATTTTTTTTTT-----TCAGTCTAGATGATGATAATTGTGTATTGTTTTATATC 5166

ClCG02G015750 GGTTGCATTAATACTCTTAGTCTTACAAAAAAAAAAGAAAGTTTGAAAATGCTTCAGCCG 1349

** * * * * * * * * * *

ClCG02G015710 ACAG--GTAGACACAGATACTAGTCTATCGCTTTCTATCGCAGATAGAAAGTGAAATATT 4752

ClCG02G015730 ATGCATGTTGCCCATTTTTTTCCTATCTCAAATGATTTAGAAGTTGG--TGGCAAAATTT 5224

ClCG02G015750 TTAGTTAGTGATCAATTTAT------TTGAAGTTTTCTGGGATTCGA--TAAGAATAATT 1401

* * * * * * * * ** **

ClCG02G015710 TTATATTTGTAAATATTTTAGTTCATTTTCTTATATTTGAAAACAACTCTATCTAATGTC 4812

ClCG02G015730 TTA------------TATTACTTTTTTTAAATG--TTTTATTTT------TTT----ATT 5260

ClCG02G015750 TAACAATAAAATATATATTTTTTCATATAGATA--TTTCGATTT------TTCTCCCTTT 1453

* * * ** ** * * * *** * *

ClCG02G015710 ATTTTGTTAGTTAATTAATTTTAATATTGTGGGGGGTAATAAAAACTTCAGTAAAAA--- 4869

ClCG02G015730 TTTTTATTTTTACAATGGTTTATATTTTGATGGTGAAGGAAACATCTTAGGTTTTAGGAA 5320

ClCG02G015750 TGTTCAATTCTTTCACCCATTAAAATTTCATGTTAAAACACAAAATTTCAGAATCAA--- 1510

** * * ** * ** * * * ** * *

ClCG02G015710 ----AAATTCAAATAAAGAATGAAATCGAAACTTTTAAAATAATTTTAAAATAATTCATA 4925

ClCG02G015730 GATTAAATGTTAATAGAAATATCAATAAAAAGTTAATA--------GAAAAAACTAAACA 5372

ClCG02G015750 AATCTTATCTTACCATAACACTAAATAGCCAGATACAA--------AAAGTTCATATTTG 1562

** * * * *** * * * ** *

ClCG02G015710 TGTATCAAATTAAAAGAGTATACACCCTAACATTAAGAATAAAATAAATAAACGATAAAA 4985

ClCG02G015730 TATATTAATATCATAAGCATTTCACCTTCAAA-ATGGCTTAAAATTAA-----------T 5420

ClCG02G015750 GCTATTAATACACACAACTACTAATGTACA--------ATCATGTTAA-----------A 1603

*** ** * * * * * **

ClCG02G015710 AATGGTTAAATTACAAATTTAATTTT---------CTATAGTTTAG-----------ACG 5025

ClCG02G015730 CAAGGTTAAATTGCAAATTCAATCAGTTTGTATCTCTTTAGTCCTTAAATTTC----AAA 5476

ClCG02G015750 ACATTTAAAGTCCGTGCTTCTATTATTATTTTTT-TTTTGCTTTTTATAATCTATATACC 1662

* ** * ** ** * * * *

ClCG02G015710 TAGGTTTAAACGGCACCTATAGTTTTAAAAGTATCAAAATTCTTATAATTTTAGTTTAGT 5085

ClCG02G015730 ATAATTGAAAGATGAGAATTTCATTATTCGATATAAAA---CTTATATTTATATTTAATA 5533

ClCG02G015750 TTAATTGAAGGGGGTGGTTTTTCATG---------------------------------- 1688

** ** * *

ClCG02G015710 TTCAATTTGATTCCTATA-ATTTTAAACATTTCAATTTGACCCGTTAGTTT--------- 5135

ClCG02G015730 ATTCAATTAATTTTCGAATTTGTCAAGGACCTCTATTAGACATAATATTGAAAGTTCAAT 5593

ClCG02G015750 ACAAACAAAGGTCAAACAATTAACAAGAAGGGGAATAAGAAAAGA-AGGGAGAAAAAAAT 1747

* * * * ** * ** ** *

ClCG02G015710 --CTTTTTCTCTGACATCTTAAATCTACCCCAAAA-----GATTAAGCTGATAAATAATG 5188

ClCG02G015730 GACGAAAGCACACACCTCACAATTTAAAGACTAAACTTGTAATTAATTAACCAAAAAATC 5653

ClCG02G015750 AGAGGAAATAGAGAA------GTGTTAGGCCA-----TATATTAAAGTAGGGGAGAGAAA 1796

* * * * ** * *

ClCG02G015710 ATAAATTTAATTATATCAATGTCATTA--------AGCGAAAATCAATAT---------- 5230

ClCG02G015730 GTAA-ATTAATA-TTACATTCACATTAG--TGATATTTGTAGACTTTTTTTTTTTTTCCG 5709

ClCG02G015750 AGAAATTGAAGA-GGAGGGTGATGGGGTGTAGATAAACAAGCATTTTTAACCTTTTTCAT 1855

** * ** * * *

ClCG02G015710 --TAATTGAAAAAAAAAGTAACCTCATAGGAATTAGAACGCAATACCTTTATGCTCTTAT 5288

ClCG02G015730 TTATATTTTACCACATATAACAGAAATATT----GTGATTAACTCCAAATGTAAAATTCT 5765

ClCG02G015750 TTTTATTATTTTAAATTTTAAGGTAAGGG-------TACT---------AATGCAACTTT 1899

*** * * * * * * * *

ClCG02G015710 TGGCCGGGTGAAAC---CAATAATATAA--TAAAAAAGATTA-AAGCTAATAAAT---TA 5339

ClCG02G015730 TGTATGTATGTAGATATAATTAAACTTGTTTAAAAAACATATAAGACTTTTTTATCGAGA 5825

ClCG02G015750 TGAAAGTTTAGAGATATTTTTGCAATG---------AGGTATGAATAGTTTTTGTTCATA 1950

** * * * * * * * * * * *

ClCG02G015710 GAATAAATTTAATTGTATCAATACTTTAGCCATCTTCT--TGTCATAATACAAATGTTAT 5397

ClCG02G015730 GGTCCGATGTTCAGGTAT-----ATTTATGGTTTTTTTTTTGTTTT---------TTT-- 5869

ClCG02G015750 TATTAACGGTAAAGGTTTTTATTTTTTGAGCTTTTCTTTAAGTTTTTTTTTTAATTTT-- 2008

* ** * *** * * * ** * **

ClCG02G015710 ATCTAAGGCAACACTTTTATTGTAGGAGGCTAAAAATAAAGTTTAATAAAAATCC----- 5452

ClCG02G015730 -TTTAATTTTTACT-----TATTACCATC-TAGTAGTCG---TGATTTGAAATTCATCAA 5919

ClCG02G015750 -TTTAATTTTTAAT-----TTTTTAATTTTGAAAGGTCAAGGGTATTTTCGAAACAAAAC 2062

* *** * * * * * * * *

ClCG02G015710 ---------TTGAATCGAAAAAAAAAAATAAATAAA------------TAAAGTGTTGTT 5491

ClCG02G015730 TCTT-----TCAAATTG-------ATAA----TATATATCTTAACCAATTGAGTTAGGGA 5963

ClCG02G015750 ACTAACGGTTTCCATCCAAAATTAACGGTAAGGATATTTTTGAAACATTTGAAAGTTTAA 2122

* ** * * * * *

ClCG02G015710 GTCAAATATAAAAAAATAAACCAAGATATTTATAAATAGAACAAAATTTTATAGTCTATC 5551

ClCG02G015730 GGAAAGTTGGCAATGATGAAACAACGTTTTAATATT--AAAGGTATGTTGAACTTCTATT 6021

ClCG02G015750 GGGTA--------TTTTTCACAAACGTTTTTTTTTT--TTTTTTTTTTTTTTTTTTTTTT 2172

* * * * ** * ** * ** * * *

ClCG02G015710 ATGGTCTAG-----ATAGAC-----CGTAATATTTTGCTATTATTTATAAATATTTTAAA 5601

ClCG02G015730 GTGATTTGAACATGCAGATCCCAAAAGTGAAAGTAGATTACCAGGTTCAAGTAAACCAAC 6081

ClCG02G015750 TTTTTTTTTTTTTTTTTTTNNNNNNNNNNNNNNNNNNNNNNNNNNNNNNNNNNNNNNNNN 2232

* * *

ClCG02G015710 CAATTTTGTCATTT--------AAAATAATTTTTCACAAATAAAAGTTTAACAACCAAAC 5653

ClCG02G015730 TGATATTTACGTTCCAAGAGATGAAAGATTTGGTCACTTGAAGATG--------TCAGAC 6133

ClCG02G015750 NNNNNNNNNNNNNNNNNNNNNNNNNNNNNNNNNNNNNN-NNNNNNN--------NNNNNN 2283

ClCG02G015710 TATATTTCTGAATATTTGTCTTTGATCTCCGAATC----AAAGGGTTTTTTTTTTTTTTT 5709

ClCG02G015730 TTTCTTGCTTATGGCCTAAAATCAATTTCAAGATCAATCAAACCAAAATTGGATGATCTA 6193

ClCG02G015750 N-NN-NNNN-NNNNNNNNNNNNNNN--NNNNNNNNNNNNNNNNNNNNNNNNNNNNNNNNN 2338

ClCG02G015710 TTAATTTTAATTTTCGCTAACAATGATGTCTTTACTGTTGCTTTGAAT-TGATTTGTTTT 5768

ClCG02G015730 TTTGATAGCACCCCAGGGGAATTTGATGACTTTAATGATGTCTTTGACCTCTTTGAAAAA 6253

ClCG02G015750 NNNNNNNNNNNNNNNNNNNNNNNNNNNNNNNNNNNNNNNNNNNNNNNNNNNNNNNNNNNN 2398

ClCG02G015710 GGTATGTTTCTCACATACCCCACTTAATAAAGTATTCCTTATAAAAGTGGAGAACCATAG 5828

ClCG02G015730 GGTTTGCCTGTTCCAAGAACTTTGCTTGAGAGTATCACTGACAACATTCCAGCTCCATTG 6313

ClCG02G015750 NNNNNNNNNNNNNNNNNNNNNNNNNNNNNNNNNNNNNNNNNNNNNNNNNNNNNNNNNNNN 2458

ClCG02G015710 -----------------TATTGTAATTAAAATAGTTAGCATATTCATT---AGAATTTTA 5868

ClCG02G015730 CTTAAAGAAATCTTTAGAACTGATGGTGAAAGATTTCTTAGATTCCCAACTCCTCAGCTA 6373

ClCG02G015750 NNNNNNNNNNNNNNNNNNNNNNNNNNNNNNNNNNNNNNNNNNNNNNNNNNNNNNNNNNNN 2518

ClCG02G015710 ATTTCTTGAAATTTGATATGATATTTTGTTTTCAATTTTGTATATAGGCAATTGCAGAA- 5927

ClCG02G015730 ATCCATGGTATGTTTTTCTTTTTCTTGCTTTTCTTGTTTTAGTACAGAGTCCTCCCAAAG 6433

ClCG02G015750 NNNNNNNNNNNNNNNNNNNNNNNNNNNNNNNNNNNNNNNNNNNNNNNNNNNNNNNNNNNN 2578

ClCG02G015710 --AACAAGCTGTACATATTAGATCACCA----TGATTTA--ATAATTCCATATCTTAAAA 5979

ClCG02G015730 TAAAATAATTGCAACTAATATATATATATATATTTTTTATTATTATTAAATTTGTTTG-- 6491

ClCG02G015750 NNNNNNNNNNNNNNNNNNNNNNNNNNNNNNNNNNNNNNNNNNNNNNNNNNNNNNNNNN-- 2636

ClCG02G015710 GAATAAATACAACTTCCACAAAAACTTATGCTACAAGAACACTTCTCTTCCTAAAAAATG 6039

ClCG02G015730 -TA--TGGATAACTTACTGTGTGTTCTTTTTTTTTTTTTTTTTTTTTTTGGNNNNNNNNN 6548

ClCG02G015750 -NN--NNNNNNNNNNNNNNNNNNNNNNNNNNNNNNNNNNNNNNNNNNNNNNNNNNNNNNN 2693

ClCG02G015710 ATGGGACTTTGAAGCCTTTAGCAATTGAATTGAGCTTG--------------CCAC---- 6081

ClCG02G015730 NNNNNNNNNNNNNNNNNNNNNNNNNNNNNNNNNNNNNNNNNNNNNNNNNNNNNNNNNNNN 6608

ClCG02G015750 NNNNNNNNNNNNNNNNNNNNNNNNNNNNNNNNNNNNNNNNNNNNNNNNNNNNNNNNNNNN 2753

ClCG02G015710 --ACCCTCAAGGA---------TAC--CAATTTG-GAGCCATTAG-TAAAATATT-GTTG 6125

ClCG02G015730 NNNNNNNNNNNNNNNNNNNNNNNNNNNNNNNNNNNNNNNNNNNNNNNNNNNNNNNNNNNN 6668

ClCG02G015750 NNNNNNNNNNNNNNNNNNNNNNNNNNNNNNNNNNNNNNNNNNNNNNNNNNNNNNNNNNNN 2813

ClCG02G015710 CCAGA-TAAAGGAAAAATTGGGGAACCACTTTGGCAACTAGCTAAAGCTTATGTTGTTGT 6184

ClCG02G015730 NNNNNNNNNNNNNNNNNNNNNNNNNNNNNNNNNNNNNNNNNNNNNNNNNNNNNNNNNNNN 6728

ClCG02G015750 NNNNNNNNNNNNNNNNNNNNNNNNNNNNNNNNNNNNNNNNNNNNNNNNNNNNNNNNNNNN 2873

ClCG02G015710 CAATGACTCTGGTCACCATCAACTCATCAGCCACTGGTATAAGTTCTTTAATCTCTTTTA 6244

ClCG02G015730 NNNNNNNNNNNNNNNNNNNNNNNNNNNNNNNNNNNNNNNNNNNNNNNNNNNNNNNNNNNN 6788

ClCG02G015750 NNNNNNNNNNNNNNNNNNNNNNNNNNNNNNNNNNNNNNNNNNNNNNNNNNNNNNNNNNNN 2933

ClCG02G015710 ACTTTCATTTGATAACTATTTAGATTT-------------TTTATTTTTTATTTTTTATT 6291

ClCG02G015730 NNNNNNNNNNNNNNNNTTTTTTTTTTTTTTTTTTTTTTTTTTTTTTTTTTTTTTTTTTTT 6848

ClCG02G015750 NNNNNNNNNNNNNNNNNNNNNNNNNNNNNNNNNNNNNNNNNNNNNNNNNNNNNNNNNNNN 2993

ClCG02G015710 TTTTAAAATTTAACTTAT-AAATATTACTCTTCTCTTTGGATTTTTTTGTTATATCTATT 6350

ClCG02G015730 TTTGGTGATTTCAATAACTTGGAATAGAGGATAAGTTTGCATGGAGCACTGATGAAGAAT 6908

ClCG02G015750 NNNNNNNNNNNNNNNNNNNNNNNNNNNNNNNNNNNNNNNNNNNNNNNNNNNNNNNNNNNN 3053

ClCG02G015710 TTTTAAATTAGAACTTTTTTAATAGCTCTAAAATAACACATCTACATTTGTTAGACTATA 6410

ClCG02G015730 TT--GCAAGAGAAATG-----TTGG-CCGGAGTTAACCCCGTAGTTATTGCTCGTCTTCA 6960

ClCG02G015750 NN--NNNNNNNNNNNN-----NNNN-NNNNNNNNNNNNNNNNNNNNNNNNNNNNNNNNNN 3105

ClCG02G015710 TTAAATCAATTTTG---ATCATAAACTGGTTAATGTATCAATTTTATCAAATTTGGCCCT 6467

ClCG02G015730 AGTAATTTTTTTTTATAAAAATAATTTGGTTAAAATACAATTTTAGTC------------ 7008

ClCG02G015750 NNNNNNNNNNN-NNNNNNNNNNNNNNNNNNNNNNNNNNNNNNNNNNNN------------ 3152

ClCG02G015710 GAACTCTTTATTTTATCAAATTTTACTCTAAACATAAATAAGTGTGGTCATTATTTTTAC 6527

ClCG02G015730 ----TATGTATT---------TTCAATAAATCTTAAATTTAGTCTCTACTACTAGTTTAT 7055

ClCG02G015750 ----NNNNNNN-----------NNNNNNNNNNNNNNNNNNNNNNNNNNNNNNNNNNNNNN 3197

ClCG02G015710 CCACAATTCAAATTTCACTAATTTTGCCACTTATTTTAATTTAAAAAAAGTAAAAATCTT 6587

ClCG02G015730 GGTTGATTCCTAACTTACTTTTAATCTATGAGTCTTTCCACTAAAAAATTAAAAAATAAA 7115

ClCG02G015750 NNNNNNNNNNNNNNNNNNN---NNNNNNNNNNNNNNNNNNNNNNNNNNNNNNNNNNNNNN 3254

ClCG02G015710 CGTAGGCTTGTTAATTTCAATAAAATAAAGTT-TTGCAATAAACT---ATATATGTTCG- 6642

ClCG02G015730 ACTAGTATATTTGTATTTTCTTGCATGAAATTATTATTATTATTATTATTATTTAATCAT 7175

ClCG02G015750 NNNNNNNNNNNNNNNNNNNNNNNNNNNNNNNNNNNN--N--------N--NNNNNNNNNN 3302

ClCG02G015710 -------------ACTAATTATACTAATTAACCATTGAATAACATTTTCAAAACTCATAC 6689

ClCG02G015730 TTTTTAAAAAAAGGCGCCTTTTGAAGGACTAAAATTTAAGATTACTTATTGAAAGTATAG 7235

ClCG02G015750 NNNNNNNNNNNNNNNNNNNNNNNNNNNNNNNNNNNNNNNNNNNNNNNNNNNNNNNNNNNN 3362

ClCG02G015710 AACTTTAACAAAAAAAAAAAAAAAAAAAAACTTTAAGAGCCC---TTTATTTTGGCAGGC 6746

ClCG02G015730 AAATCTCATAATAGAAGGATCAAAATCGTATTTTAACCTAATATGTTTTTTTTTTTAATT 7295

ClCG02G015750 NNNNNNNNNNNNNNNNNNNNNNNNNNNNNNNNNNNNNNNNNNNNNN----------NNNN 3412

ClCG02G015710 CAACCTAAATTTCTAAG---AAGTGGATA-------TTTTGATTACCGCGCTTATTTAGC 6796

ClCG02G015730 GTAGTTTATTTACAAATATTAAGCTTATATCATGTTTTTTAATTACAGGAAT---TTCCT 7352

ClCG02G015750 NNNNNNNNNNNNNNNNNNNNNNNNNNNNNNNNNNN----NNNNNNNNNNNNN---NNNNN 3465

ClCG02G015710 GGTTATTGT-AAAATGCTCTGATATCCTATGATGCTCTTATAGAGAACATCTTGAGAGTT 6855

ClCG02G015730 CCTACAAGCAAACTGGATCCTAACATTTATGGTGATCAAACAAG---------CAAAATA 7403

ClCG02G015750 NNNNNNNNNNNNNNNNNNNNNNNNNNNNNNNNNNNNNNNNNNNN---------NNNNNNN 3516

ClCG02G015710 TTGGGTGTTCTCCTGAAACATGGGTTTTTTAGATTCCCGAGTTGAGGGCATTCTCATTTT 6915

ClCG02G015730 ACTGAAGAACACATAAAAGATGGCTTAGATGG----GCTAACTGTGGATGAGGTAATTAA 7459

ClCG02G015750 NNNNNNNNNNNNNNNNNNNNNNNNNNNNNNNN----NNNNNNNNNNNNNNNNNNNNNNNN 3572

ClCG02G015710 ACTTT-----------TTTGTCTTTTTTTCCTTTTATGTATATATATATAGCCTTTTTTT 6964

ClCG02G015730 ACATTAAACTAAGAATTTTAGAGTTGATTGCTTACTTTTTTGAAACTTTTCTTTAAATAA 7519

ClCG02G015750 NNNNNNNNNNNNNNNN--NNNNNNNNNNNNNNNNNN---NNNNNNNNNNNN---NNNNNN 3624

ClCG02G015710 TTTTTTTTTTTGTTATATCGTAGTTTTTATTACTCTTTAGCAAGATAATCAAATTTAAAT 7024

ClCG02G015730 AATTTGTTTTTGATAGGCAGTTGAGAAGAACAAGCTATACATATTGAATCACCATGAT-- 7577

ClCG02G015750 NN---NNNNNNNNNNNNNNNNNNNNNNNNNNNNNNNNNNNNNNNNNNNNNNNNNNNNN-- 3679

ClCG02G015710 TTAAATTAATATTTATTTTATAAAAATAT--TAAATTATGATATATTATTTTAGTGAGGA 7082

ClCG02G015730 -TCATTGATTCCATATCTTAGAAGAATAAATACAACTCCCACAAAGACTTATGCTACAAG 7636

ClCG02G015750 -NNNNNNNNNNNNNNNNNNNNNNNNNNNNNNNNNNNNNNNNNNNNNNNNNNNNNNNNNNN 3738

ClCG02G015710 -----AAATAACATATAAAAATGATATAAATTGTATTAATTTAAAAAGAAA-TTTAAATT 7136

ClCG02G015730 AACAGTTTTGTTCTTGAAAAATGATGGGACTTTAAAGCCTTTGGCAATTGAATTAAGCTT 7696

ClCG02G015750 NNNNNNN--N-NNNNNNNNNNNNNNNNNNNNNNNNNNNNNNNNNNNNNNNNN-NNNNNNN 3794

ClCG02G015710 GGTAAAAGTAAATTATGTTATATAAGTTCAAATTAATTTAAATTAATTATAAATTAATAA 7196

ClCG02G015730 ACCACAT---------CCTCA-AGGGGACAAATTTGGGGTCATTAGTAGAGTAATTTTGC 7746

ClCG02G015750 NNNNNNN---------NNNNN-NNNNNNNNNNNNNNNNNNNNNNNNNNNNNNNNNNNNNN 3844

ClCG02G015710 GTAGTTGTAAGGGCATTCTTGGAACATTTTGTAAATTTAAGACATGCCCAAGTAGAAATC 7256

ClCG02G015730 CTGCTAAGACAGGAGTTGATGGCACAATTTGGCAGCTAGCTAAA-------GCTTATGTT 7799

ClCG02G015750 NNNNNNNNNNNNNNNNNNNNNNNNNNNNNNNNNNNNNNNNNNNN-------NNNNNNNN- 3896

ClCG02G015710 TTTCAAAACAAATACATTTGTCAAAATATTTTTAGATATTTACAAAAATATTCCTACAA- 7315

ClCG02G015730 ACTGTTAATGATACTGGCTATCATCAACTTATTAGCCATTGGTAAAATTTTCTCTTACCC 7859

ClCG02G015750 NNNNNNNNNNNNNNNNNNNNNNNNNNNNNNNNNNNNNNNNNNNNNNNN-NNNNNNNNNNN 3955

ClCG02G015710 --AACAAACATGATTATCTAAAGATGTTGAAT--A------------------------- 7346

ClCG02G015730 TAAACTTTAATTTTAGTCTCCAGATTTGGTTTAAGTTGCATTGGGGTCCTTGAATTTTAA 7919

ClCG02G015750 NNNNNNNNNN--NNNNNNNNNNNNNNNNNNNNNNNNNNNNNNNNNNNNNNNNNNNNNNNN 4013

ClCG02G015710 TCTCTATAATTCTGTATATTTAT-------ATT-------------TTCGAGCATCTAAA 7386

ClCG02G015730 ACTCAACAATTTTTTAGCTATGAGTTCTGGGTTTCGTAGCATATCTTTGGTCCCTCTATT 7979

ClCG02G015750 NNNNNNNNNNN--NNNNNNNNNNNNNNNNNNNNNNNNNNNNNNNNNNNNNNNNNNNNNNN 4071

ClCG02G015710 CATTCTGA-TTATTTACATTCTTAAAAAATAAACGACCCACTGATATCATTTTTCTTCAA 7445

ClCG02G015730 TTTTTTTTTTTTTTTTAATTTCTAACAACTGCAAGTTAATGTGACACCTATTTTTTTAAA 8039

ClCG02G015750 NNNNNNNNNNNNNNNNNNNNNNNNNNNNNNNNNNNNNNNNNNNNNNNNNNNNNNNNNNNN 4131

ClCG02G015710 GAATATTGTCGATTTATTAAATTATATCAATTTA--GAGTCAAAA-----TTGATTTTTT 7498

ClCG02G015730 -AATA--ATTTAATTACTAAATTATATATAATATTTTTTTCTTATTGTTTTTTCTTTCTT 8096

ClCG02G015750 -NNNN--NNNNNNNNNNNNNNTTTTT-----TTTTTTTTTTTTTTTTTTTTTTTTTTTTT 4183

** * * * ** *** **

ClCG02G015710 TCGTGGAATA------TTTTTAGAGATGGGGATTTTATAATATTTACTTTATTTACAGGC 7552

ClCG02G015730 TCCTCCTTTCTCCCCCTCTCTCAACCTCCTCCCTCTAACAAATAAAGTTTTGTTTGTCGA 8156

ClCG02G015750 TTTTTTTTTTTTTTAATTTAGCCTATCTGTGCGTGTGTAGAG-AGAGATATTGTTCTCCT 4242

* * * * * * * * * *

ClCG02G015710 TAAACACACATGCCGTAATTGAGCCATTTGTGATTGCAACACATAGACAAC--TCAGTGT 7610

ClCG02G015730 AGAAGGAAAATCTTGTTGTTTATAGTTGAGTTTTTCATAAAACCAAAGACATATATGGTT 8216

ClCG02G015750 GGAATTGAATATTCATACTTTGAATTGCAGGAGTTTCCACCAACAAGCAAA--------- 4293

** * * ** * ** * * *

ClCG02G015710 TGTTCATCCGATTCACAAGTTGCTTGTTCCTCACTTTCGATACACCATGAAGATCAATGC 7670

ClCG02G015730 AATATTATAGATTTAAAAAGAAGAAAAACAAATATTTAGATACATTATGAACTACATCCA 8276

ClCG02G015750 ---------------------------------------------------CTTGACCCT 4302

*

ClCG02G015710 TCTTGCAAGATCAACCCTCATTAATACTGATGGTATTATTGAGAAAACTCAATATCCTTC 7730

ClCG02G015730 TCTATTTTGCTCTCTCCAG----CCACCAACAGAAAAGAAAAAAAAAATAGTTTTCTTCG 8332

ClCG02G015750 GAAGTTTTTGGGGATCAAA----ATAGTAAGATAACTGAAGA------------------ 4340

* * * * *

ClCG02G015710 TAAGTATTCTATGGA--GATGTCTTCTTTTGCTTATCAAAATTGGGA------------- 7775

ClCG02G015730 TAGGTAAACTATAGTTGGATGACTCCGTGGACGTCAAAAAGATGGGTGACAGTCTAGGGT 8392

ClCG02G015750 ------ACACATAATACATAACTTGGATGGACTTACAGTAGAAGAGGTGAA--------- 4385

** * * * * * * *

ClCG02G015710 ----------------CCTTACTCAACAAGCACTCCCTGCTGACCTAATCAAGAGGTAAT 7819

ClCG02G015730 GTACCGCAAATATATTTTTTAAAAGAAGAACACAGGCTCGAGGTGAAACCAAAAATTAAA 8452

ClCG02G015750 ----------------TTATCATCATAGAGTGCATAATCGAAATAAAATGAAG------- 4422

* * * * ** **

ClCG02G015710 AAGTTTCTTGGATAACCATTATCGATTTTGTTTGATAACCTTTTTATTTTTATATTTAGT 7879

ClCG02G015730 AATATTT--TGATAA---------------TTAGTTTGATTATTTTTTATTAGTTTGATT 8495

ClCG02G015750 ----TTT--TAATCC---------------TTGGAAAAC-------------AAAAGATG 4448

** ** ** * *

ClCG02G015710 AATGATATG------AAGTCGATCTCTTAGTAT--TGCTCGTTCTTGGATATTTCAAATT 7931

ClCG02G015730 CTTGGTCTACCTTGATGGTTTATTTATTTATTTTTTTCTTTTTCCTTGTTGATTTACATT 8555

ClCG02G015750 TATGATTAACATAGCTGAT---------AATATTTCTGTG-TAATTTGGTGGTTTAG--- 4495

** * * * * * * * * * ** *

ClCG02G015710 AAATAATTTGCTATTAACTAGTCAATCCTTGATCATTGCATACTTTACATATTCTATAGA 7991

ClCG02G015730 TCTTCCTTAAACAAATATTGATTTGTCACTTTTTTTTTTCCCCTTATCAAAACCTAATTA 8615

ClCG02G015750 ---GCAATCAAGAAGAACAGGTTGTTCAT-ATTGG-ATCACCATGAT-------TCACTG 4543

* * * * ** * * *

ClCG02G015710 AAGT----ATATGTATAGCTCAATTGATTAAAACATATGTTTTCAAC------------- 8034

ClCG02G015730 TAATCACACCATTTATGAGTAAATTCACACACCTACATATCTTCAATAAAGTTGAATAAT 8675

ClCG02G015750 ATGCCATACCTTAGACGAATAAATACAACTTCCACA-AAGACTTATGCCAGCAGAACCGT 4602

* * * *** * * *

ClCG02G015710 --TAATTAGAAGTTAGA--AGTTTGAATATTTTCTCCTTTGTAACTTAAAATAAATCTTA 8090

ClCG02G015730 ATTTCTCCGAACTTATATTTGTTTACATTTTCTATAATTTATAGGTTGAATACT--CATG 8733

ClCG02G015750 ACTTTTCCTTAAAGA--------------------AAATG-------GGACTCT--GAAG 4633

* * * * * *

ClCG02G015710 CCAAAAATATTATAGTTTATATTAAAATGAAATAAGTTCAAACTTTTGACTTCTTGGCCA 8150

ClCG02G015730 CAACAATCGAACCATT-----TGTGATAGCAACCAACAGACAATTAAGTGTTCTTCATCC 8788

ClCG02G015750 CCACTGGCAATCGAAC-----TGAGCTTGCCAAATCCT--CAGGGAGACGAATTTGGAGC 4686

* * * * * * * **

ClCG02G015710 AATTGGCTAGTTGGAGGATT------TTTTAATCTCTAATAACCATATT-CTT-AACTTC 8202

ClCG02G015730 AATTCACAAGTTGCTTGTTCCTCATTTTCGAGACACAATGAACATTAATGCTTTAGCTAG 8848

ClCG02G015750 AGTTAGCAAGGTTTTCTTTCC---------------------------------AGCTGA 4713

* ** * ** * * * **

ClCG02G015710 ACCAAAGTTGG----------TTTAAAAAAAAAAACACCACATTAGTTTTTTTCTTTAAT 8252

ClCG02G015730 ACAATCTCTTATTAATGCTGATGGAATTATTGAAACAACTCATTAT------CCAGCTAA 8902

ClCG02G015750 ACAAGGGGTTGGTAGTTCAATTTGGCAACTTGCCAAAGCTTATGCA------GCCGTAAA 4767

** * * * * * * ** * *

ClCG02G015710 TTCATACCTCCCTCGTTACTTT--------TTGGTCTTGAGCTTTCACAAAAGTAACAAT 8304

ClCG02G015730 ATACTCCATGGAGCTGTCTTCTTTTGTTTACAAAACTTGGGTCTTCCCTCAACAAGCTCT 8962

ClCG02G015750 TGAC--------TCTG-------------------------GCTACCATCAACTCATCAG 4794

* * * **

ClCG02G015710 TTTGTCTCCGAATTTTCGTATGTAATG---ACTTAG----TCTCTTTACTTAAACTTGTT 8357

ClCG02G015730 CCCTGCTGATCTAATCAAAAGGTAATCATTACAAAATATTTAGACCCCTTTGATAATGAC 9022

ClCG02G015750 CCAT--TGGTATAACTCTTCTGTGACATGGACTAAAAATTTGTGTATAGTTCTAAATCAT 4852

* ** * ** * * ** *

ClCG02G015710 AAAATTTAGTGCTTAAACTTTAATATATATAACAATTTAATAGGCCCTATATACTTTCAA 8417

ClCG02G015730 ATACATATATATATATATAT----TTTTTTGAAAATTAAATCTATATGATTGGTTTCTTT 9078

ClCG02G015750 ATGGATTTTTGAATATAATTTCGGTTTCATGTAGGTTGAATACTCATGCTGT-------- 4904

* * * ** * * * * ** *** *

ClCG02G015710 TTTTTTAATTTTTTTTACAATTTTAGTTGGTGACTTAATTAATCTTTATGTTTTATATAC 8477

ClCG02G015730 ATTTTGGGTTCAAAATTCAAGTTGGGTTTTGAAAACTA-CAAAAAT-AGTTTTTAAAAAC 9136

ClCG02G015750 ----------AATTGAGCCATTTGTGATTGCAACGAACCGACAACTAAGTGTTCTTCATC 4954

* * ** * * * * * * ** *

ClCG02G015710 AAGTTTGAATCGTAATAAATTTGTCAATATACACGTGCA-----------ATAAATTTAA 8526

ClCG02G015730 TTGTTT---TGTTTTTAGAATTT-TGTTAAGAAATTCCCATGTTTGCTTATTAGTTTAAT 9192

ClCG02G015750 CAGTTTACAAGCTGCTCCATCCT-CACTTTCGAGACACCATGAATATAAACGCGT----- 5008

**** * * * * * *

ClCG02G015710 TTAATTATAACAATTGTTTTTATGATAGCTAGAGATTAAA-----TCATTGTAAATTGTA 8581

ClCG02G015730 TTTACTATAGTCCTCATCTTTGTTTAAGGGACATTTGTAAATATAAGAAAGTTTAAAACA 9252

ClCG02G015750 -TTGCTCGACAGATACTCATTAATGCAGGTGGCATTTTGGAAGCAACAGTGTTTCCATCC 5067

* * * * * ** ** * * **

ClCG02G015710 AAATACATAGACTAAAAGTTATAATTTATCAAAGTTGAGTGAGTAAATCCTTAGGTGGAA 8641

ClCG02G015730 AAATAGGTCGATTATATTATATATATTTTCAATTCT-ATAAAATTATTATTACTTTTTAA 9311

ClCG02G015750 AAATATGCCATGGAGATGTCAGCTGTCTTATA---T-AAGGACTGGAT---TTTTCATGA 5120

***** * * * * * * * * * * * *

ClCG02G015710 TAAATTGTTAAAAATGTGTCTTTGTTGGAGTATTAACTCATCGCCCCAAATTTTTGAGTT 8701

ClCG02G015730 ACAAACGCTAGAAGGAGGGC-TTG----AAC-----CTC----CGACCTTGTGGTTAACA 9357

ClCG02G015750 ACAAGCACTCCCTGCAGATC-TCATCAGGAG-----GTAAAAAATTCATAGTAGTAGTTG 5174

** * * * * * * *

ClCG02G015710 GACTGGAGATTTAACAT----------GATATCAATGTTCAAAACTATTGTA-------- 8743

ClCG02G015730 GCCACACGCTCTAACCAACTGAGCTATTCCAGCTTGCTTGAGCACTATCATAAAATGATA 9417

ClCG02G015750 GTATCGGGCACTAGTCTGTTTAAAGATTAATTTGTTTTCATACAGTATAGGAGC------ 5228

* * ** * * *** *

ClCG02G015710 --TCAACTAGTTGGATGTCATATATATGTTTAGGAAGTGATATATACACACAAAAA---- 8797

ClCG02G015730 ACATAACTTTATTATATATATATATATATATATCACACACTATTTTGTCTCTT-GTTTTT 9476

ClCG02G015750 --------CTGTTGTGTATA----GATTTTAAGTTAAACATATTCTGATACTTTACAAAC 5276

* * ** * * *** *

ClCG02G015710 -AAAAA-AAAAATTAATCCAACTTTCTCTTTGGTTTGATTTGTTTAGAGGAATTGCAATT 8855

ClCG02G015730 TCTCACTTCATTCATTAACATTCAAACCTTGGCTTAATTTGTTAAAGAGGAGTTGCAACA 9536

ClCG02G015750 TAGTCATTGACTCAT-TTAACTTGTTCAAAATCATTGTTTCGTTTAGAGGAATGGCAATT 5335

* * * ** * ****** * ****

ClCG02G015710 GAAGATCCAAGTGCCCCACATGGACTCCAATTACTCATAAAAGATTATCCATATGCTGTT 8915

ClCG02G015730 GAGGATTCAAGCTCTCCCCATGGACTTCAACTACTAATAGAAGATTATCCATATGCTGTT 9596

ClCG02G015750 GAGGATACAAATTCTCCACATGGACTTCGTCTTGTAATCGAGGACTATCCATATGCTGTC 5395

** *** *** * ** ******** * * * ** * ** **************

ClCG02G015710 GATGGACTTGACATTTGGACAGCCATCAAAACATGGGTACGAGAGTATTGTTCAATTTAC 8975

ClCG02G015730 GATGGTCTTGAGATTTGGTCAGCAATCAAAACATGGGTACAAGATTATTGCTCTTTCTAC 9656

ClCG02G015750 GATGGACTTGAGATTTGGTCAGCGATCAAGACATGGGTTACGGATTATTGTTCTTTCTAT 5455

***** ***** ****** **** ***** ******** ** ***** ** * **

ClCG02G015710 TACAAGAACGATGAAATGATTCGTAATGATCCAGAGCTCAAATCATGGTGGAATGAAGTT 9035

ClCG02G015730 TACAAAGATGATCAAACACTACATAATGATACAGAGCTCCAATCATGGTGGAAAGAGCTT 9716

ClCG02G015750 TACAAGACTGATGAAACGGTACAGAATGACTCGGAACTTCAGTATTGGTGGAAGGAACTT 5515

***** *** *** * * ***** * ** ** * * ******** ** **

ClCG02G015710 CGAGAAAGAGGCCATGAAGACAAGAAAGACGAACCATGGTGGCCAAAAATGCAAAGTATT 9095

ClCG02G015730 CGCGAGAAAGGCCATGCAGATAAGAAAGATGAACCATGGTGGCCAAAAATGCAATCCGTT 9776

ClCG02G015750 AGAGAGGAAGGTCATGGTGACAAGAAAGATGAATTATGGTGGCCTAAGATGCAGAATGTT 5575

* ** *** **** ** ******** *** ********* ** ***** **

ClCG02G015710 GAAGAGCTAATCAATAGTTGCACCATCATCATATGGATTTCTTCGGCTCTTCATGCTGCA 9155

ClCG02G015730 CAAGACCTAATACAAAGTTGTACAATCATTATATGGATCTCTTCAGCTCTTCATGCTGCA 9836

ClCG02G015750 GAAGAGTTAATACATTCATGTACTATCATCATATGGATTGCTTCAGCTCTTCATGCTGCA 5635

**** **** * ** ** ***** ******** **** ***************

ClCG02G015710 GTTAACTTTGGGCAATATCCTTACGGTGGTTTTCTTCCTAATCGCCCATCGACTAGCGTA 9215

ClCG02G015730 GTTAACTTTGGACAATATCCTTACGGTGGCTTTGCTCCCAATAGACCATCCACTAGTCGT 9896

ClCG02G015750 GTAAACTTTGGACAATACCCTTATGCAGGTTATCTTCCCAATCGACCAACTATAAGTCGA 5695

** ******** ***** ***** * ** * * *** *** * *** * * **

ClCG02G015710 CGATTCTTACCGGAAGAAGGCACATCTGAGTATCTAGAACTGCAGTCAAATACAGATAAA 9275

ClCG02G015730 CGGTTCCTACCAGAAAACGGTACTCCCGACTACAAAGAGCTCGAGACGAATCCCGAAAAG 9956

ClCG02G015750 AAATTCATGCCGGAAGAAGGCACTCCAGAGTATAAAGAACTCGAATCAGATCCTGAGAAA 5755

*** * ** *** * ** ** * ** ** *** ** * * ** * ** **

ClCG02G015710 GCTTTCTTGAAAACATTCACTTCCGAACTACAAGAAAATGATCTTCTAAATATCACTACA 9335

ClCG02G015730 GCGTTCTTGAGAACAATCACTTCACAATTACAAGCTCTTGT------GGGGGTGTCAGTT 10010

ClCG02G015750 GCTTTCTTAAGAACAATCACTGCACAACTTCAAACTCTTCT------TGGCGTCTCGTTG 5809

** ***** * **** ***** * ** * *** * * *

ClCG02G015710 ATTCAACTTTTATCCTCACATTCTCTTGATGAGTCCTATTTAGGGCAAAGAAGTGATCCG 9395

ClCG02G015730 ATTGAGATTTTGTCAAGGCATTCTTCAGATGAAGTGTATTTAGGGCAAAGAAGCGACCCT 10070

ClCG02G015750 ATAGAGATTTTGTCGAGGCATTCGTCTGATGAGGTCTATCTCGGCCAAAGAGACTCCCCC 5869

** * **** ** ***** ***** *** * ** ****** **

ClCG02G015710 AACTGGACTTTTGATAAGAATGCTTTAGATGCATTTGAGAATTTCAAAAGAAAGTTAGTT 9455

ClCG02G015730 GAATGGACTTTGGATAAGGAAGCTTTGGAAGCATTTGAGAAGTTTGGGAAAAAGTTGGCT 10130

ClCG02G015750 GAATGGACTACAGACAAAGGAGTGCTGGAAGCGTTTGAGAAGTTCGGGAAAAAGTTGGCC 5929

* ****** ** ** * * ** ** ******** ** * ****** *

ClCG02G015710 GAAATTGGGGAAATGATTGGGAAAAGAAACAAAGATGATATGTTGAAGAATCGAGTTGGA 9515

ClCG02G015730 GAAATTGAAGGGAAGATTGCTATGAGAAATAAAGATCCTCAATTGAAAAATAGAGTAGGA 10190

ClCG02G015750 GAGATCGAAGATGGAATCATGAAAAGAAATGAAGATTTGACACTTAGAAACAGAGTTGGA 5989

** ** * * ** * ***** ***** * * ** **** ***

ClCG02G015710 CGAGAGGTGAAGATGACATACACTTTGCTACTTCCCACTAGTCAACCTGGCATTACATGT 9575

ClCG02G015730 CC---TGTGGATATGCCATATACTTTGTTGTTTCCTACTAGTTCAGAAGGTCTCACAGGT 10247

ClCG02G015750 CC---GGTTCTGATGCCTTACACTTTGCTCTATCCATCCAGTGAAGAGGGGCTGACAGGG 6046

* ** *** * ** ****** * *** * *** * ** * *** *

ClCG02G015710 CGAGGAATTCCCAATAGCATTTCTATT--- 9602

ClCG02G015730 AGAGGAATTCCTAATAGCATTTCAATTTAA 10277

ClCG02G015750 AAAGGAATTCCCAACAGTGTCTCCATTTAA 6076

********* ** ** * ** ***

**Supplementary Figure S1.** Multiple sequence alignment of ClCG02G015710, ClCG02G015730, and ClCG02G015750 between resistant parent USVL531-MDR (531) and susceptible parent USVL677-PMS (677). Alignment was performed using Clustal Omega (<https://www.ebi.ac.uk/jdispatcher/msa/clustalo?stype=dna>).

677-710 CCACTTAAAAAGTATAGAGAGAAAGAACTCGAAAAAAAGAGAGGAAATGGTAGAAGAGTG

531-710 CCACTTAAAATGTATAGAGAGAAAGAACTCGAAAAAAAGAGAGGAGATGGTAGAGGAGTG

********** ********************************** ******** *****

677-710 CCTAAAAATTGGGAAAACATATACGACTATGATGTCTACAATGATATTAGTGATTTAGAT

531-710 CCTAAAAATTGGGAAAACATATACGACTATGATGTCTACAATGATATTAGTGATTTAGAT

************************************************************

677-710 TCTAATTCAACAAATAAACCCCCTATTCTTGGAGGATTAGTGCCTTATCCACGCAGAGGA

531-710 TCTAATTCAACAAATAAACCCCCTATTCTTGGAGGATTAGTGCCTTATCCACGCAGAGGA

************************************************************

677-710 AGAACTGGACGACCACGCTCAAAAAAAGGCAAGTACAATTTAGACTTTCATAAAGGCTTA

531-710 AGAACTGGACGACCACGCTTACAAAATGGCAAGTACAATTTAGACTTTCATAAAGGCTTA

******************* * **** *********************************

677-710 TAATTCTAGCAATTAAACTCCTTGTCTTTCATAAGCAAATCAATTTAGCCTCTTGATTAG

531-710 TAATTCTAGCAATTAAACTCCTTGTCTTTCATAAGCAAATCAATTTAGCCTCTTGATTAG

************************************************************

677-710 AATTTTTTTTTTTTAAATCGTTTATACATTAGTTCTAATCGTCCATTTCATAATTAATCC

531-710 AATTTTTTTTTTTTAAATCGTTTATACATTAGTTCTAATCGTCCATTTCATAATTAATCC

************************************************************

677-710 AACATTTGAAGAATTTTGCACATGTGTAAGAATTGATGTATTGACTATTTGCAATTGTAG

531-710 AACATTTGAAGAATTTTGCACATGTGTAAGAATTGATGTATTGACTATTTGCAATTGTAG

************************************************************

677-710 ACCTTAATAAATTATTTATATTGATCAACTTGTGATGATTTAGAAGTTTAACTTAAACAA

531-710 ACCTTAATAAATTATTTATATTGATCAACTTGTGATGATTTAGAAGTTTAACTTAAACAA

************************************************************

677-710 TTATAAGTCTCACAACATATTTTTCAATAAAATCTAAAGATCAAAGGTGTAAATTGATTT

531-710 TTATAAGTCTCACAACATATTTTTCAACAAAATCTAAAGATCAAAGGTGTAAATTGATTT

*************************** ********************************

677-710 TATTATTTCTAAACCATTTTTATCTTTGAAAATTTTAAAAATACTTTTTAACCATCTTTT

531-710 TATTATTTCTAAACCATTTTTATCTTTGAAAATTTTAAAAATACTTTTTAACCATCTTTT

************************************************************

677-710 AAAAATTTAACCAAACACTACTCATTTAAAGAAAAAGAACACCCAAAACAATCATTATAT

531-710 AAAAATTTAACCAAACACTACTCATTTAAAGAAAAAGAACACCCAAAACAATCATTATAT

************************************************************

677-710 ATGTTTAAGTTGGTGGCAAGGTTAAAAATGTCAGTATCAATTGTCTCAATTTTACAAAAT

531-710 ATGTTTAAGTTGGTGGCAAGGTTAAAAATGTCAGTATCAATTGTCTCAATTTTACAAAAT

************************************************************

677-710 ATTGATAGAAATTATATTGATAAAATAATGACATCAATGAATATTTTTAAAATGATAAAA

531-710 ATTGATAGAAATTATATTGATAAAATAATGACATCAATGAATATTTTTAAAATGATAAAA

************************************************************

677-710 ATAAATATTCAAATTTTAAAATAACATTTTATACTTTTTAAACTATCTCAAATAAATAAA

531-710 GTAAATATTCAAATTTTAAAATAACATTTTATACTTTTTAAACTATCTCAAATAAATAAA

***********************************************************

677-710 TAAATATATATATATATATAAAATTTATATTATATTCACAATAATAACATTTTGTTTGCC

531-710 TAAATATATATATATATATAAAATTTATATTATATTCACAATAGTAACATTTTGTTTGCC

******************************************* ****************

677-710 TATATATATTTCATGAATATTTAATTTTTTACTATGCATTGCAAATGTCGACTTATTGTT

531-710 TATATATATTTCATGAATATTTAATTTTTTACTATGCATTGCAAATGTCGACTTATTGTT

************************************************************

677-710 TATATCGATGTCAAAGCAACTCATAGACACATGTGAAAATGTTCGAAAATGTCTAACATT

531-710 TATATCGATGTCAAAGCAACTCATAGACACATGTGAAAATGTTCGAAAATGTCTAACATT

************************************************************

677-710 TTGAAGAAAACTGAATACTTAATTTGTTGTGAATTAACAGATGATAGATATGAGACCAGA

531-710 TTGAAGAAAACTGAATACTTAATTTGTTGTGAATTAACAGATGATAGATATGAGACCAGA

************************************************************

677-710 TGTGCAATCAAAGATGTTTATGTTCCCAGTGATGAAAGATTTAGTGACTTGAAGAAATCA

531-710 TGTGCAATCAAAGATGTTTATGTTCCCAGTGATGAAAGATTTAGTGACTTGAAGAAATCA

************************************************************

677-710 GATTTTGATATTCATGGATTAAGATCAGTGCTTCGAGACATTAAAGATAAACTTAAAGCT

531-710 GATTTTGATATTCATGGATTAAGATCAGTGCTTCGAGACATTAAAGATAAACTTAAAGCT

************************************************************

677-710 TCATTAGGAAAATCTCCTAAAAGATTGGAGTCTCTTAAAGATGTGTATGCAATCTATGAA

531-710 TCATTAGGAAAATCTCCTAAAAGATTGGAGTCTCTTAAAGATGTGTATGCAATCTATGAA

************************************************************

677-710 CCACGTTCCTTCTTTCGACGAGGGAAATTTCCAATGCCCCAGGTGATCGAAGGTATGTGT

531-710 CCACGTTCCTTCTTTCGACGAGGGAAATTTCCAATGCCCCAGGTGATCGAAGGTATGTGT

************************************************************

677-710 AACCCATTTTCTGACCTAAAACTTAAGTCCATGAAATTACGATAATGTTAAAAAGTACTT

531-710 AACCCATTTTCTGACCTAAAACTTAAGTCCATGAAATTACGATAATGTTAAAAAGTACTT

************************************************************

677-710 TTGGTATTAATATGATTAGGGGCAGTTGCAAATATAAAAATCATATCCAAATTGTTTGTA

531-710 TTGGTATTAATATGATTAGGGGCAGTTGCAAATATAAAAATCATATCCAAATTGTTTGTA

************************************************************

677-710 GATATAGCACAACACGAACACAATAGCAGATATGTCAAAACTTAAATTCTGCTTTTGAAC

531-710 GATATAGCACAACACGAACACAATAGCAGATATGTCAAAACTTAAATTCTGCTTTTGAAC

************************************************************

677-710 CTATCAATGATAAACCATATCACTAGTATGGTCTATTAGTGATAGACCATTTTATTGTTA

531-710 CTATCAATGATAAACCATATCACTAGTATGGTCTATTAGTGATAGACCATTTTATTGTTA

************************************************************

677-710 GCAGTCTATTAGTGATCGATTTTGCTACATTTGTAATTCCTTAAAAACATTGTTGTACAC

531-710 GCAGTCTATTAGTGATCGATTTTGCTACATTTGTAATTCCTTAAAAACATTGTTGTACAC

************************************************************

677-710 TTAATTATTATCGCTAAAAGTGTTATCCAATGCAATTGCCTATATGTTTATTGAAACAAT

531-710 TTAATTATTATCGCTAAAAGTGTTATCCAATGCAATTGCCTATATGTTTATTGAAACAAT

************************************************************

677-710 GAACTCTAAATTTTATTTTGAAATTAAAGAAAGATTTCGTTAACCATTTAGTCTAACTTT

531-710 GAACTCTAAATTTTATTTTGAAATTAAAGAAAGATTTCGTTAACCATTTAGTCTAACTTT

************************************************************

677-710 AGTATATAACAATACTAATTAATATCAAGTTTAGCAAAATGCGAAATTAGTTGCAAAGAA

531-710 AGTATATAACAATACTAATTAATATCAAGTTTAGCAAAATGCGAAATTAGTTGCAAAGAA

************************************************************

677-710 TGTAAGAAGTCTAAGAAACCCACTAGCCCATAAAAAAATAATAATAAATAAAAAATAAAA

531-710 TGTAAGAAGTCTAAGAAACCCACTAGCCCATAAAAAAATAATAATAAATAAAAAATAAAA

************************************************************

677-710 AAACTCATCAAAATCATCAATGACTTTTAGGGTATGCTTGGGTTAACTTTTCAAGTGTTT

531-710 AAACTCATCAAAATCATCAATGACTTTTAGGGTATGCTTGGGTTAACTTTTCAAGTGTTT

************************************************************

677-710 AAATTTGAATATAATTCATTTTGAAAAAAATTGAAGTTTTTGATAACCACTCAAAATAGT

531-710 AAATTTGAATATAATTCATTTTGAAAAAAATTGAAGTTTTTGATAACCACTCAAAATAGT

************************************************************

677-710 TTTTGAAACACATTTAAAGTGTATTTTAAATAGCTTTTATCAAAAGAGTTTAAATAAAAA

531-710 TTTTGAAACACATTTAAAGTGTATTTTAAATAGCTTTTATCAAAAGAGTTTAAATAAAAA

************************************************************

677-710 TGACTTTTTTGAAAAACATTTTTTTCCCTAGTCAATCCAAATAGACCCTTACTCTATTTG

531-710 TGACTTTTTTGAAAAACATTTTTTTCCCTAGTCAATCCAAATAGACCCTTACTCTATTTG

************************************************************

677-710 GTGATTGATCTTTAACCATGATACTCTATCGGTCGTAAACTTCTAGATAATCGGCTATAT

531-710 GTGATTGATCTTTAACCATGATACTCTATCGGTCGTAAACTTCTAGATAATCGGCTATAT

************************************************************

677-710 ATTTACAAAATCTTAAAAACGCATTGCTACCGTACAATTTTTCTTTTATTATTTACTTTT

531-710 ATTTACAAAATCTTAAAAACGCATTGCTACCGTACAATTTTTCTTTTATTATTTACTTTT

************************************************************

677-710 TTGTAGTTTATAAACACATATGATCATCGTCAGTTTATTGATCTACATATATATACAACT

531-710 TTGTAGTTTATAAACACATATGATCATCGTCAGTTTATTGATCTACATATATATACAACT

************************************************************

677-710 CATTAAATCTTAGAAAATATTTATAAATTTTAATCTACAAACATTAAGACTCTGTTTGGT

531-710 CATTAAATCTTAGAAAATATTTATAAATTTTAATCTACAAACATTAAGACTCTGTTTGGT

************************************************************

677-710 AACGATTTTGTTTTTAAAATTTTTCCTTGCGTTCTCATAAAATTCGATAGTATGATGTTC

531-710 AACGATTTTGTTTTTAAAATTTTTCCTTGCGTTCTCATAAAATTTGATAGTATGATGTTC

******************************************** ***************

677-710 ATATTTCTTCAAGAAACATTTGAATTCTTAGTCAAATTCTAACAACAAAAACAAGTTTTC

531-710 ATATTTCTTCAAGAAACACTTGAATTCTTAGTCAAATTCTAACAACAAAAACAAGTTTTC

****************** *****************************************

677-710 GAAAATAGTTTTGAAATTTTGGCATTTTATAAACATTTTAAAATTAAAACTTTAAGTAAA

531-710 GAAAATAGTTTTGAAATTTTGGCATTTTATAAACATTTTAAAATTAAAACTTTAAGTAAA

************************************************************

677-710 AATATCTTAGAATTTTAATGTGTTATTTTAAAATATTCTATGGGTTAATAAATCAAGGTT

531-710 AATATCTTAGAATTTTAATGTGTTATTTTAAAATATTCTATGGGTTAATAAATCAAGGTT

************************************************************

677-710 ATTGGTGTAATTAAACAGGTAATAAATCTGGATGGAGGACTGATGAAGAGTTCGCTAGAG

531-710 ATTGGTGTAATTAAACAGGTAATAAATCTGGATGGAGGACTGATGAAGAGTTCGCTAGAG

************************************************************

677-710 AAATGTTGGCAGGAGTAAATCCTATGGTCATTCCTCGTCTCCAAGTAAGGGTTTGTTTGG

531-710 AAATGTTGGCAGGAGTAAATCCTATGGTCATTCGTCGTCTCCAAGTAAGGGTTTGTTTGG

********************************* **************************

677-710 ATTAACCTAAAAAGTAATTTTTTTTTGAAAATTTATTTTCATTTAAATACTGTTGATAAG

531-710 ATTAACCTAAAAAGTAATTTTTTTTTGAAAATTTATTTTCATTTAAATACTGTTGATAAG

************************************************************

677-710 AACTTTTTAAAATGAAAACACTAATAACTTTCAAAATCCATTTTGAGTGATTGTCAAACC

531-710 AACTTTTTAAAATGAAAACACTAATAACTTTCAAAATCCATTTTGAGTGATTGTCAAACC

************************************************************

677-710 CTTTACTTCTTTTAAAATAACTTATTTTTTAAAGCCTAAATATAAAAAATGCCCTTGAAC

531-710 CTTTACTTCTTTTAAAATAACTTATTTTTTAAAGCCTAAATATAAAAAATGCCCTTGAAC

************************************************************

677-710 TATGCACTTTATGTCAAAAATATTTCTGAACTTTCAAAACTACCCTTAAACATTAAAAAA

531-710 TATGCACTTTATGTCAAAAATATTTCTGAACTTTCAAAACTACCCTTAAACATTAAAAAA

************************************************************

677-710 GAAAACAAAAATTCTCTTGCCGTTAGTTTTGGATGGAAACTGTTAAAGTTTTGTTTCAAA

531-710 GAAAACAAAAATTCTCTTGCCGTTAGTTTTGGATGGAAACTGTTAAAGTTTTGTTTCAAA

************************************************************

677-710 AATACCCCTAAACTTTTCAAAAGTTTAAAAAATACCATTAACTCAAAGAAGGTTAAAAAA

531-710 AATACCCCTAAACTTTTCAAAAGTTTAAAAAATACCATTAACTCAAAGAAGGTTAAAAAA

************************************************************

677-710 TACCTTCTTTGTAAGTATATGAACAGAAATCGTTAATACCACATTGCAAAAATAACTTTA

531-710 TACCTTCTTTGTAAGTATATGAACAGAAATCGTTAATACCACATTGCAAAAATAACTTTA

************************************************************

677-710 AAATTAAAAAAAAAAGTTTATAATTGCTCCTAACATTAATATAGTGATCAATTTGTTTAA

531-710 AAATTAAAAAAAAAAGTTTATAATTGCTCCTAACATTAATATAGTGATCAATTTGTTTAA

************************************************************

677-710 AGTTTTCTTAAGTTCGAAAATAATGAATTTAAAAACAAATTATATAGATATCCTCATTTT

531-710 AGTTTTCTTAAGTTCGAAAATAATGAATTTAAAAACAAATTATATAGATATCCTCATTTT

************************************************************

677-710 TTTTTTCATATAGATACTCTCATTTTTCTCTTCATTTTTCAATTCTAACTTACACAAATT

531-710 TTTTTTCATATAGATACTCTCATTTTTCTCTTCATTTTTCAATTCTAACTTACACAAATT

************************************************************

677-710 TTCTCGCCAACCAAAATTTTAAGTTAAAATATAAAATCCCATCAAATCTCACAAAATTCT

531-710 TTCTCGCCAACCAAAATTTTAAGTTAAAATATAAAATCCCATCAAATCTCACAAAATTCT

************************************************************

677-710 GAAATAAAAATTTTCTCTTAAACATAACAAAAAGAAAAAAATGTTAGCAGTTGAGTATGT

531-710 GAAATAAAAATTTTCTCTTAAACATAACAAAAAGAAAAAAATGTTAGCAGTTGAGTATGT

************************************************************

677-710 GTTAATAACTAAATACATTTTTTTATTTAACTATTTATCGTTTTGGTATGTTTAAGGGAA

531-710 GTTAATAACTAAATACATTTTTTTATTTAACTATTTATCGTTTTGGTATGTTTAAGGGAA

************************************************************

677-710 GATTTTGATTTTAGAATTTTGTGAGATTTTATGTTATTTTATGCTTAACTTACAATTTTG

531-710 GATTTTGATTTTAGAATTTTGTGAGATTTTATGTTATTTTATGCTTAACTTACAATTTTG

************************************************************

677-710 GTTAGTGAGAAAATTAGTGTAAGCTGAGAATTGGAAACTTAAGAGGAAAACAAAAGTATC

531-710 GTTAGTGAGAAAATTAGTGTAAGCTGAGAATTGGAAACTTAAGAGGAAAACAAAAGTATC

************************************************************

677-710 TATATAAAAAAAATGAGGATATCTCTATAATTTGTTTTAAAGTTCATTCTTTTTTAACTT

531-710 TATATAAAAAAAATGAGGATATCTCTATAATTTGTTTTAAAGTTCATTCTTTTTTAACTT

************************************************************

677-710 AAGAAAACTTCATACAAACCGATCACCATATTAATGTTATGAGCAATTTTAAACTTTTTG

531-710 AAGAAAACTTCATACAAACCGATCACCATATTAATGTTATGAGCAATTTTAAACTTTTTG

************************************************************

677-710 TTTTTTAATTTTTAAAGTTATTTTTGTAACGAGGTATTAACGGTTTCTGTTCATATACTA

531-710 TTTTTTAATTTTTAAAGTTATTTTTGTAACGAGGTATTAACGGTTTCTGTTCATATACTA

************************************************************

677-710 CGGTGAAGGTAAAAATGAATTTTTTTAGTTAACGGTATTTTTGAAACTTTTGAAAAGTTC

531-710 CGGTGAAGGTAAAAATGAATTTTTTTAGTTAACGGTATTTTTGAAACTTTTGAAAAGTTC

************************************************************

677-710 AAGGGTATTTTTGAAACAAACTTAAACGGCTTCCATCTAAAAATAACGACAAGGGTATTT

531-710 AAGGGTATTTTTGAAACAAACTTAAACGGCTTCCATCTAAAAATAACGACAAGGGTATTT

************************************************************

677-710 TCTTAACTCTTTTTGAAAGTTTAAGGATATTTTTGAAAAAAAATGCATAGTTCAAAGGCA

531-710 TCTTAACTCTTTTTGAAAGTTTAAGGATATTTTTGAAAAAAAATGCATAGTTCAAAGGCA

************************************************************

677-710 TTTTTTTTTAATATAATTTAACCTTTTTTAAATTAAACACTCGAAAAGTATTTCAAATAT

531-710 TTTTTTTTTAATATAATTTAACCTTTTTTAAATTAAACACTCGAAAAGTATTTCAAATAT

************************************************************

677-710 ACCCTAAATGTCAATTAATGTGTAATTTCTTTTGGAATTTTTATTTTAAATTAAAGTTAA

531-710 ACCCTAAATGTCAATTAATGTGTAATTTCTTTTGGAATTTTTATTTTAAATTAAAGTTAA

************************************************************

677-710 TTATATCGATTAATTAAGACTTCGTTTGATATATATATATATTTTTTGGCATATTAATTA

531-710 TTATATCGATTAATTAAGACTTCGTTTGATATATATATATATTTTTTGGCATATTAATTA

************************************************************

677-710 TAGAGTTTCCCACCGACTAGCAACCTTAACCCTAGTGACTATGGTGATCAAAACAGCAAG

531-710 TAGAGTTTCCCACCGACTAGCAACCTTAACCCTAGTGACTATGGTGATCAAAACAGCAAG

************************************************************

677-710 ATAACACCAAAACAGATTATGAATGGTATGGAGGGACTTACGGTAGACCAGGTAATCCTT

531-710 ATAACACCAAAACAGATTATGAATGGTATGGAGGGACTTACGGTAGACCAGGTAATCCTT

************************************************************

677-710 TTCTTTTCTTTTCCTAATTAAATAGACTTAGTACTTTCGATTTTTTTTTTTTTTTCATTC

531-710 TTCTTTTCTTTTCCTAATTAAATAGACTTAGTACTTTCGATTTTTTTTTTTTTTTCATTC

************************************************************

677-710 AAATCTGTCCCTAATCTTTTTAATATCATTTTTTATCGTTAACTCGAGCATAATCACTAT

531-710 AAATCTGTCCCTAATCTTTTTAATATCATTTTTTATCGTTAACTCGAGCATAATCACTAT

************************************************************

677-710 TTCAAAAGTTGATGATTTAATTCCTTTCATCCTCGTTATCGTTAACTGTTTGCATAATTG

531-710 TTCAAAAGTTGATGATTTAATTCCTTTCATCCTCGTTATCGTTAACTGTTTGCATAATTG

************************************************************

677-710 GACATTTTTCTATGATAATAAACAATTTTGAGAAGTTAAGTGGCTAAGTGCAAAGTCATG

531-710 GACATTTTTCTATGATAATAAACAATTTTGAGAAGTTAAGTGGCTAAGTGCAAAGTCATG

************************************************************

677-710 TAAATGAGTTTATTGTTTGGAAAGTAACAAAAATAGATTTCACTAGTAAAGCTCTATATA

531-710 TAAATGAGTTTATTGTTTGGAAAGTAACAAAAATAGATTTCACTAGTAAAGCTCTATATA

************************************************************

677-710 TATAATAGAAAAATTATTTTATATTAAATGATAAAAATTTTGAAAGTATTTACAAATAAT

531-710 TATAATAGAAAAATTATTTTATATTAAATGATAAAAATTTTGAAAGTATTTACAAATAAT

************************************************************

677-710 AGCAAAATATCACAGTTTATCTGTGAATAGACCGCAATAGTTCACAATAGACTACTATTT

531-710 AGCAAAATATCACAGTTTATCTGTGAATAGACCGCAATAGTTCACAATAGACTACTATTT

************************************************************

677-710 GTATCTATCATGACACAGGTAGACACAGATACTAGTCTATCGCTTTCTATCGCAGATAGA

531-710 GTATCTATCATGACACAGGTAGACACAGATACTAGTCTATCGCTTTCTATCGCAGATAGA

************************************************************

677-710 AAGTGAAATATTTTATATTTGTAAATATTTTAGTTCATTTTCTTATATTTGAAAACA

531-710 AAGTGAAATATTTTATATTTGTAAATATTTTAGTTCATTTTCTTATATTTGAAAACA

*********************************************************

**Supplementary Figure S2.** Nucleotide sequence similarity of ClCG02G015710 (710) between resistant parent USVL531-MDR (531) and susceptible parent USVL677-PMS (677). Sequences were aligned using Clustal Omega (<https://www.ebi.ac.uk/jdispatcher/msa/clustalo?stype=dna>).

677-720 TAACCATCGGCGTTCTTTCATCTTCTGATCTGCGACTCACGAACGGCCACCACTCCCATC

531-720 TAACCATCGGCGTTCTTTCATCTTCTGATCTGCGACTCACGAACGGCCACCACTCCCATC

************************************************************

677-720 AACTTCTCCGCCACCGCCGCCGCCGCTGTTCAAGGTTTGTATTTTTCTTTTCCTCTTTTT

531-720 AACTTCTCCGCCACCGCCGCCGCCGCTGTTCAAGGTTTGTATTTTTCTTTTCCTCTTTTT

************************************************************

677-720 TGATTTCTATTACTTAACTTATTAGTTTCAACTTCTTTCTTCAAGTTAAAGTTAAGTTTA

531-720 TGATTTCTATTACTTAACTTATTAGTTTCAACTTCTTTCTTCAAGTTAAAGTTAAGTTTA

************************************************************

677-720 AGTTAGATTTAGAAATATGTTCAACTAAATTAGCATTCTAACAATTAGCACAGAGTTTTT

531-720 AGTTAGATTTAGAAATATGTTCGACTAAATTAGCATTCTAACAATTAGCACAGAGTTTTT

********************** *************************************

677-720 TTTGACGTCCGAAATTATTAGGTAATATGTATTTCAGTAATTCTATTGTACTTTGTACTA

531-720 TTTGACGTCCGAAATTATTAGGTAATATGTATTTCAGTAATTCTATTGTACTTTGTACTA

************************************************************

677-720 TTGTCAATGAATTTGACATTTTATGCCTTTTAGTGAAATGCATTTCTATCCTAGCTTTTT

531-720 TTGTCAATGAATTTGACATTTTATGCCTTTTAGTGAAATGCATTTCTATCCTAGCTTTTT

************************************************************

677-720 AGAAATGAATGTATCACTGTTATCGAATCCGTTTCGTGAGGTTAAGTTTAATTATTCCCT

531-720 AGAAATGAATGTATCACTGTTATCGAATCCGTTTCGTGAGGTTAAGTTTAATTATTCCCT

************************************************************

677-720 CAATTTTCTTCAATTTCCACTCATCAATAGATTTGTAGCACTGAGAAGGCATCTAATATT

531-720 CAATTTTCTTCAATTTCCACTCATCAATAGATTTGTAGCACTGAGAAGGCATCTAATATT

************************************************************

677-720 TGTCCACCATTAGCAAGGTTGATTGAAACTCCATAATAATGTCTGAGAGGACAGGTTTGA

531-720 TGTCCACCATTAGCAAGGTTGATTGAAACTCCATAATAATGTCTGAGAGGACAGGTTTGA

************************************************************

677-720 GTTCTTTTAAATGCAGTCTAGCTACAATCTTACTGCCCCAGAATAAGACCGATAAGTCAA

531-720 GTTCTTTTAAATGCAGTCTAGCTACAATCTTACTGCCCCAGAATAAGACCGATAAGTCAA

************************************************************

677-720 GTGATAGGTTTGTGGTCATTTCTAGATTTGGCATTTGCTTTCTTGGGAATTATGCGAATT

531-720 GTGATAGGTTTGTGGTCATTTCTAGATTTGGCATTTGCTTTCTTGGGAATTATGCGAATT

************************************************************

677-720 TAAATCTCATTGAGGCTAGCATAAAAGATCTATTTCCAAAAATGTTGTGGGACTTGATCA

531-720 TAAATCTCATTGAGGCTAGCATAAAAGATCTATTTCCAAAAATGTTGTGGGACTTGATCA

************************************************************

677-720 GTGAGTCCGTAAGGTTTTGATGATGTTCATGAGTTTTTTAAAACGTCAACTGTGCAGCTC

531-720 GTGAGTCCGTAAGGTTTTGATGATGTTCATGAGTTTTTTAAAACGTCAACTGTGCAGCTC

************************************************************

677-720 CCCCACCCCCCGGGGAGCAAGAAACCCCAATTTATTAATATCGAACTGATTATAAACTCT

531-720 CCCCACCCCCCGGGGAGCAAGAAACCCCAATTTATTAATATCGAACTGATTATAAACTCT

************************************************************

677-720 ATTAATTTAGTCCTCATTTCCACTGTCATGGAATCACTAAGGTCCCATTTTGAATAAAAT

531-720 ATTAATTTAGTCCTCATTTCCACTGTCATGGAATCACTAAGGTCCCATTTTGAATAAAAT

************************************************************

677-720 AAAATGGTTATCAAATGTGGCCTAAGTAATTTTGATTTTTGAAATGCTCCATTTTTGTTG

531-720 AAAATGGTTATCAAATGTGGCCTAAGTAATTTTGATTTTTGAAATGCTCCATTTTTGTTG

************************************************************

677-720 GTACTTAATAATTTATATGAATTGGTATGTGCTCTTGCAAGGGATTGGCTTAAGCATTTG

531-720 GTACTTAATAATTTATATGAATTGGTATGTGCTCTTGCAAGGGATTGGCTTAAGCATTTG

************************************************************

677-720 AATGGAAATCTTTTTGACACATCCATATTGGTTTGTAATATCCATATTTGTTTGTTTCTC

531-720 AATGGAAATCTTTTTGACACATCCATATTGGTTTGTAATATCCATATTTGTTTGTTTCTC

************************************************************

677-720 TTTTTTTTTTCACTTTTTTTACATTCTTTTTTATTAGAAACAACAATGTTCATTGAGAAA

531-720 TTTTTTTTTTCACTTTTTTTAAATTCTTTTTTATTAGAAACAACAATGTTCATTGAGAAA

********************* **************************************

677-720 AAATGAAAGAATACAAGGACATACAAAATATCGAGCCCACAAAAGAGAAGGAAGAATCCC

531-720 AAATGAAAGAATACAAGGACATACAAAATATCGAGCCCACAAAAGAGAAGGAAGAATCCC

************************************************************

677-720 TCTAAAGGAAGAGACTCCAACTATACAAAATAGAACCCTATAGAATAGATACAAAATGTC

531-720 TCTAAAGGAAGAGACTCCAACTATACAAAATAGAACCCTATAGAATAGATACAAAATGTC

************************************************************

677-720 TTGGAAACTAAAGCCCAAAGAGAAACATCATATCTAACAAAGGGACAATACATCAAAAGA

531-720 TTGGAAACTAAAGCCCAAAGAGAAACATCATATCTAACAAAGGGACAATACATCAAAAGA

************************************************************

677-720 GTCACTCTCTAACTCCCGAACATCTTTTTATTCCCTTCCCCCTGGAAGACCCAAAGAACA

531-720 GTCACTCTCTAACTCCCGAACATCTTTTTATTCCCTTCCCCCTGGAAGACCCAAAGAACA

************************************************************

677-720 ACACACACCTTGGCCATCCATGAGAAAACGCTCCTTCTATCGAACAGTTGGATGAGGGAG

531-720 ACACGCACCTTGGCCATCCATGAGAAAACGCTCCTTCTATCGAACAGTTGGATGAGGGAG

**** *******************************************************

677-720 AAACTTTCCAATCATATCACTAGTAACCCTATGACGAGCATGCAAGAAGCCAAACGTATA

531-720 AAACTTTCCAATCATATCACTAGTAACCCTATGACGAGCATGCAAGAAGCCAAACGTATA

************************************************************

677-720 GAAGAAATAATCCCATATTGCTCTTGCAAACTCACAATGTTAGGGAATATGATCCAAGTC

531-720 GAAGAAATAATCCCATATTGCTCTTGCAAACTCACAATGTTAGGGAATATGATCCAAGTC

************************************************************

677-720 TTCCTCTGCCTTCCAACAAAGAATACAACAAAGCGGACCAACAAACGAGGGCAACTTCCT

531-720 TTCCTCTGCCTTCCAACAAAGAATACAACAAAGCGGACCAACAAACGAGGGCAACTTCCT

************************************************************

677-720 CGCAACGATCCATGGTGGATTAAACAATGCAAGTTGTTAACTATGAAGGATTGAACGATG

531-720 CGCAACGATCCATGGTGGATTAAACAATGCAAGTTGTTAACTATGAAGGATTGAACGATG

************************************************************

677-720 CAACCTTTTGAAAACTTGTTGCATATATGTTGCAAGTGTTTAATAAGATGTAAATAGTAG

531-720 CAACCTTTTGAAAACTTGTTGCATATATGTTGCAAGTGTTTAATAAGATGTAAATAGTAG

************************************************************

677-720 GCCTATTATTAGAATATACTGATTTGAGCATGTTGATTATGTAGTATCTGGTGGTTTTAT

531-720 GCCTATTATTAGAATATACTGATTTGAGCATGTTGATTATGTAGTATCTGGTGGTTTTAT

************************************************************

677-720 ATTGGAATTTGTTCATTTGATCTTCTATGTTTTACATGTCTGAAGCATCTTGTGACATAT

531-720 ATTGGAATTTGTTCATTTGATCTTCTATGTTTTACATGTCTGAAGCATCTTGTGACATAT

************************************************************

677-720 TTCCAGGATACACTACGTATACAATCATGCTTAATATTGCGGCCACGTTTTGCAAGCGAC

531-720 TTCCAGGATACACTACGTATACAATCATGCTTAATATTGCGGCCACGTTTTGCAAGCGAC

************************************************************

677-720 TGAATGTCAAGGATTTGGTGACCAATGTTCCTGTATATAGCAGTTTTAGTGGTAAGGATG

531-720 TGAATGTCAAGGATTTGGTGACCAATGTTCCTGTATATAGCAGTTTTAGTGGTAAGGATG

************************************************************

677-720 GCTCATCTAGAACAAGTTGGTGTCTGTCATGTTACTTTGACTGCGTATTTGATGATTACA

531-720 GCTCATCTAGAACAAGTTGGTGTCTGTCATGTTACTTTGACTGCGTATTTGATGATTACA

************************************************************

677-720 ATGTTTGTTGAATCAAATCTCAGATGGAGCTGCTGTTGGATTAAGCTTATTATTCAGACG

531-720 ATGTTTGTTGAATCAAATCTCAGATGGAGCTGCTGTTGGATTAAGCTTATTATTCAGACG

************************************************************

677-720 TTGGGCTACCAAAAAGACAGCTGGGTCGACTAAAAATGGACGAGACTCGAAACCCAAGAA

531-720 TTGGGCTACCAAAAAGACAGCTGGGTCGACTAAAAATGGACGAGACTCGAAACCCAAGAA

************************************************************

677-720 TCTTGGTGTAAAGAAGTTTGGTGGGGAGGTCAGTGGCAAGGAAACAAATCACGTTGTCTT

531-720 TCTTGGTGTAAAGAAGTTTGGTGGGGAGGTCAGTGGCAAGGAAACAAATCACGTTGTCTT

************************************************************

677-720 AAACTAGAACTGAATTTCTATTGATTGAAACTGTTTTGTTGTGAACTTATTTTACGGAGA

531-720 AAACTAGAACTGAATTTCTATTGATTGAAACTGTTTTGTTGTGAACTTATTTTACGGAGA

************************************************************

677-720 GTTGTTCCTTCTGGATACAGAGAGTTATTCCTGGTAACATTATCGTTCGTCAACGTGGCA

531-720 GTTGTTCCTTCTGGATACAGAGAGTTATTCCTGGTAACATTATCGTTCGTCAACGTGGCA

************************************************************

677-720 CTCGGTTTCATCCTGGAGATTATGTTGGAATAGGGAAGGATCACACTCTTTTTGCCCTGA

531-720 CTCGGTTTCATCCTGGAGATTATGTTGGAATAGGGAAGGATCACACTCTTTTTGCCCTGA

************************************************************

677-720 AAGAAGGCTGTGTCAAGTTTGAAAAGCACAAGCTGAGCGGACGTAAGTGGGTTCATGTTA

531-720 AAGAAGGCTGTGTCAAGTTTGAAAAGCACAAGCTGAGCGGACGTAAGTGGGTTCATGTTA

************************************************************

677-720 TTCCAAAGGAAGGGCACGTTCTTCATCCTGCTTATGCAACTACTTCTACTACCAAAGAAT

531-720 TTCCAAAGGAAGGGCACGTTCTTCATCCTGCTTATGCAACTACTTCTACTACCCCAGAAT

***************************************************** *****

677-720 TGAAGACAACATGATGTTTGAGTTCGTTGATGAACATGGTCATGAATGGTATAAATTTTC

531-720 TGAAGACAACATGATGTTTGAGTTCGTTGATGAACATGGTCATGAATGGTATAAATTTTC

************************************************************

677-720 TTGTAGTTTTTCATGGTCCACAGCAAATTCTGACTAAACTCGATCACCTCAAGAGGGGGT

531-720 TTGTAGTTTTTCATGGTCCACAGCAAATTCTGACTAAACTCGATCACCTCAAGAGGGGGT

************************************************************

677-720 GTAGGCAGTCTCTTCGAGGGCATGTTTTTAATGTTTCTAATTTCTTTTTCTGGGAACCCT

531-720 GTAGGCAGTCTCTTCGAGGGCATGTTTTTAATGTTTCTAATTTCTTTTTCTGGGAACCCT

************************************************************

677-720 ATCGTATTTCACTTTGGCTATTCTGCAGACGAAGATGTCCGGTCTTAGCTCGATGCACTT

531-720 ATCGTATTTCACTTTGGCTATTCTGCAGACGAAGATGTCCGGTCTTAGCTCGATGCACTT

************************************************************

677-720 AACAAATGAGAAATCGCACGACAAAGAGAGAAATTTCCTTAAGTTGCCTCCTTAAGTAAG

531-720 AACAAATGAGAAATCGCACGACAAAGAGAGAAATTTCCTTAAGTTGCCTCCTTAAGTAAG

************************************************************

677-720 TACAGGTGCACCGGACTGACAAATAATCGGTACCTCGAGTGTATTCAAGGTCCTCTTTCA

531-720 TACAGGTGCACCGGACTGACAAATAATCGGTACCTCGAGTGTATTCAAGGTCCTCTTTCA

************************************************************

677-720 ACTTCCTCTCCTCCCTGAATTTGTTTAGGGATATTTTCTTAATTGACATGCCAATGTGTA

531-720 ACTTCCTCTCCTCCCTGAATTTGTTTAGGGATATTTTCTTAATTGACATGCCAATGTGTA

************************************************************

677-720 CTCTTTTTTTTTTCCCCAGTAAATGATAGATCCACGGCTTAACCATGAGCATTAGTTATT

531-720 CTCTTTTTTTTTTCCCCAGTAAATGATAGATCCACGGCTTAACCATGAGCATTAGTTATT

************************************************************

677-720 AATTGCTTATTATATATATTTATGTATATATTGTTGCTATAAGAAGGTTC

531-720 AATTGCTTATTATATATATTTATGTATATATTGTTGCTATAAGAAGGTTC

**************************************************

**Supplementary Figure S3.** Nucleotide sequence similarity of ClCG02G015720 (720) between resistant parent USVL531-MDR (531) and susceptible parent USVL677-PMS (677). Sequences were aligned using Clustal Omega (<https://www.ebi.ac.uk/jdispatcher/msa/clustalo?stype=dna>).

677-730 TTAAATTGAAATGCTATTAGGAATTCCTCTACCTGTGAGACCTTCTGAACTAGTAGGAAA

531-730 TTAAATGGAAATGCTATTAGGAATTCCTCTACCTGTGAGACCTTCTGAACTAGTAGGAAA

****** *****************************************************

677-730 CAACAAAGTATATGGCATATCCACAGGTCCTACTCTATTTTTCAATTGAGGATCTTTATT

531-730 CAACAAAGTATATGGCATATCCACAGGTCCTACTCTATTTTTCAATTGAGGATCTTTATT

************************************************************

677-730 TCTCATAGCAATCTTCCCTTCAATTTCAGCCAACTTTTTCCCAAACTTCTCAAATGCTTC

531-730 TCTCATAGCAATCTTCCCTTCAATTTCAGCCAACTTTTTCCCAAACTTCTCAAATGCTTC

************************************************************

677-730 CAAAGCTTCCTTATCCAAAGTCCATTCAGGGTCGCTTCTTTGCCCTAAATACACTTCATC

531-730 CAAAGCTTCCTTATCCAAAGTCCATTCAGGGTCGCTTCTTTGCCCTAAATACACTTCATC

************************************************************

677-730 TGAAGAATGCCTTGACAAAATCTCAATAACTGACACCCCCATAAGAGCTTGTAATTGTGA

531-730 TGAAGAATGCCTTGACAAAATCTCAATAACTGACACCCCCACAAGAGCTTGTAATTGTGA

***************************************** ******************

677-730 AGTGATTGTTCTCAAGAACGCCTTTTCGGGATTCGTCTCGAGCTCTTTGTAGTCGGGAGT

531-730 AGTGATTGTTCTCAAGAACGCCTTTTCGGGATTCGTCTCGAGCTCTTTGTAGTCGGGAGT

************************************************************

677-730 ACCGTTTTCTGGTAGGAACCGACGACTAGTGGATGGTCTATTGGGAGCAAAGCCACCGTA

531-730 ACCGTTTTCTGGTAGGAACCGACGACTAGTGGATGGTCTATTGGGAGCAAAGCCACCGTA

************************************************************

677-730 AGGATATTGTCCAAAGTTAACTGCAGCATGAAGAGCTGAAGAGATCCATATAATGATTGT

531-730 AGGATATTGTCCAAAGTTAACTGCAGCATGAAGAGCTGAAGAGATCCATATAATGATTGT

************************************************************

677-730 ACAACTTTGTATTAGGTCTTGAACGGATTGCATTTTTGGCCACCATGGTTCATCTTTCTT

531-730 ACAACTTTGTATTAGGTCTTGAACGGATTGCATTTTTGGCCACCATGGTTCATCTTTCTT

************************************************************

677-730 ATCTGCATGGCCTTTCTCGCGAAGCTCTTTCCACCATGATTGGAGCTCTGTATCATTATG

531-730 ATCTGCATGGCCTTTCTCGCGAAGCTCTTTCCACCATGATTGGAGCTCTGTATCATTATG

************************************************************

677-730 TAGTGTTTGATCATCTTTGTAGTAGAAAGAGCAATAATCTTGTACCCATGTTTTGATTGC

531-730 TAGTGTTTGATCATCTTTGTAGTAGAAAGAGCAATAATCTTGTACCCATGTTTTGATTGC

************************************************************

677-730 TGACCAAATCTCAAGACCATCAACAGCATATGGATAATCTTCTATTAGTAGTTGAAGTCC

531-730 TGACCAAATCTCAAGACCATCAACAGCATATGGATAATCTTCTATTAGTAGTTGAAGTCC

************************************************************

677-730 ATGGGGAGAGCTTGAATCCTCTGTTGCAACTCCTCTTTAACAAATTAAGCCAAGGTTTGA

531-730 ATGGGGAGAGCTTGAATCCTCTGTTGCAACTCCTCTTTAACAAATTAAGCCAAGGTTTGA

************************************************************

677-730 ATGTTAATGAATGAAGTGAGAAAAAACAAGAGACAAAATAGTGTGTGATATATATATATA

531-730 ATGTTAATGAATGAAGTGAGAAAAAACAAGAGACAAAATAGTGTGTGATATATATATATA

************************************************************

677-730 TATATATAATAAAGTTATGTTATCATTTTATGATAGTGCTCAAGCAAGCTGGAATAGCTC

531-730 TATATATAATAAAGTTATGTTATCATTTTATGGTAGTGCTCAAGCAAGCTGGAATAGCTC

******************************** ***************************

677-730 AGTTGGTTAGAGCGTGTGGCTGTTAACCACAAGGTCGGAGGTTCAAGCCCTCCTTCTAGC

531-730 AGTTGGTTAGAGCGTGTGGCTGTTAACCACAAGGTCGGAGGTTCAAGCCCTCCTTCTAGC

************************************************************

677-730 GTTACTTTAAAAAGTAATAATAATTTTATAGAATTGAAAATATATATAATATAATCGACC

531-730 GTTTGTTTAAAAAGTAATAATAATTTTATAGAATTGAAAATATATATAATATAATCGACC

*** *******************************************************

677-730 TATTTTGTTTTAAACTTTCTTATATTTACAAATGTCCCTTAAACAAAGATGAGGACTATA

531-730 TATTTTGTTTTAAACTTTCTTATATTTACAAATGTCCCTTAAACAAAGATGAGGACTATA

************************************************************

677-730 GTAAAATTAAACTAATAAGCAAACATGGGAATTTCTTAACAAAATTCTAAAAACAAAACA

531-730 GTAAAATTAAACTAATAAGCAAACATGGGAATTTCTTAACAAAATTCTAAAAACAAAACA

************************************************************

677-730 AGTTTTTAAAAACTATTTTTGTAGTTTTCAAAACCCAACTTGAATTTTGAACCCAAAATA

531-730 AGTTTTTAAAAACTATTTTTGTAGTTTTCAAAACCCAACTTGAATTTTGAACCCAAAATA

************************************************************

677-730 AAGAAACCAATCATATAGATTTAATTTTCAAAAAAATATATATATATATATGTATGTCAT

531-730 AAGAAACCAATCATATAGATTTAATTTTCAAAAATATATATATATATATATGTATGTCAT

********************************** *************************

677-730 TATCAAAGGGGTCTAAATATTTTGTAATGATTACCTTTTGATTAGATCAGCAGGGAGAGC

531-730 TATCAAAGGGGTCTAAATATTTTGTAATGATTACCTTTTGATTAGATCAGCAGGGAGAGC

************************************************************

677-730 TTGTTGAGGGAAGACCCAAGTTTTGTAAACAAAAGAAGACAGCTCCATGGAGTATTTAGC

531-730 TTGTTGAGGGAAGACCCAAGTTTTGTAAACAAAAGAAGACAGCTCCATGGAGTATTTAGC

************************************************************

677-730 TGGATAATGAGTTGTTTCAATAATTCCATCAGCATTAATAAGAGATTGTCTAGCTAAAGC

531-730 TGGATAATGAGTTGTTTCAATAATTCCATCAGCATTAATAAGAGATTGTCTAGCTAAAGC

************************************************************

677-730 ATTAATGTTCATTGTGTCTCGAAAATGAGGAACAAGCAACTTGTGAATTGGATGAAGAAC

531-730 ATTAATGTTCATTGTGTCTCGAAAATGAGGAACAAGCAACTTGTGAATTGGATGAAGAAC

************************************************************

677-730 ACTTAATTGTCTGTTGGTTGCTATCACAAATGGTTCGATTGTTGCATGAGTATTCAACCT

531-730 ACTTAATTGTCTGTTGGTTGCTATCACAAATGGTTCGATTGTTGCATGAGTATTCAACCT

************************************************************

677-730 ATAAATTATAGAAAATGTAAACAAATATAAGTTCGGAGAAATATTATTCAACTTTATTGA

531-730 ATAAATTATAGAAAATGTAAACAAATATAAGTTCGGAGAAATATTATTCAACTTTATTGA

************************************************************

677-730 AGATATGTAGGTGTGTGAATTTACTCATAAATGGTGTGATTATAATTAGGTTTTGATAAG

531-730 AGATATGTAGGTGTGTGAATTTACTCATAAATGGTGTGATTATAATTAGGTTTTGATAAG

************************************************************

677-730 GGGAAAAAAAAAAGTGACAAATCAATATTTGTTTAAGGAAGAAATGTAAATCAACAAGGA

531-730 GGGAAAAAAAAAAGTGACAAATCAATATTTGTTTAAGGAAGAAATGTAAATCAACAAGGA

************************************************************

677-730 AAAAGAAAAAAATAAATAAATAAACCATCAAGGTAGACCAAGAATCAAACTAATAAAAAA

531-730 AAAAGAAAAAAATAAATAAATAAACCATCAAGGTAGACCAAGAATCAAACTAATAAAAAA

************************************************************

677-730 TAATCAAACTAATTATCAAAATATTTTTAATTTTTGGTTTCACCTCGAGCCTGTGTTCTT

531-730 TAATCAAACTAATTATCAAAATATTTTTAATTTTTGGTTTCACCTCGAGCCTGTGTTCTT

************************************************************

677-730 CTTTTAAAAAATATATTTGCGGTACACCCTAGACTGTCACCCATCTTTTTGACGTCCACG

531-730 CTTTTAAAAAATATATTTGCGGTACACCCTAGACTGTCACCCATCTTTTTGACGTCCACG

************************************************************

677-730 GAGTCATCCAACTATAGTTTACCTACGAAGAAAACTATTTTTTTTTTCTTTTCTGTTGGT

531-730 GAGTCATCCAACTATAGTTTACCTACGAAGAAAACTATTTTTTTTTTCTTTTCTGTTGGT

************************************************************

677-730 GGCTGGAGAGAGCAAAATAGATGGATGTAGTTCATAATGTATCTAAATATTTGTTTTTCT

531-730 GGCTGGAGAGAGCAAAATAGATGGATGTAGTTCATAATGTATCTAAATATTTGTTTTTCT

************************************************************

677-730 TCTTTTTAAATCTATAATATTAACCATATATGTCTTTGGTTTTATGAAAAACTCAACTAT

531-730 TCTTTTTAAATCTATAATATTAACCATATATGTCTTTGGTTTTATGAAAAACTCAACTAT

************************************************************

677-730 AAACAACAAGATTTTCCTTCTTCGACAAACAAAACTTTATTTGTTAGAGGGAGGAGGTTG

531-730 AAACAACAAGATTTTCCTTCTTCGACAAACAAAACTTTATTTGTTAGAGGGAGGAGGTTG

************************************************************

677-730 AGAGAGAGGGAGAAAGGAGGAAAGAAAGAAAAAACAATAAGAAAAAAATATTATATATAA

531-730 AGAGAGAGGGAGAAAGGAGGAAAGAAAGAAAAAACAATAAGAAAAAAATATTATATATAA

************************************************************

677-730 TTTAGTAATTAAATTATTTTTAAAAAAATAGGTGTCACATTAACTTGCAGTTGTTAGAAA

531-730 TTTAGTAATTAAATTATTTTTAAAAAAATAGGTGTCACATTAACTTGCAGTTGTTAGAAA

************************************************************

677-730 TTAAAAAAAAAAAAAAAAAATAGAGGGACCAAAGATATGCTACGAAACCCAGAACTCATA

531-730 TTAAAAAAAAAAAAAAAAAATAGAGGGACCAAAGATATGCTACGAAACCCAGAACTCATA

************************************************************

677-730 GCTAAAAAATTGTTGAGTTTAAAATTCAAGGACCCCAATGCAACTTAAACCAAATCTGGA

531-730 GCTAAAAAATTGTTGAGTTTAAAATTCAAGGACCCCAATGCAACTTAAACCAAATCTGGA

************************************************************

677-730 GACTAAAATTAAAGTTTAGGGTAAGAGAAAATTTTACCAATGGCTAATAAGTTGATGATA

531-730 GACTAAAATTAAAGTTTAGGGTAAGAGAAAATTTTACCAATGGCTAATAAGTTGATGATA

************************************************************

677-730 GCCAGTATCATTAACAGTAACATAAGCTTTAGCTAGCTGCCAAATTGTGCCATCAACTCC

531-730 GCCAGTATCATTAACAGTAACATAAGCTTTAGCTAGCTGCCAAATTGTGCCATCAACTCC

************************************************************

677-730 TGTCTTAGCAGGCAAAATTACTCTACTAATGACCCCAAATTTGTCCCCTTGAGGATGTGG

531-730 TGTCTTAGCAGGCAAAATTACTCTACTAATGACCCCAAATTTGTCCCCTTGAGGATGTGG

************************************************************

677-730 TAAGCTTAATTCAATTGCCAAAGGCTTTAAAGTCCCATCATTTTTCAAGAACAAAACTGT

531-730 TAAGCTTAATTCAATTGCCAAAGGCTTTAAAGTCCCATCATTTTTCAAGAACAAAACTGT

************************************************************

677-730 TCTTGTAGCATAAGTCTTTGTGGGAGTTGTATTTATTCTTCTAAGATATGGAATCAATGA

531-730 TCTTGTAGCATAAGTCTTTGTGGGAGTTGTATTTATTCTTCTAAGATATGGAATCAATGA

************************************************************

677-730 ATCATGGTGATTCAATATGTATAGCTTGTTCTTCTCAACTGCCTATCAAAAACAAATTTT

531-730 ATCATGGTGATTCAATATGTATAGCTTGTTCTTCTCAACTGCCTATCAAAAACAAATTTT

************************************************************

677-730 ATTTAAAGAAAAGTTTCAAAAAAGTAAGCAATCAACTCTAAAATTCTTAGTTTAATGTTT

531-730 ATTTAAAGAAAAGTTTCAAAAAAGTAAGCAATCAACTCTAAAATTCTTAGTTTAATGTTT

************************************************************

677-730 AATTACCTCATCCACAGTTAGCCCATCTAAGCCATCTTTTATGTGTTCTTCAGTTATTTT

531-730 AATTACCTCATCCACAGTTAGCCCATCTAAGCCATCTTTTATGTGTTCTTCAGTTATTTT

************************************************************

677-730 GCTTGTTTGATCACCATAAATGTTAGGATCCAGTTTGCTTGTAGGAGGAAATTCCTGTAA

531-730 GCTTGTTTGATCACCATAAATGTTAGGATCCAGTTTGCTTGTAGGAGGAAATTCCTGTAA

************************************************************

677-730 TTAAAAAACATGATATAAGCTTAATATTTGTAAATAAACTACAATTAAAAAAAAAAACAT

531-730 TTAAAAAACATGATATAAGCTTAATATTTGTAAATAAACTACAATTAAAAAAAAAAACAT

************************************************************

677-730 ATTAGGTTAAAATACGATTTTGATCCTTCTATTACGAGATTTCTATACTTTCAATAAGTA

531-730 ATTAGGTTAAAATACGATTTTGATCCTTCTATTACGAGATTTCTATACTTTCAATAAGTA

************************************************************

677-730 ATCTTAAATTTTAGTCCTTCAAAAGGCGCCTTTTTTTAAAAAATGATTAAATAATAATAA

531-730 ATCTTAAATTTTAGTCCTTCAAAAGGCGCCTTTTTTTAAAAAATGATTAAATAATAATAA

************************************************************

677-730 TAATAATAATAATTTCATGCAAGAAAATACAAATATACTAGTTTTATTTTTTAATTTTTT

531-730 TAATAATAATAATTTCATGCAAGAAAATACAAATATACTAGTTTTATTTTTTAATTTTTT

************************************************************

677-730 AGTGGAAAGACTCATAGATTAAAAGTAAGTTAGGAATCAACCATAAACTAGTAGTAGACA

531-730 AGTGGAAAGACTCATAGATTAAAAGTAAGTTAGGAATCAACCATAAACTAGTAGTAGAGA

********************************************************** *

677-730 CTAAATTAAAGATTTATTGAAAATACATAGACTAAAATTGTATTTTAACCAAATTATTTT

531-730 CTAAATTTAAGATTTATTGAAAATATATAGACTAAAATTGTATTTTAACCAAATTATTTT

******* ***************** **********************************

677-730 TATAAAAAAAAATTACTTGAAGACGAGCAATAACTACGGGGTTAACTCCGGCCAACATTT

531-730 TATAAAAAAAAATTACTTGAAGACGAGCAATAACTACGGGGTTAACTCCGGCCAACATTT

************************************************************

677-730 CTCTTGCAAATTCTTCATCAGTGCTCCATGCAAACTTATCCTCTATTCCAAGTTATTGAA

531-730 CTCTTGCAAATTCTTCATCAGTGCTCCATGCAAACTTATCCTCTATTCCAAGTTATTGAA

************************************************************

677-730 ATCACCAAAAAAAAAAAAAAAAAAAAAAAAAAAAAAAAAAAAAAAAAAAAAAANNNNNNN

531-730 ATCACCAAAAAAAAAAAAAAAAAAAAAAAAAAAAAAAAAAAAAAAAAAAAAAANNNNNNN

************************************************************

677-730 NNNNNNNNNNNNNNNNNNNNNNNNNNNNNNNNNNNNNNNNNNNNNNNNNNNNNNNNNNNN

531-730 NNNNNNNNNNNNNNNNNNNNNNNNNNNNNNNNNNNNNNNNNNNNNNNNNNNNNNNNNNNN

************************************************************

677-730 NNNNNNNNNNNNNNNNNNNNNNNNNNNNNNNNNNNNNNNNNNNNNNNNNNNNNNNNNNNN

531-730 NNNNNNNNNNNNNNNNNNNNNNNNNNNNNNNNNNNNNNNNNNNNNNNNNNNNNNNNNNNN

************************************************************

677-730 NNNNNNNNNNNNNNNNNNNNNNNNNNNNNNNNNNNNNNNNNNNNNNNNNNNNNNNNNNNN

531-730 NNNNNNNNNNNNNNNNNNNNNNNNNNNNNNNNNNNNNNNNNNNNNNNNNNNNNNNNNNNN

************************************************************

677-730 NNNNNNNNNNNNNNNNNNNNNNNNNNNNNNNNNNNNNNNNNNNNNNNNNNNNNNNNNNNN

531-730 NNNNNNNNNNNNNNNNNNNNNNNNNNNNNNNNNNNNNNNNNNNNNNNNNNNNNNNNNNNN

************************************************************

677-730 NNNNNNNNNNNNNNNNNNCCAAAAAAAAAAAAAAAAAAAAAAAGAACACACAGTAAGTTA

531-730 NNNNNNNNNNNNNNNNNNCCAAAAAAAAAAAAAAAAAAAAAAAGAACACACAGTAAGTTA

************************************************************

677-730 TCCATACAAACAAATTTAATAATAATAAAAAATATATATATATATATTAGTTGCAATTAT

531-730 TCCATACAAACAAATTTAATAATAATAAAAAATATATATATATATATTAGTTGCAATTAT

************************************************************

677-730 TTTACTTTGGGAGGACTCTGTACTAAAACAAGAAAAGCAAGAAAAAGAAAAACATACCAT

531-730 TTTACTTTGGGAGGACTCTGTACTAAAACAAGAAAAGCAAGAAAAAGAAAAACATACCAT

************************************************************

677-730 GGATTAGCTGAGGAGTTGGGAATCTAAGAAATCTTTCACCATCAGTTCTAAAGATTTCTT

531-730 GGATTAGCTGAGGAGTTGGGAATCTAAGAAATCTTTCACCATCAGTTCTAAAGATTTCTT

************************************************************

677-730 TAAGCAATGGAGCTGGAATGTTGTCAGTGATACTCTCAAGCAAAGTTCTTGGAACAGGCA

531-730 TAAGCAATGGAGCTGGAATGTTGTCAGTGATACTCTCAAGCAAAGTTCTTGGAACAGGCA

************************************************************

677-730 AACCTTTTTCAAAGAGGTCAAAGACATCATTAAAGTCATCAAATTCCCCTGGGGTGCTAT

531-730 AACCTTTTTCAAAGAGGTCAAAGACATCATTAAAGTCATCAAATTCCCCTGGGGTGCTAT

************************************************************

677-730 CAAATAGATCATCCAATTTTGGTTTGATTGATCTTGAAATTGATTTTAGGCCATAAGCAA

531-730 CAAATAGATCATCCAATTTTGGTTTGATTGATCTTGAAATTGATTTTAGGCCATAAGCAA

************************************************************

677-730 GAAAGTCTGACATCTTCAAGTGACCAAATCTTTCATCTCTTGGAACGTAAATATCAGTTG

531-730 GAAAGTCTGACATCTTCAAGTGACCAAATCTTTCATCTCTTGGAACGTAAATATCAGTTG

************************************************************

677-730 GTTTACTTGAACCTGGTAATCTACTTTCACTTTTGGGATCTGCATGTTCAAATCACAATA

531-730 GTTTACTTGAACCTGGTAATCTACTTTCACTTTTGGGATCTGCATGTTCAAATCACAATA

************************************************************

677-730 GAAGTTCAACATACCTTTAATATTAAAACGTTGTTTCATCATTGCCAACTTTCCTCCCTA

531-730 GAAGTTCAACATACCTTTAATATTAAAACGTTGTTTCATCATTGCCAACTTTCCTCCCTA

************************************************************

677-730 ACTCAATTGGTTAAGATATATATTATCAATTTGAAAGATTGATGAATTTCAAATCACGAC

531-730 ACTCAATTGGTTAAGATATATATTATCAATTTGAAAGATTGATGAATTTCAAATCACGAC

************************************************************

677-730 TACTAGATGGTAATAAGTAAAAATTAAAAAAAAACAAAAAAAAAAACCATAAATATACCT

531-730 TACTAGATGGTAATAAGTAAAAATTAAAAAAAAAACAAAAAAAAAACCATAAATATACCT

********************************** ************************

677-730 GAACATCGGACCTCTCGATAAAAAAGTCTTATATGTTTTTTAAACAAGTTTAATTATATC

531-730 GAACATCGGACCTCTCGATAAAAAAGTCTTATATGTTTTTTAAACAAGTTTAATTATATC

************************************************************

677-730 TACATACATACAAGAATTTTACATTTGGAGTTAATCACAATATTTCTGTTATATGTGGTA

531-730 TACATACATACAAGAATTTTACATTTGGAGTTAATCACAATATTTCTGTTATATGTGGTA

************************************************************

677-730 AAATATAACGGAAAAAAAAAAAAAGTCTACAAATATCACTAATGTGAATGTAATATTAAT

531-730 AAATATAACGAAAAAAAAAAAAAAGTCTACAAATATCACTAATGTGAATGTAATATTAAT

********** *************************************************

677-730 TTACGATTTTTTGGTTAATTAATTACAAGTTTAGTCTTTAAATTGTGAGGTGTGTGCTTT

531-730 TTACGATTTTTTGGTTAATTAATTACAAGTTTAGTCTTTAAATTGTGAGGTGTGTGCTTT

************************************************************

677-730 CGTCATTGAACTTTCAATATTATATCTAATAGAGGTCCTTGACAAATTCGAAAATTAATT

531-730 CGTCATTGAACTTTCAATATTATATCTAATAGAGGTCCTTGACAAATTCGAAAATTAATT

************************************************************

677-730 GAATTATTAAATATAAATATAAGTTTTATATCGAATAATGAAATTCTCATCTTTCAATTA

531-730 GAATTATTAAATATAAATATAAGTTTTATATCGAATAATGAAATTCTCATCTTTCAATTA

************************************************************

677-730 TTTTGAAATTTAAGGACTAAAGAGATACAAACTGATTGAATTTGCAATTTAACCTTGATT

531-730 TTTTGAAATTTAAGGACTAAAGAGATACAAACTGATTGAATTTGCAATTTAACCTTGATT

************************************************************

677-730 AATTTTAAGCCATTTTGAAGGTGAAATGCTTATGATATTAATATATGTTTAGTTTTTTCT

531-730 AATTTTAAGCCATTTTGAAGGTGAAATGCTTATGATATTAATATATGTTTAGTTTTTTCT

************************************************************

677-730 ATTAACTTTTTATTGATATTTCTATTAACATTTAATCTTCCTAAAACCTAAGATGTTTCC

531-730 ATTAACTTTTTATTGATATTTCTATTAACATTTAATCTTCCTAAAACCTAAGATGTTTCC

************************************************************

677-730 TTCACCATCAAAATATAAACCATTGTAAAAATAAAAAAATAAAAAAATAAAACATTTAAA

531-730 TTCACCATCAAAATATAAACCATTGTAAAAATAAAAAAATAAAAAAATAAAACATTTAAA

************************************************************

677-730 AAAAGTAATATAAAAATTTTGCCACCAACTTCTAAATCATTTGAGATAGGAAAAAAATGA

531-730 AAAAGTAATATAAAAATTTTGCCACCAACTTCTAAATCATTTGAGATAGGAAAAAAATGA

************************************************************

677-730 GCAACGTGCATGATATAAAACAATACACAATTATCATCATCTAGAGTGAAAAAAAAAAAT

531-730 GCAACGTGCATGATATAAAACAATACACAATTATCATCATCTAGAGTGAAAAAAAAAAAT

************************************************************

677-730 CAAAATGAACATAGTTCAACTGGCATACGAATGTGTTAATGACAAAAAGATTTGTAGTTT

531-730 CAAAATGAACATAGTTCAACTGGCATACGAATGTGTTAATGACAAAAAGATTTGTAGTTT

************************************************************

677-730 GAATTTCTCTATCTCTATTGTACTAAAAAATAGAGAGTGAAAAAATCAAGAAAAAAATTA

531-730 GAATTTCTCTATCTCTATTGTACTAAAAAATAGAGAGTGAAAAAATCAAGAAAAAAATTA

************************************************************

677-730 AAAAAAAATCAAACATTAATATGATGATGAAATAATTAATTGATTAATTAAACTTAGTAG

531-730 AAAAAAAATCAAACATTAATATGATGATGAAATAATTAATTGATTAATTAAACTTAGTAG

************************************************************

677-730 TAATTATTAATGATTTAGGAATGTATATAATTGTGATTTAAAAATTTTTACTACATTTAA

531-730 TAATTATTAATGATTTAGGAATGTATATAATTGTGATTTAAAAATTTTTACTACATTTAA

************************************************************

677-730 CTATTGTCTAGACCACACAAATATAGATATTTTTGAGTGAACACAAAGATAGATGTTGAG

531-730 CTATTGTCTAGACCACACAAATATAGATATTTTTGAGTGAACACAAAGATAGATGTTGAG

************************************************************

677-730 GTTAGATATTATCAAAGCTTTTCTTTAAAAAAAAAAATAAAAATATATGTATATATAATT

531-730 GTTAGATATTATCAAAGCTTTTCTTTTTAAAAAAAAATAAAAATATATGTATATATAATT

************************** ********************************

677-730 TACCTTTCTTGGAAGGTGGTCTTCCCGTTCTTCCTCTACGAGGGTACGGATATTTGGATG

531-730 TACCTTTCTTGGAAGGTGGTCTTCCCGTTCTTCCTCTACGAGGGTACGGATATTTGGATG

************************************************************

677-730 AACCTCCAAGTATAGGACGACCAAGGTCCAAATTACTATCTGGGTCACCAATATCATTGT

531-730 AACCTCCAAGTATAGGACGACCAAGGTCCAAATTACTATCTGGGTCACCAATATCATTGT

************************************************************

677-730 ATACATCATAATCATAAATTCTATCCCATTCCTTTCTCTCTCCTTTTCCATTTCCTCTAA

531-730 ATACATCATAATCATAAATTCTATCCCATTCCTTTCTCTCTCCTTTTCCATTTCCTCTAA

************************************************************

677-730 GATTCAATAGTTCATCAGCCCTATATTTGCGAAGTGGCTCCGGTGTTTCATTAGGAAGGT

531-730 GATTCAATAGTTCATCAGCCCTATATTTGCGAAGTGGCTCCGGTGTTTCATTAGGAAGGT

************************************************************

677-730 ATGCCTGTATTCAAATTATCGATATTGTCAACCCAAACATAGCTCAGTTGATCAAAACAT

531-730 ATGCCTGTATTCAAATTATCGATATTGTCAACCCAAACATAGCTCAGTTGATCAAAACAT

************************************************************

677-730 TATATATACACTCATTCAAAAAGCAATGGTTTGTGGTGAGTATCACACAATTGGTTTTGA

531-730 TATATATACACTCATTCAAAAAGCAATGGTTTGTGGTGAGTATCACACAATTGGTTTTGA

************************************************************

677-730 GAACTAATCCTCTATGTTTATATAATATGGTATCAAAACAACATATGAATTACCTAAATG

531-730 GAACTAATCCTCTATGTTTATATAATATGGTATCAAAACAACATATGAATTACCTAAATG

************************************************************

677-730 GACATTTGATTCTTTAAAAAAAAAAAAAAAGTTCAGAAATAACCAACTCCAAAGAGATTG

531-730 GACATTTGATTCTTTAAAAAAAAAAAAAAAGTTCAGAAATAACCAACTCCAAAGAGATTG

************************************************************

677-730 AGGTCAATTATCTCACATTGAAAAAATGAAGAGGATGACTCACAATTTTTGAAATATACG

531-730 AGGTCAATTATCTCACATTGAAAAAATGAAGAGGATGACTCACAATTTTTGAAATATACG

************************************************************

677-730 TGAGATATATACCAATCCACTCCTACTCTTAAACTATAAAAACATGAATTGTCGAATATT

531-730 TGAGATATATACCAATCCACTCCTACTCTTAAACTATAAAAACATGAATTGTCGAATATT

************************************************************

677-730 TAATCAACTATATTTTTTTTCTTTAATGTTTAAGGGTCGTTTGAAAAAATGGAGAGATCA

531-730 TAATCAACTATATTTTTTTTCTTTAATGTTTAAGGGTCGTTTGAAAAAATGGAGAGATCA

************************************************************

677-730 CCTCACATTCTTTATAAAATAGATGGGTTAATCTTCTCATTGCAATAGTTTTGAAATTGA

531-730 CCTCACATTCTTTATAAAATAGATGGGTTAATCTTCTCATTGCAATAGTTTTGAAATTGA

************************************************************

677-730 ATATACAGGTTTGTTTAGTAGGTTAGCAACTTTTGACCTTTGACTTTTTTTTAATTATTT

531-730 ATATACAGGTTTGTTTAGTAGGTTAGCAACTTTTGACCTTTGACTTTTTTTTAATTATTT

************************************************************

677-730 TTTAGATGTGGTACTTCAAAACTAATAAAATGTGAAGCAAAATATTTTAAAGGGTACACT

531-730 TTTAGATGTGGTACTTCAAAACTAATAAAATGTGAAGCAAAATATTTTAAAGGGTACACT

************************************************************

677-730 TCGATTATAAATATGCAAATTGCTCTTATATCTTAATTAATTCCATAGTCAAGGGCCGAG

531-730 TCGATTATAAATATGCAAATTGCTCTTATATCTTAATTAATTCCATAGTCAAGGGCCGAG

************************************************************

677-730 GAAGTCCTTTTATTTTAAAATATTAATTATCCCAAATATATAGGCTAAGCTAGGGATTAT

531-730 GAAGTCCTTTTATTTTAAAATATTAATTATCCCAAATATATAGGCTAAGCTAGGGATTAT

************************************************************

677-730 GCCAAAGCGAGCTTAGCTCAGCAATAATTGGCATAACGTTCCATCCTCGAGGTGGAAGGT

531-730 GCCAAAGCGAGCTTAGCTCAGCAATAATTGGCATAACGTTCCATCCTCGAGGTGGAAGGT

************************************************************

677-730 TCGAATCCTCCATCTCCCACTTGTTGTACTAAAAAAAAAAAAAAAATCTAGAAATCATAT

531-730 TCGAATCCTCCATCTCCCACTTGTTGTACTAAAAAAAAAAAAAAAATCTAGAAATCATAT

************************************************************

677-730 ATTAAAGTGTCATTATCATGCAAAACCAGGTGCCTTATACCATATTATAATATTTAAATC

531-730 ATTAAAGTGTCATTATCATGCAAAACCAGGTGCCTTATACCATATTATAATATTTAAATC

************************************************************

677-730 ATCAAGATAGCTAAGTTGATAGGTGATTAAACATAGATTACTACAATAGTTGATGAGCAC

531-730 ATCAAGATAGCTAAGTTGATAGGTGATTAAACATAGATTACTACAATAGTTGATGAGCAC

************************************************************

677-730 AATAAACTTTTCTTTTCCTTTTAATAATTCTTAAATAAAAAGTAAAAAGCTAAAAATATA

531-730 AATAAACTTTTCTTTTCCTTTTAATAATTCTTAAATAAAAAGTAAAAAGCTAAAAATATA

************************************************************

677-730 TATAATTTAATTTCTAATATTAGTGTGATCATTATCCAACTTATGTGTTGAAAATTTTAG

531-730 TATAATTTAATTTCTAATATTAGTGTGATCATTATCCAACTTATGTGTTGAAAATTTTAG

************************************************************

677-730 ATTAATTTTTTTTTACACATTTATATTGAATTTTTTTTAATCAAATCTTTCCCTCTAGCT

531-730 ATTAATTTTTTTTTACACATTTATATTGAATTTTTTTTAATCAAATCTTTCCCTCTAGCT

************************************************************

677-730 AAATGTGTTAAAGCTCAAGGATGCAACAATGATGTAAAATTAAGATATATTTTTATTATC

531-730 AAATGTGTTAAAGCTCAAGGATGCAACAATGATGTAAAATTAAGATATATTTTTATTATC

************************************************************

677-730 TTAGTGAGAAATATCTTGTGTTTTCATTTCTCAAATTAAAAAAAAAAAAAAAGTTACAAA

531-730 TTAGTGAGAAATATCTTGTGTTTTCATTTCTCAAATTAAAAAAAAAAAAAAAGTTACAAA

************************************************************

677-730 ATCAAACATGTCAATTTTACATTGCCTTAGTCTTTAAAACCTTACTTCGACATGAAATAG

531-730 ATCAAACATGTCAATTTTACATTGCCTTAGTCTTTAAAACCTTACTTCGACATGAAATAG

************************************************************

677-730 TAAATTACGAGGTTATTATATAATGATAATTGATAGCTTCATAACAAGTATCGTGGTTTT

531-730 TAAATTACGAGGTTATTATATAATGATAATTGATAGCTTCATAACAAGTATCGTGGTTTT

************************************************************

677-730 CAAATTCATGCAATACCATTTTTGTAACGTTTTAATGATGGATAAGGACTTGATCATGAA

531-730 CAAATTCATGCAATACCATTTTTGTAACGTTTTAATGATGGATAAGGACTTGATCATGAA

************************************************************

677-730 ACTTTACCTGATTGGCAAAAAATATACGATCATTTTTGTATTTATCAGCAGGATAAACCC

531-730 ACTTTACCTGATTGGCAAAAAATATACGATCATTTTTGTATTTATCAGCAGGATAAACCC

************************************************************

677-730 AAGAATTGCAATCAAAATGAAGTCTACCATGGCCAGGGACATCCTCAAGAGTGAGAGATT

531-730 AAGAATTGCAATCAAAATGAAGTCTACCATGGCCAGGGACATCCTCAAGAGTGAGAGATT

************************************************************

677-730 TGAGAAAGAATTCACTAAAATGTTCATTTCTAATGAAGAAAGCACCTGGAACTCCAATTT

531-730 TGAGAAAGAATTCACTAAAATGTTCATTTCTAATGAAGAAAGCACCTGGAACTCCAATTT

************************************************************

677-730 CTTCATCCCAATCAAATGTAATACTGAAGGCTGTTTCTCCAGCAAAAATTGGGATTATTG

531-730 CTTCATCCCAATCAAATGTAATACTGAAGGCTGTTTCTCCAGCAAAAATTGGGATTATTG

************************************************************

677-730 TATTCCCAACCCAATTCTCCAAGTATGCTGCCTCTCCCACTTTCCCTTCAAAATCTTTAG

531-730 TATTCCCAACCCAATTCTCCAAGTATGCTGCCTCTCCCACTTTCCCTTCAAAATCTTTAG

************************************************************

677-730 CTGTTTTGTTGAATACCAAAATTAAAAATTAATAATAAATAAATAAATATTCCAAGGTTG

531-730 CTGTTTTGTTGAATACCAAAATTAAAAATTAATAATAAATAAATAAATATTCCAAGGTTG

************************************************************

677-730 TTCGTCTCTACTTGAAATCATGAATAAAATATCCTTAATGTTACACATTACACAGTCTTT

531-730 TTCGTCTCTACTTGAAATCATGAATAAAATATCCTTAATGTTACACATTACACAGTCTTT

************************************************************

677-730 AAAATAAAAACAATAAAAAAATACTTGTCAACCTCTGTTGATTTCTTTGTTTTGTTATCT

531-730 AAAATAAAAACAATAAAAAAATACTTGTCAACCTCTGTTGATTTCTTTGTTTTGTTATCT

************************************************************

677-730 ACACTTTAAGAGTGTTTTAAACATTCAAGTCAAGTTTTAAAAACTAAAAAAATTAAAATA

531-730 ACACTTTAAGAGTGTTTTAAACATTCAAGTCAAGTTTTAAAAACTAAAAAAATTAAAATA

************************************************************

677-730 AAATGTTTTTATTTTAGAATTGGACAAAAGGTTCAATTGTTTACTTAAGGAATATGAGGA

531-730 AAATGTTTTTATTTTAGAATTGGACAAAAGGTTCAATTGTTTACTTAAGGAATATGAGGA

************************************************************

677-730 AACAAAGTTAATTTTAAAAAATGCAAAAAACTAAAAACTAAGGCCCCATTTGATAACCAT

531-730 AACAAAGTTAATTTTAAAAAACTCAAAAAACTAAAAACTAAGGCCCCATTTGATAACCAT

********************* *************************************

677-730 TTCACATTTTGTTTTTATTTTTTTTTTAAATTGAGCCTACACATACTACTTGCATCTATT

531-730 TTCACATTTTGTTTTTATTTTTTTTTTAAATTGAGCCTACACATACTACTTGCACCTATT

****************************************************** *****

677-730 GATTTTTTCTTCTTTTGATGTCTATGTTTTAGGAGTGCTTTCAAAAACCAATCAAATAGT

531-730 GATTTTTTCTTCTTTTGATGTCTATGTTTTAGGAGTGCTTTCAAAAACCAATCAAATAGT

************************************************************

677-730 AAATTAAAAAAATTTTTAAAATTAAAAAAAAAAAAAAAACTGTTTTTTAAATTTGACTTA

531-730 AAATTAAAAAAATTTTTAAAATTAAAAAAAAAAAAAAAACTGTTTTTTAAATTTGACTTA

************************************************************

677-730 GAATTCAAATGTTTAGTTAGAAAATATGAAAATCATATTAAAAAATTTGAAGAAAAAAAA

531-730 GAATTCAAATGTTTAGTTAGAAAATATGAAAATCATATTAAAAAATTTGAAGAAAAAAAA

************************************************************

677-730 AAGCGTAAATTTTATATTAAAAAAATTGAATGCAAGTATATGATGAGGATGCTAAAAATG

531-730 AAACGTAAATTTTATATTAAAAAAATTGAATGCAAGTATATGATGAGGATGCTAAAAACG

** ******************************************************* *

677-730 TGTCAATCTAGTTGAGATATCTAGGTCCATCTATTGATATCCCCACTAGTACTTTGTTAA

531-730 TGTCAATCTAGTTGAGATATCTAGGTCCATCTATTGATATCCCCACTAGTACTTTATTAA

******************************************************* ****

677-730 AAAAAAAAAAGAAAAAATGGATGCAAAATAAATACCAACCGAGCCCTAAATGGTTAGCAA

531-730 AAAAAAAAAAAAAAAAATGGATGCAAAATAAATACCAACCCAGCCCTAAATGGTTAGCAA

********** ***************************** *******************

677-730 GAACAAGACTAATTTTTTGTTTGATAAAGAATTGAAATTGAATATAAAGAAGTTCAAGAA

531-730 GAACAAGACTAATTTTTTGTTTGATAAAGAATTGAAATTGAATATAAAGAAGTTCAAGAA

************************************************************

677-730 AATGTTAAATCAATCGTTATCGTAACAAATTTAAAAGTCGCCTTTAAATTCATTCATTTT

531-730 AATGTTAAATCAATCGTTATCGTAACAAATTTAAAAGTCGCCTTTAAATTCATTCATTTT

************************************************************

677-730 AAAAATAAAAAAATAAAATTAAAAAAAAAAAAAAACAACTTGGTCTCTCCAAAGACTTTC

531-730 AAAAATAAAAAAATAAAATAAAATAAAAAAAAAAACAACTTGGTCTCTCCAAAGACTTTC

******************* *** ************************************

677-730 ACACTTTTGTATTGGTCTTTGTATTAGCCAAAATAATTAAATTATTGTAGTTTACTTTAT

531-730 ACACTTTTGTATTGGTCTTTGTATTAGCCAAAATAATTAAATTATTGTAGTTTACTTTAT

************************************************************

677-730 GTGTAGAGTGTTTAATAAAAAATTTATTGAGTATTATGTGAATTTGAATGTTTGTATATA

531-730 GTGTAGAGTGTTTAATAAAAAATTTATTGAGTATTATGTGAATTTGAATGTTTGTATATA

************************************************************

677-730 TATCGTGTTGAAATCATTAATGATGTAAGATATTTTAAATAAATTATGAAAAATTCAATG

531-730 TATCGTGTTGAAATCATTAATGATGTAAGATATTTTAAATAAATTATGAAAAATTCAATG

************************************************************

677-730 ACAAGTAATATATATTGAAAGAAATTTTTATAAAAATTTAAGAAGAAAAACAGTTTAATT

531-730 ACAAGTAATATATATTGAAAGAAATTTTTATAAAAATTTAAGAAGAAAAACAGTTTAATT

************************************************************

677-730 TTCATACTTCACGTATTGTAAAAAAAAAAAAAAAAAAGTAAAAAATGAAAATAATATAAT

531-730 TTCATACTTCACGTATTGTAAAAAAAAAAAAAAAAAAGTAAAAAATGAAAATAATATAAT

************************************************************

677-730 ATAGCAATTTAGTTTATAAATATCGAAAAAAAAAATGTAAAGTATTCCCTAGATCAAAAT

531-730 ATAGCAATTTAGTTTATAAATATCGAAAAAAAAAATGTAAAGTATTCCCTAGATCAAAAT

************************************************************

677-730 AAAATTTGCCCATTTGATGTATTTAAGCTTTTATTTTTTATTTTTTTTCCTTCAAACTTG

531-730 AAAATTTGCCCATTTGATGTATTTAAGCTTTTATTTTTTATTTTTTTTCCTTCAAACTTG

************************************************************

677-730 AATATCTAATAATCAATAAACAAAACATTTGGGTGTCAAGAAAACTCATAGAATATTAAA

531-730 AATATCTAATAATCAATAAACAAAACATTTGGGTGTCAAGAAAACTCATAGAATATTAAA

************************************************************

677-730 TCTTAAGTAGGTCGCCACCATAGATTAAATTTATAACCCTTTCGCTCTTTGGACTTTTTA

531-730 TCTTAAGTAGGTCGCCACCATAGATTAAATTTATAACCCTTTCGCTCTTTGGACTTTTTA

************************************************************

677-730 AGATATTTCCACTACCACTAGACCAACCCATGATGGTTAAGACATTTATGTATTTATGTT

531-730 AGATATTTCCACTACCACTAGACCAACCCATGATGGTTAAGACATTTATGTATTTATGTT

************************************************************

677-730 TTTTTTTCTTCTGAACAAGATACTAGTAAACAGGATATTAGGTGTATCAGGATATCTTAA

531-730 TTTTTTTCTTCTGAACAAGATACTAGTAAACAGGATATTAGGTGTATCAGGATATCTTAA

************************************************************

677-730 CTAGCTTGACACATCGTGACACCCTCATCATATCCCAAATTGAGTCGATATGTTATCAAA

531-730 CTAGCTTGACACATCGTGACACCCTCATCATATCCCAAATTGAGTCGATATGTTATCAAA

************************************************************

677-730 AGAATGAGAATAGAGAGGTTCAAAATTTACAATAGAGCTAGAGGAAACCTCATCACAAAA

531-730 AGAATGAGAATAGAGAGGTTCAAAATTTACAATAGAGCTAGAGGAAACTTCATCACAAAA

************************************************ ***********

677-730 GAAAGATGAAATATGCAAGATTAGAGGAAAGCTTTTCAATTAAGAACAATTATGGTAACA

531-730 GAAAGATGAAATATGCAAGATTAGAGGAAAGCTTTTCAATTAAGAACAATTATGGTAACA

************************************************************

677-730 TCATAATTAAAAAAAGAGATTAGATTTGGAGGTCCAAAGCACAACCAAAATAAATGATAT

531-730 TCATAATTAAAAAAAGAGATTAGATTTGGAGGTCCAAAGCACAACCAAAATAAATGATAT

************************************************************

677-730 CATGACATAGATGATTAAGAGATCTATCAACACCTTGGAACGTCCTCCTATTTCATATAT

531-730 CATGACATAGATGATTAAGAGATCTATCAACACCTTGGAACGTCCTCCTATTTCATATAT

************************************************************

677-730 GTGTTTATGTGTTGAATAATATTTTTAAAAAAAGTAACAGAAAAAGACAAATATTTCTTT

531-730 GTGTTTATGTGTTGAATAATATTTTTAAAAAAAGTAACAGAAAAAGACAAATATTTCTTT

************************************************************

677-730 TTATGCAAAGTTTGAAGTCTAGATACCGATGTTAATTTTTTTTATAAAAAAAATGGAATA

531-730 TTATGCAAAGTTTGAAGTCTAGATACCGATGTTAATTTTTTTTATAAAAAAAATGGAATA

************************************************************

677-730 GGCACCAAAAAGAAAATAAATGATAAAAATAAAATAGAAGAGACAAAAAAAAAAANNNNN

531-730 GGCACCAAAAAGAAAATAAATGATAAAAATAAAATAGAAGAGACAAAAAAAAAAANNNNN

************************************************************

677-730 NNNNNNNNNNNNNNNNNNNNNNNNNNNNNNNNNNNNNNNNNNNNNNNNNNNNNNNNNNNN

531-730 NNNNNNNNNNNNNNNNNNNNNNNNNNNNNNNNNNNNNNNNNNNNNNNNNNNNNNNNNNNN

************************************************************

677-730 NNNNNNNNNNNNNNNNNNNNNNNNNNNNNNNNNNNNNNNNNNNNNAANAAAAAAAAAAAA

531-730 NNNNNNNNNNNNNNNNNNNNNNNNNNNNNNNNNNNNNNNNNNNNNNANAAAAAAAAAAAG

********************************************* *************

677-730 AAAAAAAAAAAAAAAAAAAAAAAAAAAAAAAAAAGAAAAAAAAAAAAATATGGAAACACA

531-730 AAAAAAAAAAAAAAAAAAAAAAAAAAAAAAAAAAAAAAAAAAAAAAAAAATGGAAACACA

********************************** ************* ***********

677-730 GAGTAGTTCCATGGGCAAAATACTAAAACAAATTTTATGAAAAATTCATTCACTCAAAGA

531-730 GAGTAGTTCCATGGGCAAAATACTAAAACAAATTTTATGAAAAATTCATTCACTCAAAGA

************************************************************

677-730 AGAAAAAAGCTTTTCCAAGATTTTATAAGTAAAATCTCAAATAGTTTTGCAAACCTCTAG

531-730 AGAAAAAAGCTTTTCCAAGATTTTATAAGTAAAATCTCAAATAGTTTTGCAAACCTCTAG

************************************************************

677-730 CATACATAATTCAACATGCATTTTTTTCCATTGAGCTATTTCATTAAAAATTTGAATCCT

531-730 CATACATAATTCAACATGCATTTTTTTCCATTGAGCTATTTCATTAAAAATTTGAATCCT

************************************************************

677-730 CATTTTATTAAACTAAAAAAAAAAAATTATAATTATATATAAATAACTTAACAATAGATC

531-730 CATTTTATTAAACTAAAAAAAAAAAATTATAATTATATATAAATAACTTAACAATAGATC

************************************************************

677-730 TAGCCTCATTCGAAAGTTCAAAAATCAAAATAAAATAAGTACGAAGTAAAGTAAAACTCA

531-730 TAGCCTCATTCGAAAGTTCAAAAATCAAAATAAAATAAGTACGAAGTAAAGTAAAACTCA

************************************************************

677-730 CAAGGGTCTCCATGATGAGCGCTAACAAGTCGAAGAGAAACACCACCCCCTAAAAGCTCA

531-730 CAAGGGTCTCCATGATGAGCGCTAACAAGTCGAAGAGAAACACCACCCCCTAAAAGCTCA

************************************************************

677-730 TGAAGATTATCCAGAACAGTGGAACCAAAATCATTGAAGTCCAAAACATTGTCTCTCATC

531-730 TGAAGATTATCCAGAACAGTGGAACCAAAATCATTGAAGTCCAAAACATTGTCTCTCATC

************************************************************

677-730 AAAACCACAGTTCCATGGATTTTCTTCCCTCCATGCTTCAACCCAGAGATAGCCCCCACA

531-730 AAAACCACAGTTCCATGGATTTTCTTCCCTCCATGCTTCAACCCAGAGATAGCCCCCACA

************************************************************

677-730 ATGCTTCCAATCAACAT

531-730 ATGCTTCCAATCAACAT

*****************

**Supplementary Figure S4.** Nucleotide sequence similarity of ClCG02G015730 (730) between resistant parent USVL531-MDR (531) and susceptible parent USVL677-PMS (677). Sequences were aligned using Clustal Omega (<https://www.ebi.ac.uk/jdispatcher/msa/clustalo?stype=dna>).

677-750 TTAAATGGAGACACTGTTGGGAATTCCTTTCCCTGTCAGCCCCTCTTCACTGGATGGATA

531-750 TTAAATGGAGACACTGTTGGGAATTCCTTTCCCTGTCAGCCCCTCTTCACTGGATGGATA

************************************************************

677-750 GAGCAAAGTGTAAGGCATCAGAACCGGTCCAACTCTGTTTCTAAGTGTCAAATCTTCATT

531-750 GAGCAAAGTGTAAGGCATCAGAACCGGTCCAACTCTGTTTCTAAGTGTCAAATCTTCATT

************************************************************

677-750 TCTTTTCATGATTCCATCTTCGATCTCGGCCAACTTTTTCCCGAACTTCTCAAACGCTTC

531-750 TCTTTTCATGATTCCATCTTCGATCTCGGCCAACTTTTTCCCGAACTTCTCAAACGCTTC

************************************************************

677-750 CAGCACTCCTTTGTCTGTAGTCCATTCGGGGGAGTCTCTTTGGCCGAGATAGACCTCATC

531-750 CAGCACTCCTTTGTCTGTAGTCCATTCGGGGGAGTCTCTTTGGCCGAGATAGACCTCATC

************************************************************

677-750 AGACGAATGCCTCGACAAAATCTCTATCAACGAGACGCCAAGAAGAGTTTGAAGTTGTGC

531-750 AGACGAATGCCTCGACAAAATCTCTATCAACGAGACGCCAAGAAGAGTTTGAAGTTGTGC

************************************************************

677-750 AGTGATTGTTCTTAAGAAAGCTTTCTCAGGATCTGATTCGAGTTCTTTATACTCTGGAGT

531-750 AGTGATTGTTCTTAAGAAAGCTTTCTCAGGATCTGATTCGAGTTCTTTATACTCTGGAGT

************************************************************

677-750 GCCTTCTTCCGGCATGAATTTTCGACTTATAGTTGGTCGATTGGGAAGATAACCTGCATA

531-750 GCCTTCTTCCGGCATGAATTTTCGACTTATAGTTGGTCGATTGGGAAGATAACCTGCATA

************************************************************

677-750 AGGGTATTGTCCAAAGTTTACTGCAGCATGAAGAGCTGAAGCAATCCATATGATGATAGT

531-750 AGGGTATTGTCCAAAGTTTACTGCAGCATGAAGAGCTGAAGCAATCCATATGATGATAGT

************************************************************

677-750 ACATGAATGTATTAACTCTTCAACATTCTGCATCTTAGGCCACCATAATTCATCTTTCTT

531-750 ACATGAATGTATTAACTCTTCAACATTCTGCATCTTAGGCCACCATAATTCATCTTTCTT

************************************************************

677-750 GTCACCATGACCTTCCTCTCTAAGTTCCTTCCACCAATACTGAAGTTCCGAGTCATTCTG

531-750 GTCACCATGACCTTCCTCTCTAAGTTCCTTCCACCAATACTGAAGTTCCGAGTCATTCTG

************************************************************

677-750 TACCGTTTCATCAGTCTTGTAATAGAAAGAACAATAATCCGTAACCCATGTCTTGATCGC

531-750 TACCGTTTCATCAGTCTTGTAATAGAAAGAACAATAATCCGTAACCCATGTCTTGATCGC

************************************************************

677-750 TGACCAAATCTCAAGTCCATCGACAGCATATGGATAGTCCTCGATTACAAGACGAAGTCC

531-750 TGACCAAATCTCAAGTCCATCGACAGCATATGGATAGTCCTCGATTACAAGACGAAGTCC

************************************************************

677-750 ATGTGGAGAATTTGTATCCTCAATTGCCATTCCTCTAAACGAAACAATGATTTTGAACAA

531-750 ATGTGGAGAATTTGTATCCTCAATTGCCATTCCTCTAAACGAAACAATGATTTTGAACAA

************************************************************

677-750 GTTAAATGAGTCAATGACTAGTTTGTAAAGTATCAGAATATGTTTAACTTAAAATCTATA

531-750 GTTAAATGAGTCAATGACTAGTTTGTAAAGTATCAGAATATGTTTAACTTAAAATCTATA

************************************************************

677-750 CACAACAGGCTCCTATACTGTATGAAAACAAATTAATCTTTAAACAGACTAGTGCCCGAT

531-750 CACAACAGGCTCCTATACTGTATGAAAACAAATTAATCTTTAAACAGACTAGTGCCCGAT

************************************************************

677-750 ACCAACTACTACTATGAATTTTTTACCTCCTGATGAGATCTGCAGGGAGTGCTTGTTCAT

531-750 ACCAACTACTACTATGAATTTTTTACCTCCTGATGAGATCTGCAGGGAGTGCTTGTTCAT

************************************************************

677-750 GAAAAATCCAGTCCTTATATAAGACAGCTGACATCTCCATGGCATATTTGGATGGAAACA

531-750 GAAAAACCCAGTCCTTATATAAGACAGCTGACATCTCCATGGCATATTTGGATGGAAACA

****** *****************************************************

677-750 CTGTTGCTTCCAAAATGCCACCTGCATTAATGAGTATCTGTCGAGCAAACGCGTTTATAT

531-750 CTGTTGCTTCCAAAATGCCACCTGCATTAATGAGTATCTGTCGAGCAAACGCGTTTATAT

************************************************************

677-750 TCATGGTGTCTCGAAAGTGAGGATGGAGCAGCTTGTAAACTGGATGAAGAACACTTAGTT

531-750 TCATGGTGTCTCGAAAGTGAGGATGGAGCAGCTTGTAAACTGGATGAAGAACACTTAGTT

************************************************************

677-750 GTCGGTTCGTTGCAATCACAAATGGCTCAATTACAGCATGAGTATTCAACCTACATGAAA

531-750 GTCGGTTCGTTGCAATCACAAATGGCTCAATTACAGCATGAGTATTCAACCTACATGAAA

************************************************************

677-750 CCGAAATTATATTCAAAAATCCATATGATTTAGAACTATACACAAATTTTTAGTCCATGT

531-750 CCGAAATTATATTCAAAAATCCATATGATTTAGAACTATACACAAATTTTTAGTCCATGT

************************************************************

677-750 CACAGAAGAGTTATACCAATGGCTGATGAGTTGATGGTAGCCAGAGTCATTTACGGCTGC

531-750 CACAGAAGAGTTATACCAATGGCTGATGAGTTGATGGTAGCCAGAGTCATTTACGGCTGC

************************************************************

677-750 ATAAGCTTTGGCAAGTTGCCAAATTGAACTACCAACCCCTTGTTCAGCTGGAAAGAAAAC

531-750 ATAAGCTTTGGCAAGTTGCCAAATTGAACTACCAACCCCTTGTTCAGCTGGAAAGAAAAC

************************************************************

677-750 CTTGCTAACTGCTCCAAATTCGTCTCCCTGAGGATTTGGCAAGCTCAGTTCGATTGCCAG

531-750 CTTGCTAACTGCTCCAAATTCGTCTCCCTGAGGATTTGGCAAGCTCAGTTCGATTGCCAG

************************************************************

677-750 TGGCTTCAGAGTCCCATTTTCTTTAAGGAAAAGTACGGTTCTGCTGGCATAAGTCTTTGT

531-750 TGGCTTCAGAGTCCCATTTTCTTTAAGGAAAAGTACGGTTCTGCTGGCATAAGTCTTTGT

************************************************************

677-750 GGAAGTTGTATTTATTCGTCTAAGGTATGGCATCAGTGAATCATGGTGATCCAATATGAA

531-750 GGAAGTTGTATTTATTCGTCTAAGGTATGGCATCAGTGAATCATGGTGATCCAATATGAA

************************************************************

677-750 CAACCTGTTCTTCTTGATTGCCTAAACCACCAAATTACACAGAAATATTATCAGCTATGT

531-750 CAACCTGTTCTTCTTGATTGCCTAAACCACCAAATTACACAGAAATATTATCAGCTATGT

************************************************************

677-750 TAATCATACATCTTTTGTTTTCCAAGGATTAAAACTTCATTTTATTTCGATTATGCACTC

531-750 TAATCATACATCTTTTGTTTTCCAAGGATTAAAACTTCATTTTATTTCGATTATGCACTC

************************************************************

677-750 TATGATGATAATTCACCTCTTCTACTGTAAGTCCATCCAAGTTATGTATTATGTGTTCTT

531-750 TATGATGATAATTCACCTCTTCTACTGTAAGTCCATCCAAGTTATGTATTATGTGTTCTT

************************************************************

677-750 CAGTTATCTTACTATTTTGATCCCCAAAAACTTCAGGGTCAAGTTTGCTTGTTGGTGGAA

531-750 CAGTTATCTTACTATTTTGATCCCCAAAAACTTCAGGGTCAAGTTTGCTTGTTGGTGGAA

************************************************************

677-750 ACTCCTGCAATTCAAAGTATGAATATTCAATTCCAGGAGAACAATATCTCTCTCTACACA

531-750 ACTCCTGCAATTCAAAGTATGAATATTCAATTCCAGGAGAACAATATCTCTCTCTACACA

************************************************************

677-750 CGCACAGATAGGCTAAATTAAAAAAAAAAAAAAAAAAAAAAAAAAAAAAAAAAAAAAAAA

531-750 CGCACAGATAGGCTAAATTAAAAAAAAAAAAAAAAAAAAAAAAAAAAAAAAAAAAAAAAA

************************************************************

677-750 AAAAAAANNNNNNNNNNNNNNNNNNNNNNNNNNNNNNNNNNNNNNNNNNNNNNNNNNNNN

531-750 AAAAAAANNNNNNNNNNNNNNNNNNNNNNNNNNNNNNNNNNNNNNNNNNNNNNNNNNNNN

************************************************************

677-750 NNNNNNNNNNNNNNNNNNNNNNNNNNNNNNNNNNNNNNNNNNNNNNNNNNNNNNNNNNNN

531-750 NNNNNNNNNNNNNNNNNNNNNNNNNNNNNNNNNNNNNNNNNNNNNNNNNNNNNNNNNNNN

************************************************************

677-750 NNNNNNNNNNNNNNNNNNNNNNNNNNNNNNNNNNNNNNNNNNNNNNNNNNNNNNNNNNNN

531-750 NNNNNNNNNNNNNNNNNNNNNNNNNNNNNNNNNNNNNNNNNNNNNNNNNNNNNNNNNNNN

************************************************************

677-750 NNNNNNNNNNNNNNNNNNNNNNNNNNNNNNNNNNNNNNNNNNNNNNNNNNNNNNNNNNNN

531-750 NNNNNNNNNNNNNNNNNNNNNNNNNNNNNNNNNNNNNNNNNNNNNNNNNNNNNNNNNNNN

************************************************************

677-750 NNNNNNNNNNNNNNNNNNNNNNNNNNNNNNNNNNNNNNNNNNNNNNNNNNNNNNNNNNNN

531-750 NNNNNNNNNNNNNNNNNNNNNNNNNNNNNNNNNNNNNNNNNNNNNNNNNNNNNNNNNNNN

************************************************************

677-750 NNNNNNNNNNNNNNNNNNNNNNNNNNNNNNNNNNNNNNNNNNNNNNNNNNNNNNNNNNNN

531-750 NNNNNNNNNNNNNNNNNNNNNNNNNNNNNNNNNNNNNNNNNNNNNNNNNNNNNNNNNNNN

************************************************************

677-750 NNNNNNNNNNNNNNNNNNNNNNNNNNNNNNNNNNNNNNNNNNNNNNNNNNNNNNNNNNNN

531-750 NNNNNNNNNNNNNNNNNNNNNNNNNNNNNNNNNNNNNNNNNNNNNNNNNNNNNNNNNNNN

************************************************************

677-750 NNNNNNNNNNNNNNNNNNNNNNNNNNNNNNNNNNNNNNNNNNNNNNNNNNNNNNNNNNNN

531-750 NNNNNNNNNNNNNNNNNNNNNNNNNNNNNNNNNNNNNNNNNNNNNNNNNNNNNNNNNNNN

************************************************************

677-750 NNNNNNNNNNNNNNNNNNNNNNNNNNNNNNNNNNNNNNNNNNNNNNNNNNNNNNNNNNNN

531-750 NNNNNNNNNNNNNNNNNNNNNNNNNNNNNNNNNNNNNNNNNNNNNNNNNNNNNNNNNNNN

************************************************************

677-750 NNNNNNNNNNNNNNNNNNNNNNNNNNNNNNNNNNNNNNNNNNNNNNNNNNNNNNNNNNNN

531-750 NNNNNNNNNNNNNNNNNNNNNNNNNNNNNNNNNNNNNNNNNNNNNNNNNNNNNNNNNNNN

************************************************************

677-750 NNNNNNNNNNNNNNNNNNNNNNNNNNNNNNNNNNNNNNNNNNNNNNNNNNNNNNNNNNNN

531-750 NNNNNNNNNNNNNNNNNNNNNNNNNNNNNNNNNNNNNNNNNNNNNNNNNNNNNNNNNNNN

************************************************************

677-750 NNNNNNNNNNNNNNNNNNNNNNNNNNNNNNNNNNNNNNNNNNNNNNNNNNNNNNNNNNNN

531-750 NNNNNNNNNNNNNNNNNNNNNNNNNNNNNNNNNNNNNNNNNNNNNNNNNNNNNNNNNNNN

************************************************************

677-750 NNNNNNNNNNNNNNNNNNNNNNNNNNNNNNNNNNNNNNNNNNNNNNNNNNNNNNNNNNNN

531-750 NNNNNNNNNNNNNNNNNNNNNNNNNNNNNNNNNNNNNNNNNNNNNNNNNNNNNNNNNNNN

************************************************************

677-750 NNNNNNNNNNNNNNNNNNNNNNNNNNNNNNNNNNNNNNNNNNNNNNNNNNNNNNNNNNNN

531-750 NNNNNNNNNNNNNNNNNNNNNNNNNNNNNNNNNNNNNNNNNNNNNNNNNNNNNNNNNNNN

************************************************************

677-750 NNNNNNNNNNNNNNNNNNNNNNNNNNNNNNNNNNNNNNNNNNNNNNNNNNNNNNNNNNNN

531-750 NNNNNNNNNNNNNNNNNNNNNNNNNNNNNNNNNNNNNNNNNNNNNNNNNNNNNNNNNNNN

************************************************************

677-750 NNNNNNNNNNNNNNNNNNNNNNNNNNNNNNNNNNNNNNNNNNNNNNNNNNNNNNNNNNNN

531-750 NNNNNNNNNNNNNNNNNNNNNNNNNNNNNNNNNNNNNNNNNNNNNNNNNNNNNNNNNNNN

************************************************************

677-750 NNNNNNNNNNNNNNNNNNNNNNNNNNNNNNNNNNNNNNNNNNNNNNNNNNNNNNNNNNNN

531-750 NNNNNNNNNNNNNNNNNNNNNNNNNNNNNNNNNNNNNNNNNNNNNNNNNNNNNNNNNNNN

************************************************************

677-750 NNNNNNNNNNNNNNNNNNNNNNNNNNNNNNNNNNNNNNNNNNNNNNNNNNNNNNNNNNNN

531-750 NNNNNNNNNNNNNNNNNNNNNNNNNNNNNNNNNNNNNNNNNNNNNNNNNNNNNNNNNNNN

************************************************************

677-750 NNNNNNNNNNNNNNNNNNNNNNNNNNNNNNNNNNNNNNNNNNNNNNNNNNNNNNNNNNNN

531-750 NNNNNNNNNNNNNNNNNNNNNNNNNNNNNNNNNNNNNNNNNNNNNNNNNNNNNNNNNNNN

************************************************************

677-750 NNNNNNNNNNNNNNNNNNNNNNNNNNNNNNNNNNNNNNNNNNNNNNNNNNNNNNNNNNNN

531-750 NNNNNNNNNNNNNNNNNNNNNNNNNNNNNNNNNNNNNNNNNNNNNNNNNNNNNNNNNNNN

************************************************************

677-750 NNNNNNNNNNNNNNNNNNNNNNNNNNNNNNNNNNNNNNNNNNNNNNNNNNNNNNNNNNNN

531-750 NNNNNNNNNNNNNNNNNNNNNNNNNNNNNNNNNNNNNNNNNNNNNNNNNNNNNNNNNNNN

************************************************************

677-750 NNNNNNNNNNNNNNNNNNNNNNNNNNNNNNNNNNNNNNNNNNNNNNNNNNNNNNNNNNNN

531-750 NNNNNNNNNNNNNNNNNNNNNNNNNNNNNNNNNNNNNNNNNNNNNNNNNNNNNNNNNNNN

************************************************************

677-750 NNNNNNNNNNNNNNNNNNNNNNNNNNNNNNNNNNNNNNNNNNNNNNNNNNNNNNNNNNNN

531-750 NNNNNNNNNNNNNNNNNNNNNNNNNNNNNNNNNNNNNNNNNNNNNNNNNNNNNNNNNNNN

************************************************************

677-750 NNNNNNNNNNNNNNNNNNNNNNNNNNNNNNNNNNNNNNNNNNNNNNNNNNNNNNNNNNNN

531-750 NNNNNNNNNNNNNNNNNNNNNNNNNNNNNNNNNNNNNNNNNNNNNNNNNNNNNNNNNNNN

************************************************************

677-750 NNNNNNNNNNNNNNNNNNNNNNNNNNNNNNNNNNNNNNNNNNNNNNNNNNNNNNNNNNNN

531-750 NNNNNNNNNNNNNNNNNNNNNNNNNNNNNNNNNNNNNNNNNNNNNNNNNNNNNNNNNNNN

************************************************************

677-750 NNNNNNNNNNNNNNNNNNNNNNNNNNNNNNNNNNNNNNNNNNNNNNNNNNNNNNNNNNNN

531-750 NNNNNNNNNNNNNNNNNNNNNNNNNNNNNNNNNNNNNNNNNNNNNNNNNNNNNNNNNNNN

************************************************************

677-750 NNNNNNNNNNNNNNNNNNNNNNNNNNNNNNNNNNNNNNNNNNNNNNNNNNNNNNNNNNNN

531-750 NNNNNNNNNNNNNNNNNNNNNNNNNNNNNNNNNNNNNNNNNNNNNNNNNNNNNNNNNNNN

************************************************************

677-750 NNNNNNNNNNNNNNNNNNNNNNNNNNNNNNNNNNNNNNNNNNNNNNNNNNNNNNNNNNNN

531-750 NNNNNNNNNNNNNNNNNNNNNNNNNNNNNNNNNNNNNNNNNNNNNNNNNNNNNNNNNNNN

************************************************************

677-750 NNNNNNNNNNNNNNNNNNNNNNNNNNNNNNNNNNNNNNNNNNNNNNNNNNNNNNNNNNNN

531-750 NNNNNNNNNNNNNNNNNNNNNNNNNNNNNNNNNNNNNNNNNNNNNNNNNNNNNNNNNNNN

************************************************************

677-750 NNNNNNNNNNNNNNNNNNNNNNNNNNNNNNNNNNNNNNNNNNNNNNNNNNNNNNNNNNNN

531-750 NNNNNNNNNNNNNNNNNNNNNNNNNNNNNNNNNNNNNNNNNNNNNNNNNNNNNNNNNNNN

************************************************************

677-750 NNNNNNNNNNNNNNNNNNNNNNNNNNNNNNNNNNNNNNNNNNNNNNNNNNNNNNNNNNNN

531-750 NNNNNNNNNNNNNNNNNNNNNNNNNNNNNNNNNNNNNNNNNNNNNNNNNNNNNNNNNNNN

************************************************************

677-750 NNNNNNNNNNNNNNNNNNNNNNNNNNNNNNNNNNNNNNNNNNNNNNNNNNNNNNNNNNNN

531-750 NNNNNNNNNNNNNNNNNNNNNNNNNNNNNNNNNNNNNNNNNNNNNNNNNNNNNNNNNNNN

************************************************************

677-750 NNNNNNNNNNNNNNNNNNNNNNNNNNNNNNNNNNNNNNNNNNNNNAAAAAAAAAAAAAAA

531-750 NNNNNNNNNNNNNNNNNNNNNNNNNNNNNNNNNNNNNNNNNNNNNAAAAAAAAAAAAAAA

************************************************************

677-750 AAAAAAAAAAAAAAAAAAAAAAAAAAAAAAAAAAAACGTTTGTGAAAAATACCCTTAAAC

531-750 AAAAAAAAAAAAAAAAAAAAAAAAAAAAAAAAAAAACGTTTGTGAAAAATACCCTTAAAC

************************************************************

677-750 TTTCAAATGTTTCAAAAATATCCTTACCGTTAATTTTGGATGGAAACCGTTAGTGTTTTG

531-750 TTTCAAATGTTTCAAAAATATCCTTACCGTTAATTTTGGATGGAAACCGTTAGTGTTTTG

************************************************************

677-750 TTTCGAAAATACCCTTGACCTTTCAAAATTAAAAAATTAAAAATTAAAAAAATTAAAAAA

531-750 TTTCGAAAATACCCTTGACCTTTCAAAATTAAAAAATTAAAAATTAAAAAAATTAAAAAA

************************************************************

677-750 AAAACTTAAAGAAAAGCTCAAAAAATAAAAACCTTTACCGTTAATATATGAACAAAAACT

531-750 AAAACTTAAAGAAAAGCTCAAAAAATAAAAACCTTTACCGTTAATATATGAACAAAAACT

************************************************************

677-750 ATTCATACCTCATTGCAAAAATATCTCTAAACTTTCAAAAGTTGCATTAGTACCCTTACC

531-750 ATTCATACCTCATTGCAAAAATATCTCTAAACTTTCAAAAGTTGCATTAGTACCCTTACC

************************************************************

677-750 TTAAAATTTAAAATAATAAAAATGAAAAAGGTTAAAAATGCTTGTTTATCTACACCCCAT

531-750 TTAAAATTTAAAATAATAAAAATGAAAAAGGTTAAAAATGCTTGTTTATCTACACCCCAT

************************************************************

677-750 CACCCTCCTCTTCAATTTCTTTTCTCTCCCCTACTTTAATATATGGCCTAACACTTCTCT

531-750 CACCCTCCTCTTCAATTTCTTTTCTCTCCCCTACTTTAATATATGGCCTAACACTTCTCT

************************************************************

677-750 ATTTCCTCTATTTTTTTCTCCCTTCTTTTCTTATTCCCCTTCTTGTTAATTGTTTGACCT

531-750 ATTTCCTCTATTTTTTTCTCCCTTCTTTTCTTATTCCCCTTCTTGTTAATTGTTTGACCT

************************************************************

677-750 TTGTTTGTCATGAAAAACCACCCCCTTCAATTAAGGTATATAGATTATAAAAAGCAAAAA

531-750 TTGTTTGTCATGAAAAACCACCCCCTTCAATTAAGGTATATAGATTATAAAAAGCAAAAA

************************************************************

677-750 AAAAATAATAATAGAAGCACGGACTTTAAATGTTTTAACATGATTGTACATTAGTAGTTG

531-750 AAAAATAATAATAGAAGCACGGACTTTAAATGTTTTAACATGATTGTACATTAGTAGTTG

************************************************************

677-750 TGTGTATTAATAGCCAAATATGAACTTTTTGTATCTGGCTATTTAGTGTTATGGTAAGAT

531-750 TGTGTATTAATAGCCAAATATGAACTTTTTGTATCTGGCTATTTAGTGTTATGGTAAGAT

************************************************************

677-750 AAGATTTTGATTCTGAAATTTTGTGTTTTAACATGAAATTTTAATGGGTGAAAGAATTGA

531-750 AAGATTTTGATTCTGAAATTTTGTGTTTTAACATGAAATTTTAATGGGTGAAAGAATTGA

************************************************************

677-750 ACAAAAGGGAGAAAAATCGAAATATCTATATGAAAAAATATATATTTTATTGTTAAATTA

531-750 ACAAAAGGGAGAAAAATCGAAATATCTATATGAAAAAATATATATTTTATTGTTAAATTA

************************************************************

677-750 TTCTTATCGAATCCCAGAAAACTTCAAATAAATTGATCACTAACTAACGGCTGAAGCATT

531-750 TTCTTATCGAATCCCAGAAAACTTCAAATAAATTGATCACTAACTAACGGTTGAAGCATT

************************************************** *********

677-750 TTCAAACTTTCTTTTTTTTTTGTAAGACTAAGAGTATTAATGCAACCTTTGAAAGTT

531-750 TTCAAACTTTCTTTTTTTTTTGTAAGACTAAGAGTATTAATGCAACCTTTGAAAGTT

*********************************************************

**Supplementary Figure S5.** Nucleotide sequence similarity of ClCG02G015750 (750) between resistant parent USVL531-MDR (531) and susceptible parent USVL677-PMS (677). Sequences were aligned using Clustal Omega (<https://www.ebi.ac.uk/jdispatcher/msa/clustalo?stype=dna>).

USVL677-PMS PLKMYREKELEKKRGDGRGVPKNWENIYDYDVYNDISDLDSNSTNKPPILGGLKSDFDIH 60

PLKMYREKELEKKRGDGRGVPKNWENIYDYDVYNDISDLDSNSTNKPPILGGLKSDFDIH

USVL531-MDR PLKMYREKELEKKRGDGRGVPKNWENIYDYDVYNDISDLDSNSTNKPPILGGLKSDFDIH 60

USVL677-PMS GLRSVLRDIKDKLKASLGKSPKRLESLKDVYAIYEPRSFFRRGKFPMPQVIEGMCNSFPP 120

GLRSVLRDIKDKLKASLGKSPKRLESLKDVYAIYEPRSFFRRGKFPMPQVIEGMCNSFPP

USVL531-MDR GLRSVLRDIKDKLKASLGKSPKRLESLKDVYAIYEPRSFFRRGKFPMPQVIEGMCNSFPP 120

USVL677-PMS TSNLNPSDYGDQNSKITPKQIMNGMEGLTVDQVI 154

TSNLNPSDYGDQNSKITPKQIMNGMEGLTVDQVI

USVL531-MDR TSNLNPSDYGDQNSKITPKQIMNGMEGLTVDQVI 154

**Supplementary Figure S6.** Amino acid sequences similarity of ClCG02G015710 gene between resistant parent USVL531-MDR and susceptible parent USVL677-PMS. Sequences were aligned using NCBI blastx (<https://blast.ncbi.nlm.nih.gov/>). There was 100% similarity between both proteins.

USVL677-PMS MLNIAATFCKRLNVKDLVTNVPVYSSFSGKDGSSVGLSLLFRRWATKKTAGSTKNGRDSK 60

MLNIAATFCKRLNVKDLVTNVPVYSSFSGKDGSSVGLSLLFRRWATKKTAGSTKNGRDSK

USVL531-MDR MLNIAATFCKRLNVKDLVTNVPVYSSFSGKDGSSVGLSLLFRRWATKKTAGSTKNGRDSK 60

USVL677-PMS PKNLGVKKFGGERVIPGNIIVRQRGTRFHPGDYVGIGKDHTLFALKEGCVKFEKHKLSGR 120

PKNLGVKKFGGERVIPGNIIVRQRGTRFHPGDYVGIGKDHTLFALKEGCVKFEKHKLSGR

USVL531-MDR PKNLGVKKFGGERVIPGNIIVRQRGTRFHPGDYVGIGKDHTLFALKEGCVKFEKHKLSGR 120

USVL677-PMS KWVHVIPKEGHVLHPAYA 138

KWVHVIPKEGHVLHPAYA

USVL531-MDR KWVHVIPKEGHVLHPAYA 138

**Supplementary Figure S7.** Amino acid sequences similarity of ClCG02G015720 gene between resistant parent USVL531-MDR and susceptible parent USVL677-PMS. Sequences were aligned using NCBI blastx (<https://blast.ncbi.nlm.nih.gov/>). There was 100% similarity between both proteins.

USVL677-PMS EDKFAWSTDEEFAREMLAGVNPVVIARLQEFPPTSKLDPNIYGDQTSKITEEHIKDGLDG 60

EDKFAWSTDEEFAREMLAGVNPVVIARLQEFPPTSKLDPNIYGDQTSKITEEHIKDGLDG

USVL531-MDR EDKFAWSTDEEFAREMLAGVNPVVIARLQEFPPTSKLDPNIYGDQTSKITEEHIKDGLDG 60

USVL677-PMS LTVDEVIKHTKNFRVDCLLFNFSLNKICFAVEKNKLYILNHHDSLIPYLRRINTTPTKTY 120

LTVDEVIKHTKNFRVDCLLFNFSLNKICFAVEKNKLYILNHHDSLIPYLRRINTTPTKTY

USVL531-MDR LTVDEVIKHTKNFRVDCLLFNFSLNKICFAVEKNKLYILNHHDSLIPYLRRINTTPTKTY 120

USVL677-PMS ATRTVLFLKNDGTLKPLAIELSLPHPQGDKFGVISRVILPAKTGVDGTIWQLAKAYVTVN 180

ATRTVLFLKNDGTLKPLAIELSLPHPQGDKFGVISRVILPAKTGVDGTIWQLAKAYVTVN

USVL531-MDR ATRTVLFLKNDGTLKPLAIELSLPHPQGDKFGVISRVILPAKTGVDGTIWQLAKAYVTVN 180

USVL677-PMS DTGYHQLISHWLNTHATIEPFVIATNRQLSVLHPIHKLLVPHFRDTMNINALARQSLINA 240

DTGYHQLISHWLNTHATIEPFVIATNRQLSVLHPIHKLLVPHFRDTMNINALARQSLINA

USVL531-MDR DTGYHQLISHWLNTHATIEPFVIATNRQLSVLHPIHKLLVPHFRDTMNINALARQSLINA 240

USVL677-PMS DGIIETTHYPAKYSMELSSFVYKTWVFPQQALPADLIKRGVATEDSSSPHGLQLLIEDYP 300

DGIIETTHYPAKYSMELSSFVYKTWVFPQQALPADLIKRGVATEDSSSPHGLQLLIEDYP

USVL531-MDR DGIIETTHYPAKYSMELSSFVYKTWVFPQQALPADLIKRGVATEDSSSPHGLQLLIEDYP 300

USVL677-PMS YAVDGLEIWSAIKTWVQDYCSFYYKDDQTLHNDTELQSWWKELREKGHADKKDEPWWPKM 360

YAVDGLEIWSAIKTWVQDYCSFYYKDDQTLHNDTELQSWWKELREKGHADKKDEPWWPKM

USVL531-MDR YAVDGLEIWSAIKTWVQDYCSFYYKDDQTLHNDTELQSWWKELREKGHADKKDEPWWPKM 360

USVL677-PMS QSVQDLIQSCTIIIWISSALHAAVNFGQYPYGGFAPNRPSTSRRFLPENGTPDYKELETN 420

QSVQDLIQSCTIIIWISSALHAAVNFGQYPYGGFAPNRPSTSRRFLPENGTPDYKELETN

USVL531-MDR QSVQDLIQSCTIIIWISSALHAAVNFGQYPYGGFAPNRPSTSRRFLPENGTPDYKELETN 420

USVL677-PMS PEKAFLRTITSQLQAL**M**GVSVIEILSRHSSDEVYLGQRSDPEWTLDKEALEAFEKFGKKL 480

PEKAFLRTITSQLQAL**+**GVSVIEILSRHSSDEVYLGQRSDPEWTLDKEALEAFEKFGKKL

USVL531-MDR PEKAFLRTITSQLQAL**V**GVSVIEILSRHSSDEVYLGQRSDPEWTLDKEALEAFEKFGKKL 480

USVL677-PMS AEIEGKIAMRNKDPQLKNRVGPVDMPYTLLFPTSSEGLTGRGIPNSISI 529

AEIEGKIAMRNKDPQLKNRVGPVDMPYTLLFPTSSEGLTGRGIPNSISI

USVL531-MDR AEIEGKIAMRNKDPQLKNRVGPVDMPYTLLFPTSSEGLTGRGIPNSISI 529

**Supplementary Figure S8.** Amino acid sequences similarity of ClCG02G015730 gene between resistant parent USVL531-MDR and susceptible parent USVL677-PMS. Sequences were aligned using NCBI blastx (<https://blast.ncbi.nlm.nih.gov/>). There was one Val ↔Met substitution at the 437 position in susceptible parent USVL677-PMS.
